# Preparation of 4-Allenyloxazolines from (*Z*)-2-En-4-yn-1-ol via Propargyl/Allenyl Isomerization

**DOI:** 10.1021/acs.joc.4c01152

**Published:** 2024-08-23

**Authors:** Shi-Heng Hung, Yu-Min Wang, Yi-Hung Liu, Shiuh-Tzung Liu

**Affiliations:** Department of Chemistry, National Taiwan University, Taipei 106, Taiwan

## Abstract

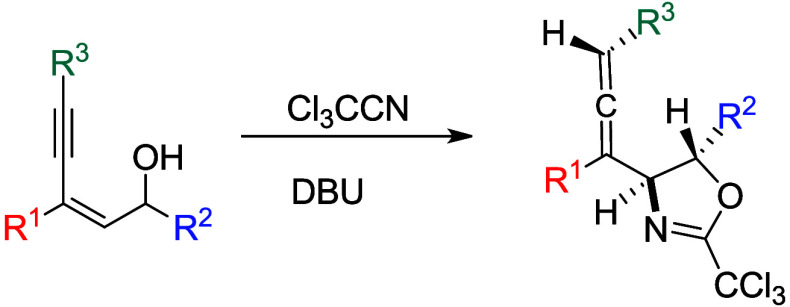

A novel method for the preparation of 4-allenyl-oxazolines **2** is described via the reaction of 2-en-4-yn-1-ols **1** with trichloroacetonitrile in the presence of DBU. Reaction proceeds through the nucleophilic attack of OH functionality in **1** to CCl_3_CN followed by cyclization, propargyl/allene isomerization, and protonation. In this investigation, it is noticed that propargyl/allene isomerization is sensitive to the substituents.

The development of synthetic methods for the construction of allenyl heterocycles has been an attractive subject due to the ability of these heterocycles to engage in cyclization with the allenylic moieties.^1^ Furthermore, quite a few natural products and pharmaceutical molecules embody an allenyl moiety with pendant heterocycles.^2^ Oxazolines are one of the common heterocycles and are useful compounds serving as building blocks in organic synthesis, polymers and as pharmaceuticals.^3^ However, there are a number of reports concerning allenyl-oxazolidinones,^4^ but few with allenyl-oxazoline.^5^

One of the approaches leading to oxazoline rings is the reaction of an allylic alcohol with trichloroactonitrile (Scheme 1A).^6^ The initial nucleophilic attack of the oxygen center on the nitrile generates the imidate anion **I**, which subsequently undergoes a 5-exo closure with the iodiranium moiety in **II** to give the oxazoline product. Although the transformation of 1,3-enynes into allyenyl systems is well-documented,^7^ we imagined that substituted (*Z*)-2-en-4-yn-1-ol **III** might be useful to grant the 4-allenyl oxazolines (Scheme 1B). Presumably, reaction of **III** with CCl_3_CN gives intermediate **IV**, which undergoes propargyl/allenyl isomerization, followed by protonation to reach the final product. However, there is a possibility for the protonation taking place with the initially generated propargylic intermediate **IV** and resulting in the formation of a propargyl-substituted oxazoline.^4c^

**Scheme 1:**
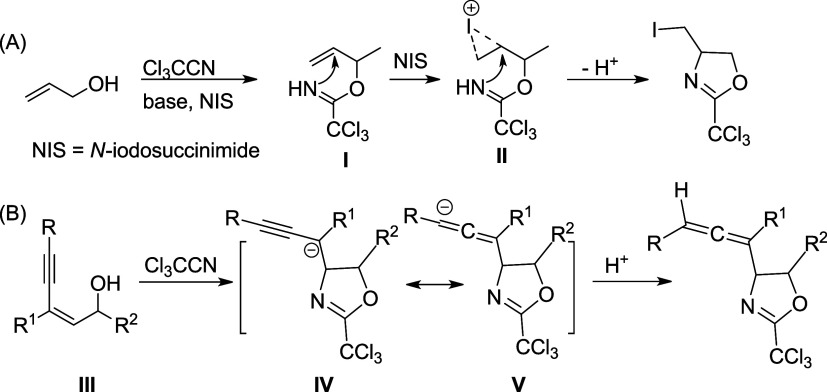
Preparation of Oxazolines from Reaction of Allyl Alcohol with CCl_3_CN

2-En-4-yn-1-ols are useful synthetic intermediates and could be prepared by addition to chalcone followed by acid-catalyzed isomerization as reported in our early works.^8^ This investigation began with the reaction of **1a** with trichloroacetonitrile in the presence of DBU in dichloromethane at ambient temperature (eq 1). To our delight, the desired compound **2a** was obtained in 43%. Compound **2a** was characterized by NMR and ESI-MS spectroscopy. ESI-HRMS gives a signal at [M + H]^+^*m*/*z* = 502.0318, consistent with the molecular formula of C_26_H_19_Cl_4_NO. Three unique signals for H^a^ ∼ H^c^ in ^1^H NMR appeared at δ 6.93 (d, *J* = 2.3 Hz), 5.84 (d, *J* = 7.9 Hz) and 5.29 (dd, *J* = 7.9, 2.3 Hz). Based on the NOE effect between H^a^ and H^b^ as well as the coupling constants,^6c^ the relative configurations along the oxazoline ring and allenylic hydrogen were assigned as shown in the structure **2a** (eq 1). The *trans-*fashion between H^b^ and H^c^ was further confirmed by X-ray crystallography on the hydrolysis product (see below).
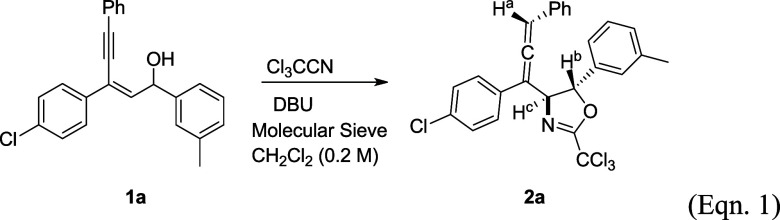
1

Optimization of reaction conditions for better production of **2a** was screened (Table 1). Running the reaction in CH_2_Cl_2_ without the addition of molecular sieves did not provide the desired product (entry 2), indicating the interference of moisture in the reaction. Screening various amounts of reagent and base (entries 3–7), it was found that the combination of CCl_3_CN (2 equiv) and DBU (1.5 equiv) provided a quantitative yield, which appears to be the best choice (entry 5). Reaction without the presence of DBU or the use of a catalytic amount of DBU provided unsatisfactory results (entry 7–8), indicating that the base is essential for the reaction. Other bases and solvents were also screened (entries 10–13), but the results were no better than the conditions shown in entry 5.

**Table 1:** Reaction Optimization^a^

entry	CCl_3_CN (equiv)	Base (equiv)	Solvent	Yield (%)^b^
1	1.5	DBU (1.5)	CH_2_Cl_2_	53 (43)
2^c^	1.5	DBU (1.5)	CH_2_Cl_2_	0
3	1.0	DBU (1.5)	CH_2_Cl_2_	50
4	1.0	DBU (1.0)	CH_2_Cl_2_	38
5	**2.0**	**DBU (1.5)**	**CH**_**2**_**Cl**_**2**_	99 (96)
6	2.0	DBU (1.0)	CH_2_Cl_2_	82
7	2.0	DBU (0.2)	CH_2_Cl_2_	28
8	2.0	–	CH_2_Cl_2_	0
9	2.0	DBU (1.5)	toluene	84
10	2.0	Pyridine (1.5)	CH_2_Cl_2_	NR
11	2.0	*t*BuOK (1.5)	CH_2_Cl_2_	d
12	2.0	KOAc	CH_2_Cl_2_	d
13	2.0	KOH	CH_2_Cl_2_	d

a Reaction condition: **1a** (0.2 mmol), CCl_3_CN, 4 Å MS and Base in 1 mL DCM were stirred at ambient temperature for 24 h.

b NMR isolated yields given in parentheses.

c No molecular sieve.

d Complicated mixture

With the optimized conditions, we next studied the reaction scope with various substituents on the substrates (Table 2). Reactions of various substituted aryl groups at R^2^ in 2-en-4-yn-1-ols (**1a**–**1f**) with CCl_3_CN in the presence of DBU gave the cyclized products **2a**–**2f** in excellent yields except for the nitro substituent at the *para* position of the R^2^ group. Presumably, the strong electron withdrawing nature slows down the nucleophilic attack toward the nitrile substrate. However, when R^1^ groups are in various natures of substituents, the reactivity leading to the desired allenyl-oxazolines is completely different. When R^1^ is a *p-*fluorophenyl group in **1g**, the reaction proceeded smoothly to grant the expected product **2g** in 94% yield, and reactant **1h** (R^1^ = *p-*bromophenyl) behaved similarly, giving **2h** in 98% yield. To our surprise, substrate **1i** (R^1^ = *p*-nitrophenyl) did undergo the formation of oxazoline ring but did not form the allenyl product. Apparently, the isomerization of propargyl into the allenyl group did not take place; instead, the propargyl intermediate underwent the abstraction of chlorinium ion from CCl_3_CN to generate **3** as the major product.^9^ For substrates with R^1^ = *p-*MeC_6_H_4_ or *p-*MeOC_6_H_4_ in **1j**–**1k**, reactions went into a complicated mixture and were not able to identify the product. Possibly, the electron-donating nature makes C=C less electrophilic, thus inhibiting the nucleophilic attack for the ring formation. This observation was also found in compound **1p** with the *o*-tolyl group (R^1^ = *o-*MeC_6_H_4_). Interestingly, when R^1^ is a methyl group, substrate **1l** went through a different pathway giving amide **4** in 64% yield. We assumed that the initial imidate anion was not able to undergo nucleophilic ring formation. Alternatively, Overman rearrangement followed by tautomerization took place to give **4** as the final product (Scheme 2).^10^ Finally, the reactivity of two substrates with a different substituent at the R^3^ position was investigated. As a TMS group seated at R^3^, the reaction proceeded similarly to that of **1i**, but providing a protonation product **5**, not a chloride. The trimethylsilyl is known to be a good inductive electron releasing group,^11^ which increases the basicity of propargyl anion to accept a proton. When R^3^ came to be a butyl group, the reaction went to a mixture of unidentified compounds. Besides **1l** (R^3^ = Ph), when the R^3^ group is a *p*-methylphenyl group in **1o**, the expected product **2o** was obtained in 33% yield, similar to that for **2d**. For gram scale reactions, substrates **1a** (1.076 g) and **1h** (1.044 g) were subjected to the reaction under the optimized conditions to give **2a** (1.449 g) and **2h** (0.910 g) in yields of 96% and 78%, respectively, showing the practicality and applicability of the developed method.

**Scheme 2:**
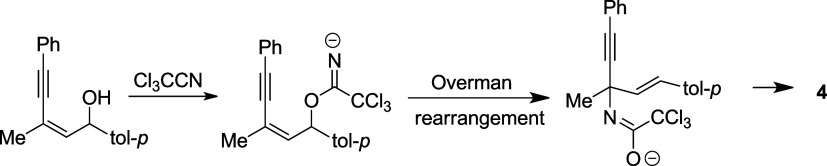
Pathway Leading to Compound **4**

**Table 2:**
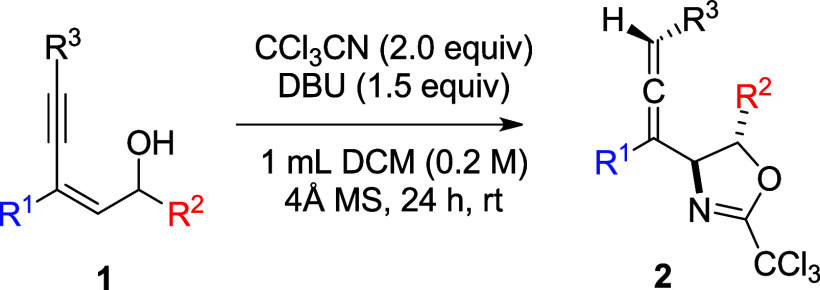

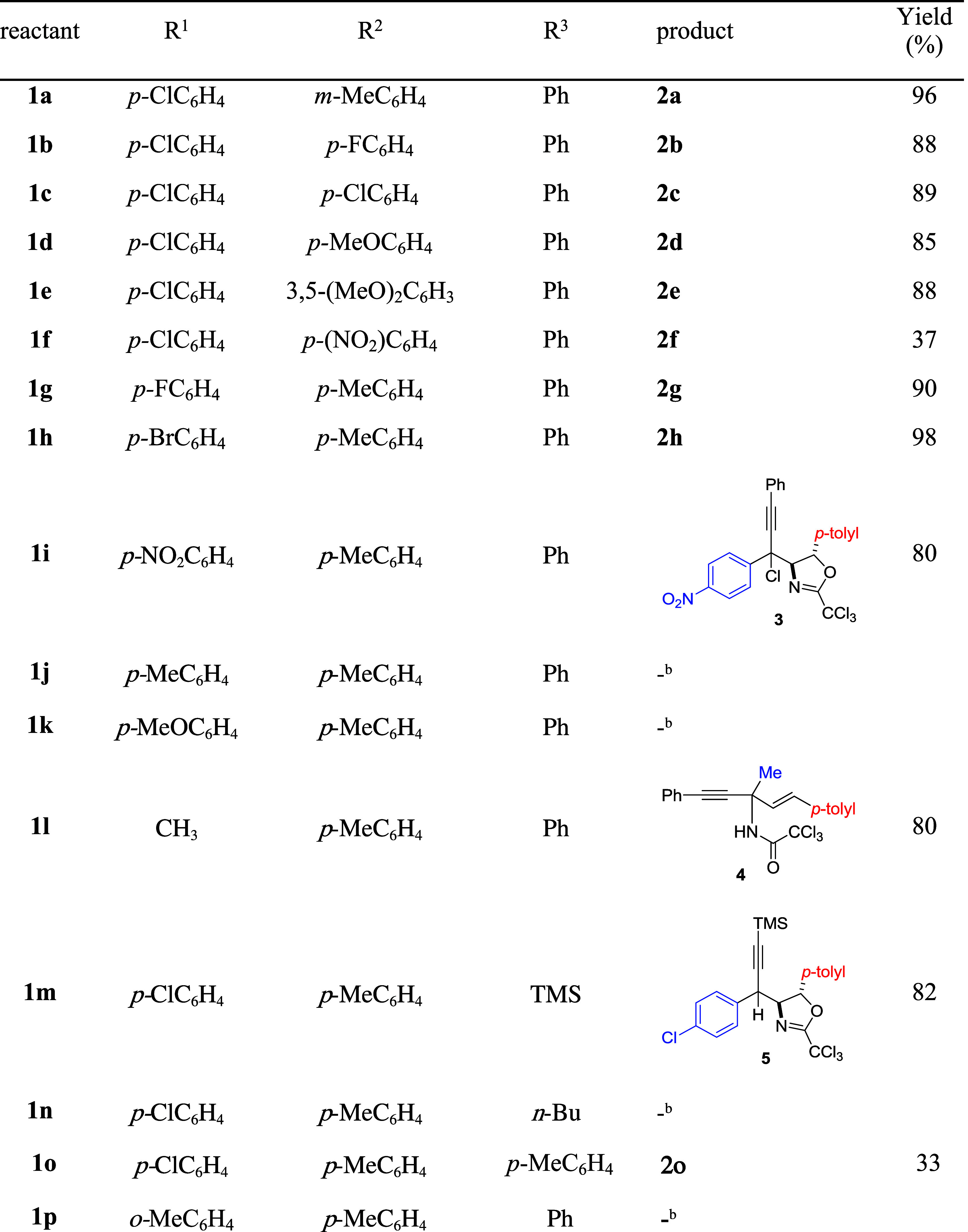
Reaction Scope^a^

a Reaction conditions: A solution of **1** (0.2 mmol), DBU (1.5 equiv) and CCl_3_CN (2 equiv) and 4 Å MS in 1 mL CH_2_Cl_2_ was stirred at room temperature for 24 h. Isolated yield.

b Complicated mixture.

Hydrolysis of 2-(trichloromethyl)-4,5-dihydrooxazoles leading to trichloroacetamido-alcohols is a well-documented reaction.^12^ We selected several compounds **2** for hydrolysis under acidic conditions (Table 3). All reactions provided the desired products quantitatively and all obtained compounds are in solid form. Particularly, crystal structures of both **6c** and **6h** were obtained. Figure 1 illustrates the ORTEP plot of the molecule. The relative configuration at both C4 and C5 are consistent with the proposed structure by NMR spectroscopic analysis. Crystallographic details and an ORTEP plot of **6c** are deposited in the Supporting Information.

**Figure 1:**
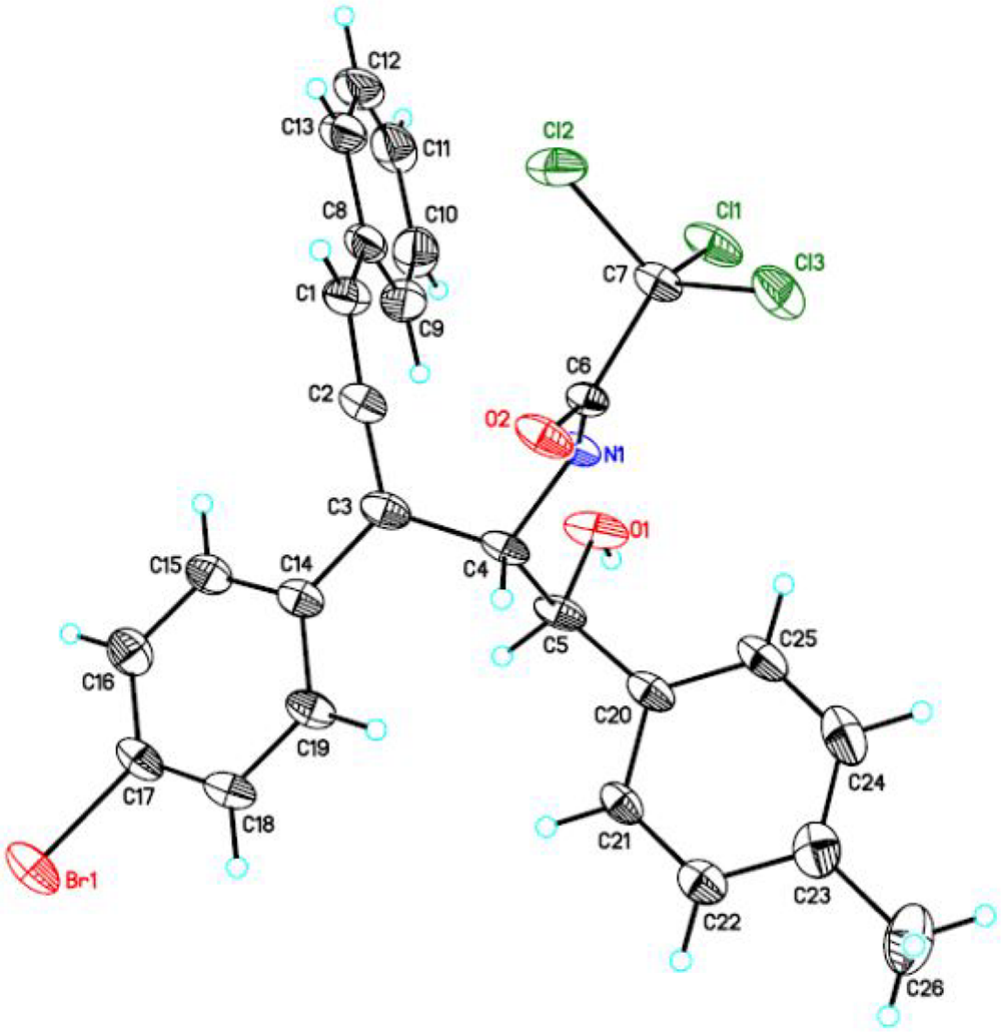
ORTEP plot of **6h** (30% probability ellipsoids)

**Table 3:**
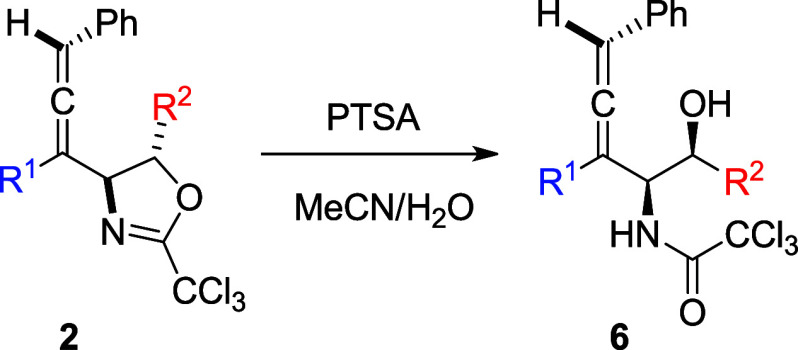
Hydrolysis of **2** under Acidic Conditions^a^

Reactant	R^1^	R^2^	Product	Yield (%)
**2a**	*p-*ClC_6_H_4_	*m*-MeC_6_H_4_	**6a**	99
**2b**	*p-*ClC_6_H_4_	*p-*FC_6_H_4_	**6b**	99
**2c**	*p-*ClC_6_H_4_	*p-*ClC_6_H_4_	**6c**	99
**2h**	*p-*BrC_6_H_4_	*p-*MeC_6_H_4_	**6h**	99

a Reaction conditions: A solution of **2** (0.2 mmol) and *p*-toluenesulfonic acid (0.2 mmol) in a mixture of MeCN (1.6 mL) and H_2_O (0.4 mL) was stirred at rt under nitrogen atmosphere for 0.5 h; isolated yield.

A possible reaction pathway is illustrated in Scheme 3. Nucleophilic attack of **1** with CCl_3_CN in the presence of DBU gives the intermediate **Int-1**, where R^2^ is seated at the pseudo equatorial position. This conformation is more stable than that of **Int-2** with R^2^ in a pseudo axial position, which may cause the steric interaction with the alkynyl group. Intramolecular ring closure followed by propargyl/allenyl isomerization leads to **Int-1′**, which accepts a proton to give the final product **2**.

**Scheme 3:**
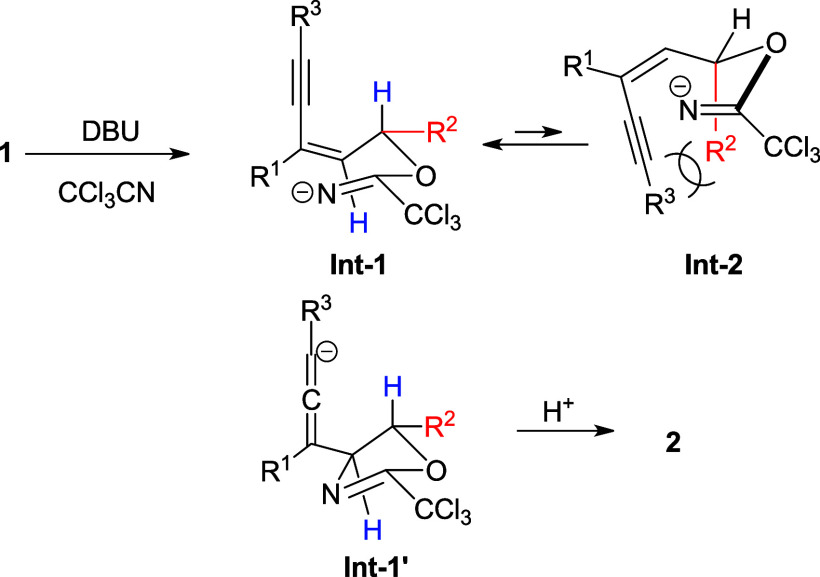
Possible Reaction Pathway

In summary, we have disclosed an efficient method to prepare 4-allenyl-oxazolines from readily accessible 2-en-4-yn-1-ols. Although there is a limitation of substituents in the molecules, it offers an easy way to prepare the desired allene-heterocycles. From the study of the reaction scope, we found that the nature of substituents affects the propargyl/allene isomerization dramatically. In our opinion, theoretical study of the reaction pathway including the activation energy might be the possible way to understand this query and is currently under investigation.

## Experimental Section

### General Information

^1^H and ^13^C NMR spectra were recorded in a 400 MHz or 800 MHz spectrometer in CDCl_3_ referenced to TMS. All chemicals were commercially purchased and used without further purification. Flash chromatography was performed using silica gel 230–400 mesh. Chalcone derivatives were prepared according to the literature procedure. In cases of known compounds, their spectral data were compared with the literature values. Melting points were determined on a Fargo MP-1D instrument. Unless otherwise noted, all the reactions were performed without any special precautions. Compounds **1** were prepared according to our previously reported method^8^ and spectral data are deposited in SI.

### General Procedure for Preparation of **2**

A solution of pent-2-en-4-yn-1-ols **1** (0.2 mmol), 1,8-diazabicyclo[5.4.0]undec-7-ene (DBU, 45 μL, 0.3 mmol, 1.5 equiv) and 4 Å MS in CH_2_Cl_2_ (1 mL) was stirred for 10 min. Trichloroacetonitrile (40 μL, 0.4 mmol, 2 equiv) was then added, and the reaction mixture was stirred at ambient temperature overnight. Saturated NaHCO_3_aqueous solution (10 mL) was added to quench the reaction. Upon extraction with Et_2_O (15 mL × 2), the organic extracts were washed with water (15 mL), brine (15 mL), dried over MgSO_4_ and concentrated. The residue was chromatographed on silica gel with an elution of dichloromethane/hexane.

4-(1-(4-Chlorophenyl)-3-phenylpropa-1,2-dien-1-yl)-5-(*m*-tolyl)-2-(trichloromethyl)-4,5-dihydrooxazole (**2a**). Pale-yellow oil (96.6 mg, 96%). Eluent: hexane/CH_2_Cl_2_ (10:1). ^1^H NMR (400 MHz, CDCl_3_): δ 7.52 (d, *J* = 8.6 Hz, 2H), 7.39–7.36 (m, 4H), 7.34–7.30 (m, 3H), 7.27 (d, *J* = 7.7 Hz, 1H), 7.20–7.18 (m, 1H), 7.13–7.10 (m, 1H), 7.05–7.04 (m, 1H), 6.93 (d, *J* = 2.3 Hz, 1H), 5.84 (d, *J* = 7.9 Hz, 1H), 5.29 (dd, *J* = 7.9, 2.3 Hz, 1H), 2.30 (s, 3H); ^13^C{^1^H} NMR (100 MHz, CDCl_3_): δ 206.2, 162.3, 142.2, 138.8, 138.3, 133.6, 132.4, 132.3, 129.8, 128.9, 128.8, 128.2, 128.1, 127.1, 126.7, 122.9, 109.8, 102.3, 89.4, 74.7(2C), 21.2. HRMS (ESI-TOF) *m*/*z* [M + H]^+^ Calcd for C_26_H_20_Cl_4_NO: 502.0294, found 502.0318.

4-(1-(4-Chlorophenyl)-3-phenylpropa-1,2-dien-1-yl)-5-(*p*-fluorophenyl)-2-(trichloromethyl)-4,5-dihydrooxazole (**2b**). Pale-yellow oil (89.3 mg, 88%). Eluent: hexane/CH_2_Cl_2_ (10:1). ^1^H NMR (400 MHz, CDCl_3_): δ 7.50 (dd, *J* = 8.6, 1.3 Hz, 2H), 7.41–7.26 (m, 10H), 7.08 (t, *J* = 8.6 Hz, 2H), 6.94 (br, 1H), 5.86 (dd, *J* = 7.6, 1.2 Hz, 1H), 5.27 (dd, *J* = 7.6, 1.6 Hz, 1H); ^13^C{^1^H} NMR (100 MHz, CDCl_3_): δ 206.1, 163.0 (d, ^2^*J*_C–F_ = 248.6 Hz), 134.2, 133.8, 132.3 (d, ^4^*J*_C–F_ = 2.5 Hz) 129.0, 128.9, 128.2, 128.1, 128.0 (d, ^3^*J*_C–F_ = 8.3 Hz), 127.1, 116.1, 115.9, 109.7, 102.4, 88.7, 86.4, 74.8(2C); ^19^F NMR (375 MHz, CDCl_3_): δ −111.8. HRMS (ESI-TOF) *m*/*z* [M + H]^+^ Calcd for C_25_H_17_Cl_4_FNO: 506.0043, found: 506.0058.

4-(1-(4-Chlorophenyl)-3-phenylpropa-1,2-dien-1-yl)-5-(*p*-chlorophenyl)-2-(trichloromethyl)-4,5-dihydrooxazole (**2c**). Yellow oil (93.2 mg, 89%). Eluent: hexane/CH_2_Cl_2_ (10:1). ^1^H NMR (400 MHz, CDCl_3_): δ 7.50 (d, *J* = 8.6 Hz, 2H), 7.39–7.32 (m, 10H), 7.22 (d, *J* = 8.4 Hz, 2H), 6.93 (d, *J* = 2.3 Hz, 1H), 5.84 (d, *J* = 7.6 Hz, 1H), 5.25 (dd, *J* = 7.6, 2.3 Hz, 1H); ^13^C{^1^H} NMR (100 MHz, CDCl_3_): δ 206.1, 162.2, 136.9, 135.0, 133.8, 132.2(2C), 129.2, 128.9, 128.9, 128.2, 128.1, 127.2, 127.1, 109.6, 102.4, 88.5, 74.8(2C). HRMS (ESI-TOF) *m*/*z* [M + H]^+^ Calcd for C_25_H_17_Cl_5_NO: 521.9747, found 521.9753.

4-(1-(4-Chlorophenyl)-3-phenylpropa-1,2-dien-1-yl)-5-(*p*-methoxyphenyl)-2-(trichloromethyl)-4,5-dihydrooxazole (**2d**). Pale-yellow oil (88.3 mg, 85%). Eluent: hexane/CH_2_Cl_2_ (10:1). ^1^H NMR (400 MHz, CDCl_3_): δ 7.47 (d, *J* = 8.6 Hz, 2H), 7.38–7.36 (m, 4H), 7.33–7.30 (m, 3H), 7.23 (d, *J* = 8.8 Hz, 2H), 6.92–6.90 (m, 3H), 5.81 (d, *J* = 7.5 Hz, 1H), 5.29 (dd, *J* = 7.5, 2.2 Hz, 1H), 3.84 (s, 3H); ^13^C{^1^H} NMR (100 MHz, CDCl_3_): δ 206.2, 162.4, 160.2, 133.6, 132.4(2C), 130.3, 128.9, 128.8, 128.1, 128.0, 127.7, 127.1, 114.4, 109.9, 102.2, 89.6, 74.5(2C), 55.3; HRMS (ESI-TOF) *m*/*z* [M + H]^+^ Calcd for C_26_H_20_Cl_4_NO_2_: 518.0243, found 518.0244.

4-(1-(4-Chlorophenyl)-3-phenylpropa-1,2-dien-1-yl)-5-(3,5-dimethoxyphenyl)-2-(trichloromethyl)-4,5-dihydrooxazole (**2e**). Pale-yellow oil (96.7 mg, 88%). Eluent: hexane/CH_2_Cl_2_ (10:1). ^1^H NMR (400 MHz, CDCl_3_): δ 7.53 (d, *J* = 8.6 Hz, 2H), 7.38–7.37 (m, 4H), 7.35–7.32 (m, 3H), 6.92 (d, *J* = 2.3 Hz, 1H), 6.46 (t, *J* = 2.2 Hz, 1H), 6.43 (d, *J* = 2.2 Hz, 2H), 5.82 (d, *J* = 7.3 Hz, 1H), 5.30 (dd, *J* = 7.3, 2.3 Hz, 1H), 3.72 (s, 6H); ^13^C{^1^H} NMR (100 MHz, CDCl_3_): δ 206.2, 162.2, 161.2, 140.9, 133.7, 132.3, 132.3, 128.9, 128.8, 128.2, 128.1, 127.1, 110.0, 103.4, 102.3, 101.0, 89.1, 74.7(2C), 55.2; HRMS (ESI-TOF) *m*/*z* [M + H]^+^ Calcd for C_27_H_22_Cl_4_NO_3_, 548.0348, found 548.0363.

4-(1-(4-Chlorophenyl)-3-phenylpropa-1,2-dien-1-yl)-5-(*p*-nitrophenyl)-2-(trichloromethyl)-4,5-dihydrooxazole (**2f**). Yellow oil (39.5 mg, 37%). Eluent: hexane/CH_2_Cl_2_ (10:1). ^1^H NMR (400 MHz, CDCl_3_): δ 8.23 (d, *J* = 8.8 Hz, 2H), 7.54 (d, *J* = 8.6 Hz, 2H), 7.44 (d, *J* = 8.6 Hz, 2H), 7.42–7.37 (m, 5H), 7.35 (d, *J* = 8.6 Hz, 2H), 6.98 (d, *J* = 2.2 Hz, 1H), 5.99 (d, *J* = 7.8 Hz, 2H), 5.25 (d, *J* = 7.8, 2.2 Hz, 1H); ^13^C{^1^H} NMR (100 MHz, CDCl_3_): δ 206.1, 162.1, 148.1, 145.3, 134.0, 131.9 (2C), 129.1, 128.9, 128.5, 128.2, 127.1, 126.4, 124.2, 109.4, 102.7, 87.5, 75.0(2C); HRMS (ESI-TOF) *m*/*z* [M + H]^+^ Calcd for C_25_H_17_Cl_4_N_2_O_3_: 532.9988, found 532.9981.

4-(1-(4-Fluorophenyl)-3-phenylpropa-1,2-dien-1-yl)-5-(*p*-methylphenyl)-2-(trichloromethyl)-4,5-dihydrooxazole (**2g**). Pale-yellow oil (87.6 mg, 90%). Eluent: hexane/CH_2_Cl_2_ (10:1). ^1^H NMR (400 MHz, CDCl_3_): δ 7.52 (dd, *J* = 8.8, 5.3 Hz, 2H), 7.37–7.31 (m, 5H), 7.22–7.18 (m, 4H), 7.04 (t, *J* = 8.7 Hz, 2H), 6.89 (d, *J* = 2.0 Hz, 1H), 5.83 (d, *J* = 7.4 Hz, 1H), 5.29 (7.4, 2.0 Hz, 1H), 2.38 (s, 3H); ^13^C{^1^H} NMR (100 MHz, CDCl_3_): δ 206.0, 162.3, 139.1, 135.5, 132.6, 129.9, 129.6, 128.8, 128.6, 128.5, 127.9, 127.1, 126.0, 115.7, 115.5, 109.9, 102.0, 89.5, 74.9, 21.1; ^19^F NMR (375 MHz, CDCl_3_): δ −114.7. HRMS (ESI-TOF) *m*/*z* [M + H]^+^ Calcd for C_26_H_20_Cl_3_FNO: 486.0589, found 486.0591.

4-(1-(4-Bromophenyl)-3-phenylpropa-1,2-dien-1-yl)-5-(*p*-methylphenyl)-2-(trichloromethyl)-4,5-dihydrooxazole (**2h**). Pale-yellow oil (107.3 mg, 98%). Eluent: hexane/CH_2_Cl_2_ (10:1). ^1^H NMR (400 MHz, CDCl_3_): δ 7.48 (d, *J* = 8.8 Hz, 2H), 7.42 (d, *J* = 8.8 Hz, 2H), 7.39–7.30 (m, 5H), 7.20 (s, 4H), 6.91 (d, *J* = 2.2 Hz, 1H), 5.84 (d, *J* = 7.4 Hz, 1H), 5.30 (dd, *J* = 7.4, 2.2 Hz, 1H), 2.39 (s, 3H); ^13^C{^1^H} NMR (100 MHz, CDCl_3_): δ 206.5, 162.7, 139.5, 135.7, 133.2, 132.6, 132.1, 130.0, 129.2, 128.7, 128.4, 127.4, 126.4, 122.1, 110.3, 102.7, 89.9, 74.9 (2C), 21.5; HRMS (ESI-TOF) *m*/*z* [M + H]^+^ Calcd for C_26_H_20_Cl_3_BrNO: 545.9788, found 545.9745.

4-(1-(4-Chlorophenyl)-3-(p-tolyl)propa-1,2-dien-1-yl)-5-(*p*-tolyl)-2-(trichloromethyl)-4,5-dihydrooxazole (**2o**). Light yellow oil (34.1 mg, 33%). Eluent: hexane/CH_2_Cl_2_ (10:1).^1^H NMR (400 MHz, CDCl_3_): δ7.43 (d, *J* = 8.6 Hz, 2H), 7.26 (d, *J* = 8.7 Hz, 2H), 7.22 (d, *J* = 8.1 Hz, 2H), 7.17–7.12 (m, 6H), 6.85 (d, *J* = 2.1 Hz, 1H), 5.78 (d, *J* = 7.4 Hz, 1H), 5.23 (dd, *J* = 7.4 Hz, *J* = 2.1 Hz, 1H), 2.35 (s, 3H), 2.34 (s, 3H); ^13^C{^1^H} NMR (100 MHz, CDCl_3_): δ 206.1, 139.1, 138.1 (2C), 135.6, 133.5, 132.7, 129.7 (2C), 129.4, 128.8, 128.2, 127.1, 126.0, 109.8, 102.2, 89.6, 86.6, 74.8, 21.3, 21.2. HRMS (ESI-TOF) *m*/*z* [M + H]^+^ Calcd for C_27_H_22_Cl_4_NO: 516.0450, found 516.0448.

4-(1-Chloro-1-(4-nitrophenyl)-3-phenylprop-2-yn-1-yl)-5-(*p*-tolyl)-2-(trichloromethyl)-4,5-dihydrooxazole (**3**). Yellow oil (87.7 mg, 80%). Eluent: hexane/CH_2_Cl_2_ (10:1). ^1^H NMR (400 MHz, CDCl_3_): δ 8.29 (d, *J* = 9.0 Hz, 2H), 8.03 (d, *J* = 9.0 Hz, 2H), 7.63–7.61 (m, 2H), 7.50–7.38 (m, 5H), 7.29 (d, *J* = 8.0 Hz, 2H), 6.34 (d, *J* = 5.4 Hz, 1H), 4.92 (d, *J* = 5.4 Hz, 1H), 2.43 (s, 3H); ^13^C{^1^H} NMR (100 MHz, CDCl_3_): δ 164.8, 148.0, 145.4, 139.2, 135.7, 132.1, 129.8, 128.8, 128.4, 125.9, 123.4, 120.6, 91.6, 87.9, 86.2, 85.0, 83.5, 67.3, 21.2; HRMS (ESI-TOF) *m*/*z* [M + H]^+^ Calcd for C_26_H_19_Cl_4_N_2_O_3_: 547.0144, found 547.0158. The isotope pattern with Cl_4_ also agreed with theoretical analysis (see SI).

2,2,2-Trichloro-*N*-(3-methyl-5-phenyl-1-(*p*-tolyl)pent-1-en-4-yn-3-yl)acetamide (**4**). Off-white solid. (65.1 mg, 80%). Eluent: hexane/CH_2_Cl_2_ (9:1). mp 142–143 °C. ^1^H NMR (400 MHz, CDCl_3_): δ 7.55–7.53 (m, 2H), 7.38–7.36 (m, 5H), 7.17 (d, *J* = 7.8 Hz, 2H), 7.00 (d, *J* = 15.8 Hz, 1H), 6.99 (br, 1H), 6.50 (d, *J* = 15.8 Hz, 1H), 2.37 (s, 3H), 2.02 (s, 3H); ^13^C{^1^H} NMR (100 MHz, CDCl_3_): δ 159.7, 138.1, 133.0, 131.8, 131.3, 129.2, 128.7, 128.2, 127.8, 126.8, 122.0, 87.9, 85.4, 54.6 (2C), 27.5, 21.1; HRMS (ESI-TOF) *m*/*z* [M + H]^+^ Calcd for C_21_H_19_Cl_3_NO: 406.0527, found 406.0538.

4-(1-(4-Chlorophenyl)-3-(trimethylsilyl)prop-2-yn-1-yl)-5-(*p*-tolyl)-2-(trichloromethyl)-4,5-dihydrooxazole (**5**). Yellow oil (81.9 mg, 82%). Eluent: hexane/CH_2_Cl_2_ (9:1). ^1^H NMR (400 MHz, CDCl_3_): δ 7.40 (d, *J* = 8.7 Hz, 2H), 7.36 (d, *J* = 8.7 Hz, 2H), 7.07 (d, *J* = 7.0 Hz, 2H), 6.79 (d, *J* = 8.0 Hz, 2H), 5.74 (d, *J* = 5.6 Hz, 1H), 4.41 (t, *J* = 5.6 Hz, 1H), 4.35 (d, *J* = 5.6 Hz, 1H), 2.32 (s, 3H), 0.25 (s, 9H); ^13^C{^1^H} NMR (100 MHz, CDCl_3_): δ 163.0, 138.3, 136.3, 134.7, 133.6, 129.5, 129.4, 128.8, 124.8, 101.4, 92.0, 86.4, 79.8 (2C), 42.8, 21.0, −0.1; HRMS (ESI-TOF) *m*/*z* [M + H]^+^ Calcd for C_23_H_24_Cl_4_NOSi: 498.0376, found 498.0382.

### General Procedure for Hydrolysis of **2**

To a solution of 4-allenyl-2-oxazoline **2** (0.2 mmol) in acetonitrile (1.2 mL) under a N_2_ atmosphere was added a solution of *para*-toluenesulfonic acid (1 equiv) in 0.8 mL of MeCN/H_2_O (v/v = 1:1). The reaction was stirred at ambient temperature for 0.5 h. A saturated NaHCO_3_ aqueous solution (10 mL) followed by ether (10 mL) was added. The organic portion was separated, and the aqueous portion was extracted with Et_2_O (10 mL). The combined organic extracts were washed with water (10 mL) and brine (10 mL), dried over MgSO_4_ and concentrated. The residue was purified by flash chromatography with elution of EtOAc/hexane to give the desired product.

2,2,2-Trichloro-*N*-(3-(4-chlorophenyl)-1-hydroxy-5-phenyl-1-(*m*-tolyl)penta-3,4-dien-2-yl)acetamide (**6a**). Off-white sold (103.2 mg, 99%). Eluent: EtOAc: hexane (1:9). mp 164–165 °C. ^1^H NMR (400 MHz, CDCl_3_): δ 7.36–7.25 (m, 10H), 7.23 (d, *J* = 6.9 Hz, 1H), 7.17 (d, *J* = 5.4 Hz, 1H), 7.14 (d, *J* = 7.7 Hz, 1H), 7.07 (s, 1H), 7.02 (d, *J* = 7.4 Hz, 1H), 6.68 (d, *J* = 2.6 Hz, 1H), 5.11 (dt, *J* = 8.9, 2.6 Hz, 1H), 4.97 (br, 1H), 2.23 (s, 3H). ^13^C{^1^H} NMR (100 MHz, CDCl_3_): δ 205.9, 161.6, 140.2, 138.6, 134.1, 133.3, 132.8, 129.4, 129.4, 129.3, 128.9, 128.3, 128.3, 127.4, 126.7, 123.1, 109.7, 101.7, 72.9 (2C), 56.5, 21.7. HRMS (ESI-TOF) *m*/*z* [M + Na]^+^ Calcd for C_26_H_21_Cl_4_NO_2_Na: 542.0219, found 542.0200.

2,2,2-Trichloro-*N*-(3-(4-chlorophenyl)-1-hydroxy-5-phenyl-1-(*p*-fluorophenyl)penta-3,4-dien-2-yl)acetamide (**6b**). Off-white sold (104.0 mg, 99%). Eluent: EtOAc: hexane (1:9). mp 161–162 °C. ^1^H NMR (400 MHz, CDCl_3_): δ 7.45 (d, *J* = 8.6 Hz, 2H), 7.43–7.30 (m, 10H), 7.04 (t, *J* = 8.6 Hz, 2H), 6.80 (d, *J* = 2.6 Hz, 1H), 5.23 (dt, *J* = 9.1, 2.6 Hz, 1H), 5.11 (br, 1H), 2.44 (d, *J* = 3.2 Hz, 1H); ^13^C{^1^H} NMR (100 MHz, CDCl_3_): δ 205.5, 162.5 (d, ^1^*J*_C–F_ = 245.9 Hz), 161.4, 135.7 (d, ^4^*J*_C–F_ = 2.7 Hz), 134.0, 132.8, 132.3, 129.1, 129.0, 128.1, 127.9, 127.4 (d, ^3^*J*_C–F_ = 8.2 Hz), 127.0, 115.5 (d, ^2^*J*_C–F_ = 21.7 Hz), 109.3, 101.5, 92.3, 72.0, 56.2; HRMS (ESI-TOF) *m*/*z* [M + Na]^+^ Calcd for C_25_H_18_Cl_4_FNO_2_Na: 545.9968, found 545.9945.

2,2,2-Trichloro-*N*-(3-(4-chlorophenyl)-1-hydroxy-5-phenyl-1-(*p*-chlorophenyl)penta-3,4-dien-2-yl)acetamide (**6c**). Off-white sold (107.2 mg, 99%). Eluent: EtOAc: hexane (1:9). mp 168–169 °C. ^1^H NMR (400 MHz, CDCl_3_): δ 7.45–7.28 (m, 14H), 6.80 (d, *J* = 2.8 Hz, 1H), 5.20 (dt, *J* = 8.9, 2.8 Hz, 1H), 5.07 (br, 1H), 2.47 (s, 1H); ^13^C{^1^H} NMR (100 MHz, CDCl_3_): δ 205.5, 161.4, 138.4, 134.1, 134.0, 132.7, 132.2, 129.1, 129.0, 128.7, 128.1, 127.9, 127.1, 127.0, 109.2, 101.5, 92.3, 72.0, 56.0. HRMS (ESI-TOF) *m*/*z* [M + H]^+^ Calcd for C_25_H_18_Cl_5_NO_2_Na: 561.9672, found 561.9650.

2,2,2-Trichloro-*N*-(3-(4-bromophenyl)-1-hydroxy-5-phenyl-1-(*p*-tolyl)penta-3,4-dien-2-yl)acetamide (**6h**). Off-white sold (112.0 mg, 99%). Eluent: EtOAc: hexane (1:9). mp 180–181 °C. ^1^H NMR (400 MHz, CDCl_3_): δ 7.52 (d, *J* = 8.5 Hz, 2H), 7.43–7.33 (m, 7H), 7.34–7.24 (m, 3H), 7.16 (d, *J* = 8.0 Hz, 2H), 6.76 (d, *J* = 2.5 Hz, 1H), 5.20 (dt, *J* = 8.9, 2.5 Hz, 1H), 5.06 (d, *J* = 1.8 Hz, 1H), 2.36 (s, 3H), 2.34 (br, 1H); ^13^C{^1^H} NMR (100 MHz, CDCl_3_): δ 205.5, 161.3, 138.0, 137.1, 133.1, 133.0, 132.0, 129.3, 128.9, 128.7, 128.3, 127.9, 127.1, 125.6, 121.9, 109.5, 101.4, 92.5, 72.4, 56.2, 21.1. HRMS (ESI-TOF) *m*/*z* [M + Na]^+^ Calcd for C_26_H_20_BrCl_3_NO_2_Na: 585.9713, found 585.9702.

### Crystallography

Crystals of **6c** and **6h** suitable for X-ray determination were obtained by recrystallization from diethyl ether/hexane solutions. Data were collected at room temperature on a Bruker D8 Venture diffractometer. The structure was solved using the SHELXS-97 program^13^ and refined using the SHELXL-97 program^14^ by full-matrix least-squares on F2 values.

## Data Availability

The data underlying this study are available in the published article and its online Supporting Information.

## Supporting Information Available

ORTEP plot of **6c**, crystal data of **6c** and **6h**, and spectra for all new compounds (^1^H NMR and ^13^C NMR) (PDF)
