# Supplementary material for: Preparation of 4-Allenyloxazolines from (Z)-2-En-4-yn-1-ol via Propargyl/Allenyl Isomerization
Source: J Org Chem. 2024 Aug 23;89(17):12762–8. doi: 10.1021/acs.joc.4c01152 (PMC11382262; doi:10.1021/acs.joc.4c01152)

Preparation of 4-Allenyloxazolines from (Z)-2-En-4-yn-1-ol *via* Propargyl/Allenyl Isomerization

Shi-Heng Hung, Yu-Min Wang,Yi-Hung Liu and Shiuh-Tzung Liu*

Department of Chemistry, National Taiwan University, Taipei 106, Taiwan

Supplementary Materials

Experimental procedure for preparation of **1** and spectal data....................S1
Figure S1. ORTEP plot of **6c** (30% probability ellipsoids).........................S6
Figure S2 ORTEP Plot of **6h** (30% probability ellipsoids) S7
Table S1 Crystal Data for **6c** and **6h**..S8
Spectra for all compounds...S8-S40
Figure S3. NOE signal enhancement (%) of **2a**..S41
Figure S4. Mass spectrum of compound **3**..S41

General Procedure for Preparation of Pent-2-en-4-yn-1-ols 1.[1]

To a solution of substituted pent-1-en-4-yn-3-ol (2 mmol) in THF (10 mL) was added an aqueous solution of H_2SO_4 (10% w/w, 5.88 g). The reaction was kept at ambient temperature and stirred overnight. Upon completion of reaction, saturated an aqueous solution of $NaHCO_3$ was added slowly to quench the reaction, and Et_2O (20 mL × 2) was added. The organic extracts were washed with water (15 mL) and brine (15 mL), dried with $MgSO_4$, and concentrated. The residue was chromatographed on silica gel with the use of ethyl acetate/hexane as the eluent.

Compound **1a**.[1] Colorless oil. (667.5 mg, 93%). Eluent: EtOAc: hexane (1: 10). ^{1}H NMR (400 MHz, $CDCl_3$): δ 7.68 (d, *J* = 8.7 Hz, 2H), 7.67-7.63 (m, 2H), 7.47-7.44 (m, 5H), 7.39 (d, *J* = 8.7 Hz, 2H), 7.35 (d, *J* = 7.5 Hz, 1H), 7.20 (d, *J* = 8.7 Hz, 1H), 6.67 (d, *J* = 8.7 Hz, 1H), 6.14 (d, *J* = 8.7 Hz, 1H), 3.17 (br, 1H), 2.54 (s, 3H). ^{13}C{H} NMR (100 MHz, $CDCl_3$): δ 142.5, 138.9(2C), 138.3, 135.6, 134.1, 131.7, 128.8, 128.6, 128.6, 128.5, 127.7, 126.7, 123.1, 122.9, 122.7, 97.0, 85.7, 73.1, 21.5. HRMS (ESI-TOF) *m/z* $[M+H]^+$ Calcd. for $C_{24}H_{20}ClO$: 359.1197, found 359.1198.

Compound **1b**. Colorless oil (624.2 mg, 86%). Eluent: EtOAc: hexane (1: 10). ^{1}H NMR (400 MHz, $CDCl_3$): δ 7.60 (d, *J* = 8.6 Hz, 2H), 7.57-7.54 (m, 2H), 7.53-7.49 (m, 2H), 7.41-7.39 (m, 3H), 7.33 (d, *J* = 8.6 Hz, 2H), 7.06 (t, *J* = 8.7 Hz, 2H), 6.53 (d, *J* = 8.6 Hz, 1H), 2.82 (s, 1H). ^{13}C{H} NMR (100 MHz, $CDCl_3$): δ 162.3 (d, $^1J_{C-F}$ = 246.0 Hz), 138.2, 138.1 (d, $^4J_{C-F}$ = 3.1 Hz), 135.3, 134.2, 131.6, 128.9, 128.6, 128.5, 128.5, 128.5, 127.6 (d, $^3J_{C-F}$ = 8.1 Hz), 127.5, 123.1, 122.4, 115.4 (d, $^2J_{C-F}$ = 21.5 Hz), 97.1, 85.2, 72.4. ^{19}F NMR (375 MHz, $CDCl_3$): δ -114.7. HRMS (ESI-TOF) *m/z* $[M+H]^+$ Calcd. for $C_{23}H_{17}ClFO$: 363.0946, found 363.0947.

Compound **1c**. Colorless oil. (704.8 mg, 93%). Eluent: EtOAc: hexane (1: 10). ^{1}H NMR (400 MHz, $CDCl_3$): δ 7.63 (d, *J* = 8.5 Hz, 2H), 7.59-7.56 (m, 2H), 7.50 (d, *J* = 8.3 Hz, 2H), 7.43-7.41 (m, 3H), 7.38-7.34 (m, 4H), 6.52 (d, *J* = 8.6 Hz, 1H), 6.07 (d, *J* = 8.6 Hz, 1H), 2.56 (br, 1H);^{13}C NMR (100 MHz, $CDCl_3$): δ 140.8, 137.9, 135.3, 134.3, 133.5, 131.6, 128.9, 128.7, 128.6, 128.5, 127.6, 127.2, 123.5, 122.4, 97.1, 85.2, 72.3. HRMS (ESI-TOF) *m/z* $[M+H]^+$ Calcd.

for $C_{23}H_{17}Cl_2O$: 379.0651, found 379.0650.

Compound **1d**. Colorless oil. (697.3 mg, 93%). Eluent: EtOAc: hexane (1: 10). ^{1}H NMR (400 MHz, $CDCl_3$): δ 7.63 (d, *J* = 8.5 Hz, 2H), 7.60-7.57 (m, 2H), 7.49 (d, *J* = 8.6 Hz, 2H), 7.43-7.41 (m, 3H), 7.35 (d, *J* = 8.5 Hz, 2H), 6.95 (d, *J* = 8.6 Hz, 2H), 6.61 (d, *J* = 8.6 Hz, 1H), 6.05 (d, *J* = 8.6 Hz, 1H), 3.82 (s, 3H), 2.71 (br, 1H); ^{13}C{H} NMR (100 MHz, $CDCl_3$): δ 159.2, 138.9, 135.6, 134.7, 134.0, 131.6, 128.8, 128.5, 128.5, 127.5, 127.2, 122.6, 122.6, 114.1, 96.9, 85.5, 72.7, 55.2. HRMS (ESI-TOF) *m/z* $[M+H]^+$ Calcd. for $C_{24}H_{20}ClO_2$: 375.1146, found 375.1148.

Compound **1e**. Colorless oil. (518.0 mg, 64%). Eluent: EtOAc: hexane (1: 10). ^{1}H NMR (400 MHz, $CDCl_3$): δ 7.63 (d, *J* = 8.7 Hz, 2H), 7.59-7.56 (m, 2H), 7.41-7.39 (m, 3H), 7.34 (d, *J* = 8.7 Hz, 2H), 6.75 (d, *J* = 2.2 Hz, 2H), 6.55 (d, *J* = 8.7 Hz, 2H), 6.43 (t, *J* = 2.2 Hz, 1H), 6.02 (d, *J* = 8.7 Hz, 1H), 3.80 (s, 6H); ^{13}C{H} NMR (100 MHz, $CDCl_3$): δ 161.1, 142.2, 138.2, 134.1, 133.3, 132.1, 129.8, 129.2, 128.8, 128.7, 127.7, 122.4, 105.3, 100.8, 89.8, 88.1, 73.0, 55.6. HRMS (ESI-TOF) *m/z* $[M+H]^+$ Calcd. for $C_{25}H_{22}ClO_3$: 405.1252, found 405.1245.

Compound **1f**. Colorless oil. (585.0 mg, 75%). Eluent: EtOAc: hexane (1: 10). ^{1}H NMR (400 MHz, $CDCl_3$): δ 8.23 (d, *J* = 8.7 Hz, 2H), 7.72 (d, *J* = 8.6 Hz, 2H), 7.62 (d, *J* = 8.6 Hz, 2H), 7.59-7.55 (m, 2H), 7.45-7.40 (m, 3H), 7.36 (d, *J* = 8.7 Hz, 2H), 6.47 (d, *J* = 8.7 Hz, 1H), 6.20 (d, *J* = 8.7 Hz, 1H), 2.75 (br, 1H); ^{13}C{H} NMR (100 MHz, $CDCl_3$): δ 149.4, 147.3, 136.7, 135.0, 134.6, 131.6, 129.1, 128.7, 128.6, 127.6, 126.5, 124.5, 123.8, 122.1, 97.6, 84.9, 72.1. HRMS (ESI-TOF) *m/z* $[M+H]^+$ Calcd. for $C_{23}H_{17}ClNO_3$: 390.0891, found 390.0880.

Compound **1g**.[1] Colorless oil. (637.0 mg, 93%). Eluent: EtOAc: hexane (1: 10). ^{1}H NMR (400 MHz, $CDCl_3$): δ 7.74-7.70 (m, 2H), 7.65-7.62 (m, 2H), 7.53 (d, *J* = 8.1 Hz, 2H), 7.47-7.44 (m, 3H), 7.27 (d, *J* = 7.9 Hz, 2H), 7.11 (t, *J* = 8.7 Hz, 2H), 6.62 (d, *J* = 8.7 Hz, 1H), 6.13 (d, *J* = 8.7 Hz, 1H), 2.95 (br, 1H), 2.43 (s, 3H). ^{13}C{H} NMR (100 MHz, $CDCl_3$): δ 162.7 (d, $^1J_{C-F}$ = 247.8 Hz), 139.7, 138.4, 137.5, 133.3, 131.6, 129.4, 128.8, 128.5, 128.0 (d, $^2J_{C-F}$ = 8.1 Hz), 125.9, 122.8, 115.3 (d, $^3J_{C-F}$ = 21.6 Hz), 96.7, 85.9, 73.0, 21.1. ^{19}F NMR (375 MHz, $CDCl_3$): δ -113.5.

HRMS (ESI-TOF) *m/z* $[M+H]^+$ Calcd. for $C_{24}H_{20}FO$: 343.1493, found 343.1500.

Compound **1h**.[1] Colorless oil. (867.4 mg, 93%). Eluent: EtOAc: hexane (1: 10). ^1H NMR (400 MHz, $CDCl_3$): δ 7.57-7.53 (m, 4H), 7.47 (d, *J* = 8.7 Hz, 2H), 7.43 (d, *J* = 8.0 Hz, 2H), 7.40-7.38 (m, 3H), 7.20 (d, *J* = 8.0 Hz, 2H), 6.58 (d, *J* = 8.7 Hz, 1H), 6.03 (d, *J* = 8.7 Hz, 1H), 2.36 (s, 3H), 2.33 (br, 1H). $^{13}C\{H\}$ NMR (100 MHz, $CDCl_3$): δ 139.8, 139.0, 137.9, 136.4, 131.9, 131.8, 129.7, 129.1, 128.8, 128.2, 126.1, 123.2, 122.9, 122.6, 97.1, 85.6, 73.3, 21.4. HRMS (ESI-TOF) *m/z* $[M+H]^+$ Calcd. for $C_{24}H_{20}BrO$: 403.0692, found 403.0693.

Compound **1i**. Colorless oil. (687.1 mg, 93%). Eluent: EtOAc: hexane (1: 10). ^1H NMR (400 MHz, $CDCl_3$): δ 8.23 (d, *J* = 9.0 Hz, 2H), 7.85 (d, *J* = 9.0 Hz, 2H), 7.60-7.57 (m, 2H), 7.47-7.41 (m, 5H), 7.23 (d, *J* = 7.9 Hz, 2H), 6.76 (d, *J* = 8.6 Hz, 1H), 6.07 (dd, *J* = 8.6, 2.0 Hz, 1H), 2.38 (s, 3H), 2.27 (d, *J* = 2.9 Hz, 1H); $^{13}C\{H\}$ NMR (100 MHz, $CDCl_3$): δ 147.3, 143.4, 141.7, 139.1, 137.9, 131.6, 129.4, 129.0, 128.5, 127.0, 125.8, 123.6, 122.2, 122.1, 97.6, 84.6, 73.0, 21.1. HRMS (ESI-TOF) *m/z* $[M+H]^+$ Calcd. for $C_{24}H_{20}NO_3$: 370.1438, found 370.1443.

Compound **1j**.[1] Colorless oil. (581.9 mg, 86%). Eluent: EtOAc: hexane (1: 10). ^1H NMR (400 MHz, $CDCl_3$): δ 7.81 (d, *J* = 8.0 Hz, 2H), 7.77-7.74 (m, 2H), 7.68 (d, *J* = 8.0 Hz, 2H), 7.54-7.51 (m, 3H), 7.35 (d, *J* = 7.6 Hz, 2H), 7.34 (d, *J* = 7.6 Hz, 2H), 6.81 (d, *J* = 8.7 Hz, 1H), 6.30 (d, *J* = 8.7 Hz, 1H), 3.61 (br, 1H), 2.54 (s, 3H), 2.52 (s, 3H). $^{13}C\{H\}$ NMR (100 MHz, $CDCl_3$): δ 140.5, 138.5, 138.5, 137.6, 134.9, 132.1, 129.8, 129.6, 129.1, 128.9, 126.8, 126.5, 124.0, 123.6, 96.9, 86.9, 73.5, 21.6, 21.6. HRMS (ESI-TOF) *m/z* $[M+H]^+$ Calcd. for $C_{25}H_{23}O$: 339.1743, found 339.1747.

Compound **1k**.[1] Colorless oil. (535.3 mg, 76%). Eluent: EtOAc: hexane (1: 10). ^1H NMR (400 MHz, $CDCl_3$): δ 7.65 (d, *J* = 8.7 Hz, 2H), 7.59-7.56 (m, 2H), 7.46 (d, *J* = 7.9 Hz, 2H), 7.41-7.39 (m, 4H), 7.21 (d, *J* = 7.9 Hz, 2H), 6.91 (d, *J* = 8.7 Hz, 2H), 6.51 (d, *J* = 8.7 Hz, 1H), 6.51 (d, *J* = 8.7 Hz, 1H), 3.85 (s, 3H), 2.38 (s, 3H), 2.26 (br, 1H). $^{13}C\{H\}$ NMR (100 MHz, $CDCl_3$): δ 159.7, 139.9, 137.4, 136.6, 131.6, 129.7, 129.3, 128.6, 128.4, 127.5, 125.9, 123.5, 123.0, 113.8, 96.3, 86.1, 73.0, 55.3, 21.1. HRMS (ESI-TOF) *m/z* $[M+H]^+$ Calcd. for $C_{25}H_{23}O_2$:

355.1693, found 355.1688.

Compound **1l**. Colorless oil. (488.1 mg, 93%). Eluent: EtOAc: hexane (1: 10). ^{1}H NMR (400 MHz, $CDCl_3$): δ 7.57-7.54 (m, 2H), 7.51-7.48 (m, 1H), 7.45 (d, *J* = 8.0 Hz, 2H), 7.38-7.36 (m, 2H), 7.24 (d, *J* = 7.8 Hz, 2H), 5.99 (dq, *J* = 8.8, 1.4 Hz, 1H), 5.89 (d, *J* = 8.8 Hz, 1H), 2.66 (br, 1H), 2.41 (s, 3H), 2.04 (d, *J* = 1.4 Hz, 3H); ^{13}C{H} NMR (100 MHz, $CDCl_3$): δ 140.1, 139.3, 137.0, 131.4, 129.1, 128.2, 128.1, 125.7, 123.0, 119.3, 94.1, 87.8, 72.6, 23.0, 21.0. HRMS (ESI-TOF) *m/z* $[M+H]^+$ Calcd. for $C_{19}H_{19}O$: 263.1430, found 263.1428.

Compound **1m**. Colorless oil. (667.3 mg, 94%). Eluent: EtOAc: hexane (1: 10). ^{1}H NMR (400 MHz, $CDCl_3$): δ 7.56 (d, *J* = 8.8 Hz, 2H), 7.43 (d, *J* = 8.0 Hz, 2H), 7.33 (d, *J* = 8.8 Hz, 2H), 7.22 (d, *J* = 8.0 Hz, 2H), 6.57 (d, *J* = 8.6 Hz, 1H), 5.98 (d, *J* = 8.6 Hz, 1H), 2.43 (br, 1H), 2.39 (s, 3H), 0.34 (s, 9H); ^{13}C{H} NMR (100 MHz, $CDCl_3$): δ 139.8, 139.5, 137.5, 135.2, 134.0, 129.3, 128.4, 127.5, 125.8, 122.7, 102.8, 100.9, 72.8, 21.1, -0.2. HRMS (ESI-TOF) *m/z* $[M+H]^+$ Calcd. for $C_{21}H_{24}ClOSi$: 355.1729, found 355.1735.

Compound **1n**. Colorless oil. (630.0 mg, 93%). Eluent: EtOAc: hexane (1: 10). ^{1}H NMR (400 MHz, $CDCl_3$): δ 7.53 (d, *J* = 8.6 Hz, 2H), 7.38 (d, *J* = 8.0 Hz, 2H), 7.28 (d, *J* = 8.6 Hz, 2H), 7.18 (d, *J* = 8.0 Hz, 2H), 6.43 (d, *J* = 8.6 Hz, 1H), 5.91 (d, *J* = 8.6 Hz, 1H), 2.50 (t, *J* = 7.0 Hz, 2H), 2.35 (s, 3H), 2.30 (br, 1H), 1.64 (quin, 7.2 Hz, 2H), 1.51 (sext, *J* = 7.3 Hz, 2H), 0.97 (t, *J* = 7.3 Hz, 3H); ^{13}C{H} NMR (100 MHz, $CDCl_3$): δ 140.0, 137.7, 137.7, 136.4, 134.0, 129.5, 128.6, 127.8, 126.0, 123.7(2C), 98.7, 73.1, 31.0, 22.4, 21.4, 19.6, 13.9. HRMS (ESI-TOF) *m/z* $[M+H]^+$ Calcd. for $C_{22}H_{24}ClO$: 339.1510, found 339.1520.

Compound **1o**. Colorless oil. (604.1 mg, 81%). ^{1}H NMR (400 MHz, $CDCl_3$): δ7.59 (d, *J* = 8.7 Hz, 2H), 7.41 (t, *J* = 8.2 Hz, 4H), 7.30 (d, *J* = 8.7 Hz, 2H), 7.17 (d, *J* = 7.7 Hz, 4H), 6.52 (d, *J* = 8.6 Hz, 1H), 6.00 (d, *J* = 8.4 Hz, 1H), 2.38 (s, 3H), 2.33 (s, 3H), 2.28 (d, *J* = 2.2 Hz, 1H); ^{13}C{^{1}H} NMR (100 MHz, $CDCl_3$): δ139.6, 139.0, 138.4(2C), 137.6, 135,8, 134.0, 131.5, 129.2, 128.5, 127.6, 125.9, 123.1, 119.6, 97.1, 84.8, 72.9, 21.5, 21.1. HRMS (ESI-TOF) *m/z* $[M+H]^+$ Calcd. for $C_{25}H_{22}ClO$: 373.1359, found 373.1362.

Compound **1p**. Colorless oil. (480.6 mg, 71%). ^{1}H NMR (400 MHz, $CDCl_3$): δ7.56-7.49 (m, 4H), 7.39-7.32 (m, 4H), 7.30-7.22 (m, 5H), 6.24 (d, *J* = 8.7 Hz, 1H), 6.11 (d, *J* = 8.8 Hz, 1H), 2.73 (s, 1H), 2.52 (s, 3H), 2.42 (s, 3H); ^{13}C{^{1}H} NMR (100 MHz, $CDCl_3$): δ 142.7, 139.7, 138.6, 137.2, 135.7, 131.4, 130.3, 129.2, 128.8, 128.4, 128.3, 127.8, 125.8, 125.7, 124.3, 122.9, 96.3, 86.6, 72.5, 21.0, 20.2. HRMS (ESI-TOF) *m/z* $[M+H]^+$ Calcd. for $C_{25}H_{23}O$: 339.1749, found 339.1738.

References:

1. (a) Chiang, A.-C.; Liu, Y.-H.; Peng, S.-M.; Liu, S.-T. *Org. Lett.* **2022**, 24, 7649-7653. (b) Du, X.; Song, F.; Lu, Y.; Chen, H.; Liu, Y. *Tetrahedron* **2009**, *65*, 1839-1845. (c) Hsu, M.-T.; Liu, Y.-H.; Liu, S.-T. *Reactions* **2020**, *1*, 47-53.

Figure S1 ORTEP Plot of **6c** (30% probability ellipsoids)

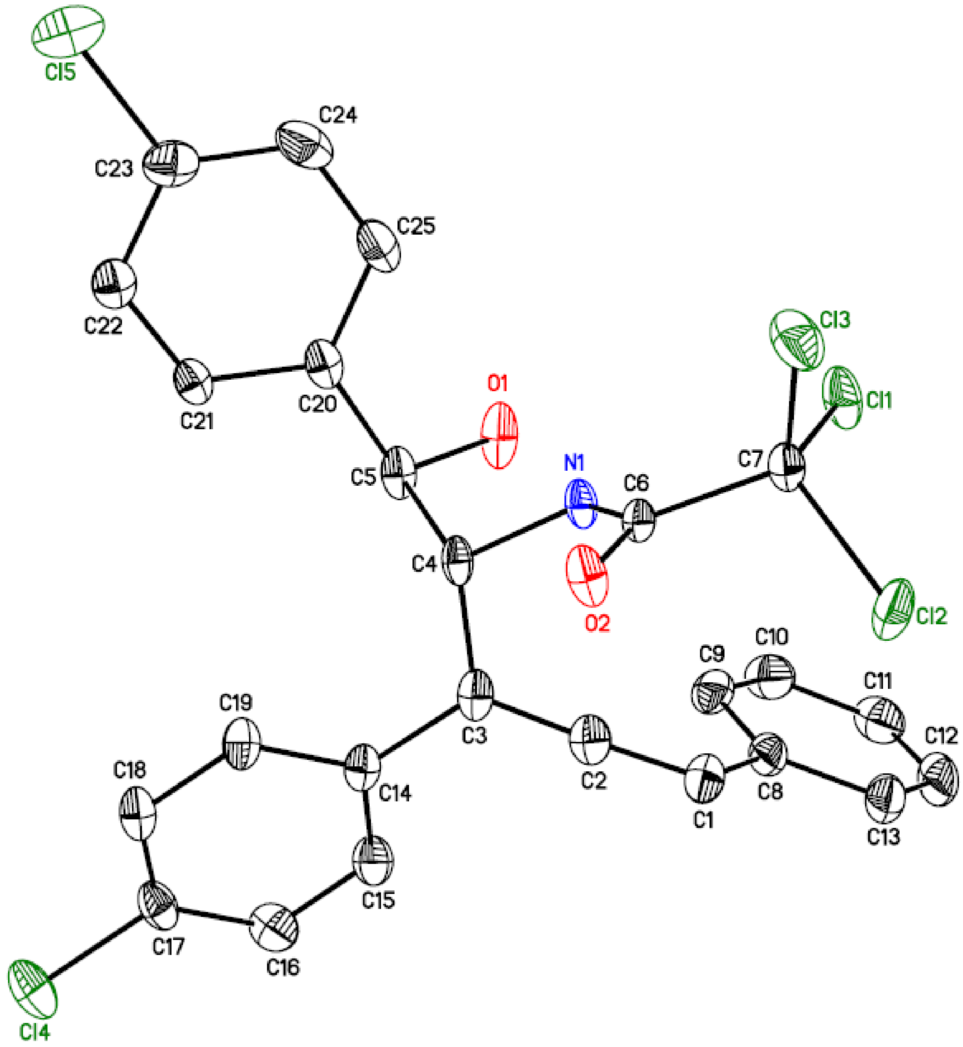

Figure S2 ORTEP Plot of **6h** (30% probability ellipsoids)

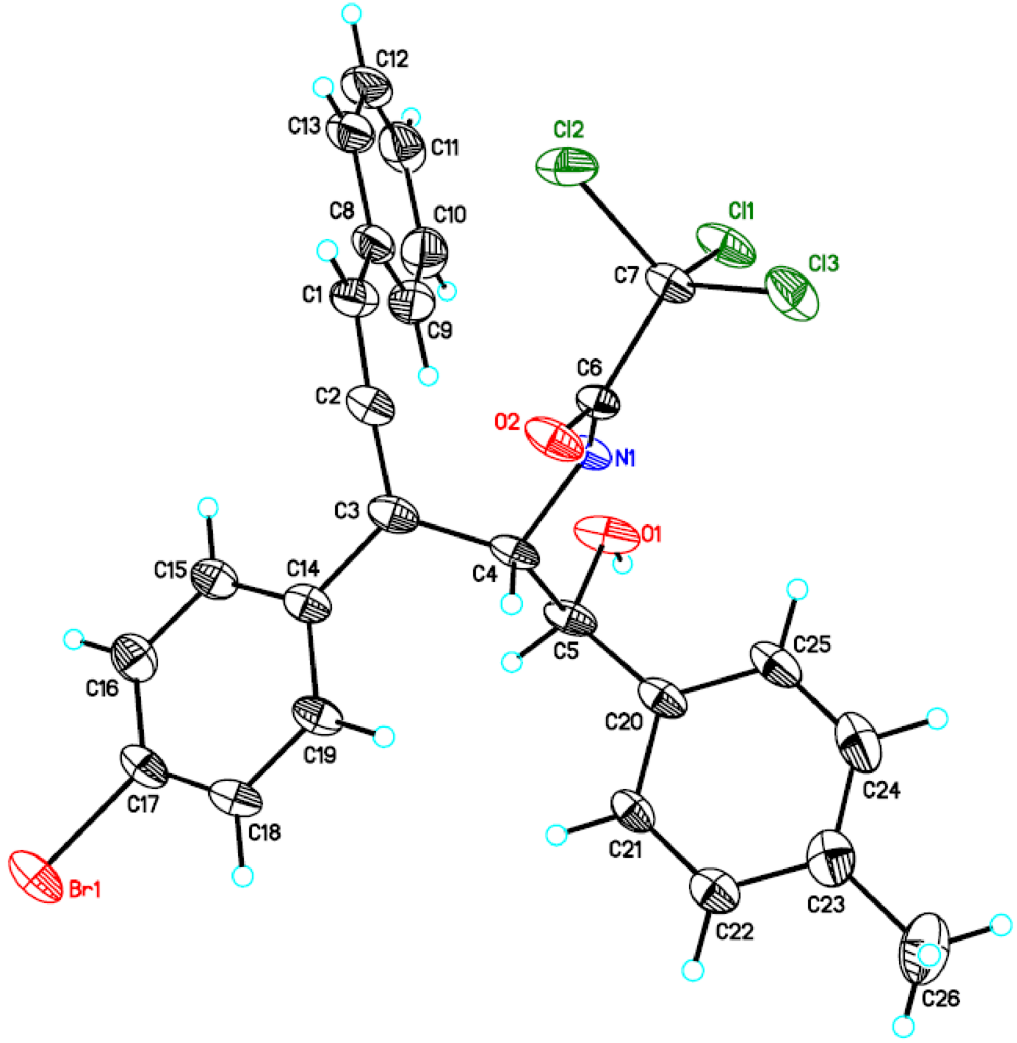

Table S1 Crystal Data for **6c** and **6h**

	6c	6h
Empirical formula	$C_{25}H_{18}Cl_5NO_2$	$C_{26}H_{20}BrCl_3NO_2$
Formula weight	541.65	564.69
Crystal system	Triclinic	Triclinic
Space group	P-1	P-1
Unit cell dimensions	a = 7.4539(4) Å	a = 7.4760(6) Å
	b = 12.3702(7) Å	b = 12.5395(10) Å
	c = 13.4550(7) Å	c = 13.5075(11) Å
	α= 90.3930(18)°	α= 91.031(3)°
	β= 93.5003(18)°	β= 93.475(3)°
	γ = 92.0612(17)°.	γ = 92.416(3)°
Volume	1237.47(12) Å ^{3}	1262.55(18) Å ^{3}
Z	2	2
F(000)	552	570
Density(Cacld.)	1.454 Mg/m^3	1.485 Mg/m^3
Wavelength	1.54178 Å	1.54178 Å
Theta range for Cell parameters	3.29 to 72.06°	3.278 to 72.439°
Absorption coefficient	5.534 mm^{-1}	5.331 mm^{-1}
Temperature	100(2) K	100(2) K
Crystal size	0.120 x 0.050 x 0.015 mm^3	0.100 x 0.050 x 0.050 mm^3
Max. and min. transmission	1.0000 and 0.7653	1.0000 and 0.7234
No. of measured reflections	21495	16609
No. of independent reflections	4753 [R(int) = 0.0477]	16609
Completeness to theta = 67.679°	98.7 %	98.0 %
Theta range for data collection	3.291 to 72.368°	3.278 to 72.439°
Final R indices [I>2sigma(I)]	R1 = 0.0429, wR2 = 0.1215	R1 = 0.0562, wR2 = 0.1617
R indices (all data)	R1 = 0.0442, wR2 = 0.1227	R1 = 0.0585, wR2 = 0.1643
Goodness-of-fit on F^2	1.050	1.071
Largest diff. peak and hole	0.829 and -0.578 e.Å $^{-3}$	1.760 and -0.766 e.Å $^{-3}$

^{1}H NMR (400 MHz, $CDCl_3$) Spectrum of **1a**

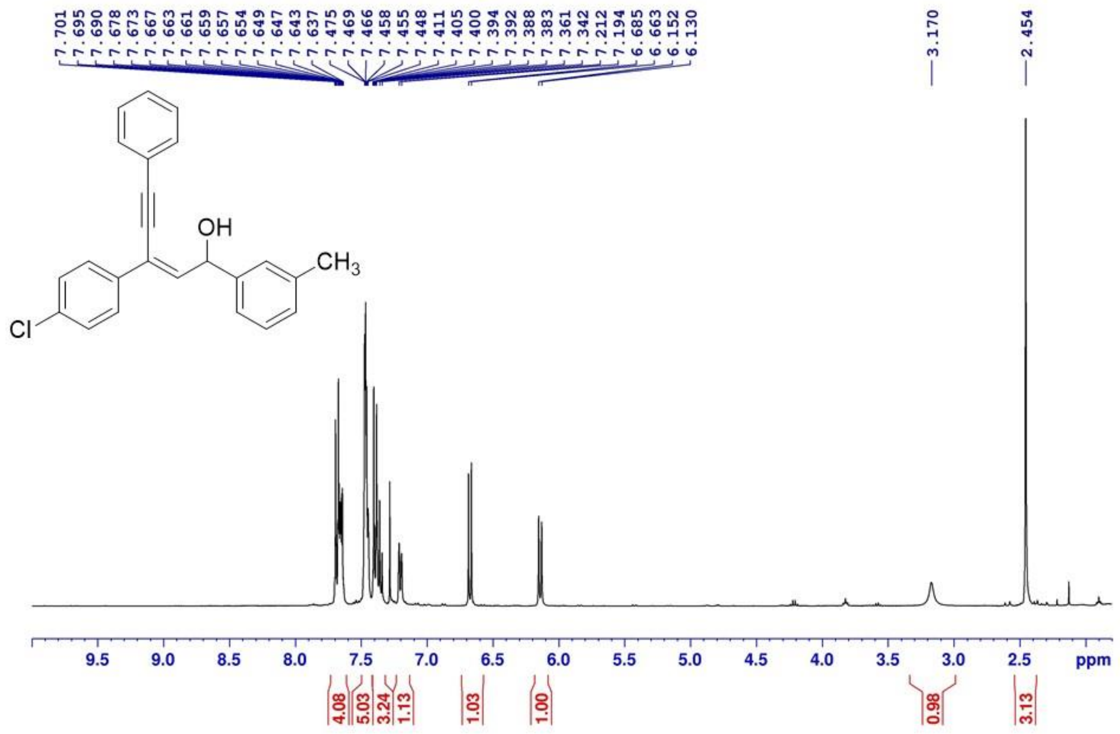

$^{13}C\{^{1}H\}$ NMR (100 MHz, $CDCl_3$) Spectrum of **1a**

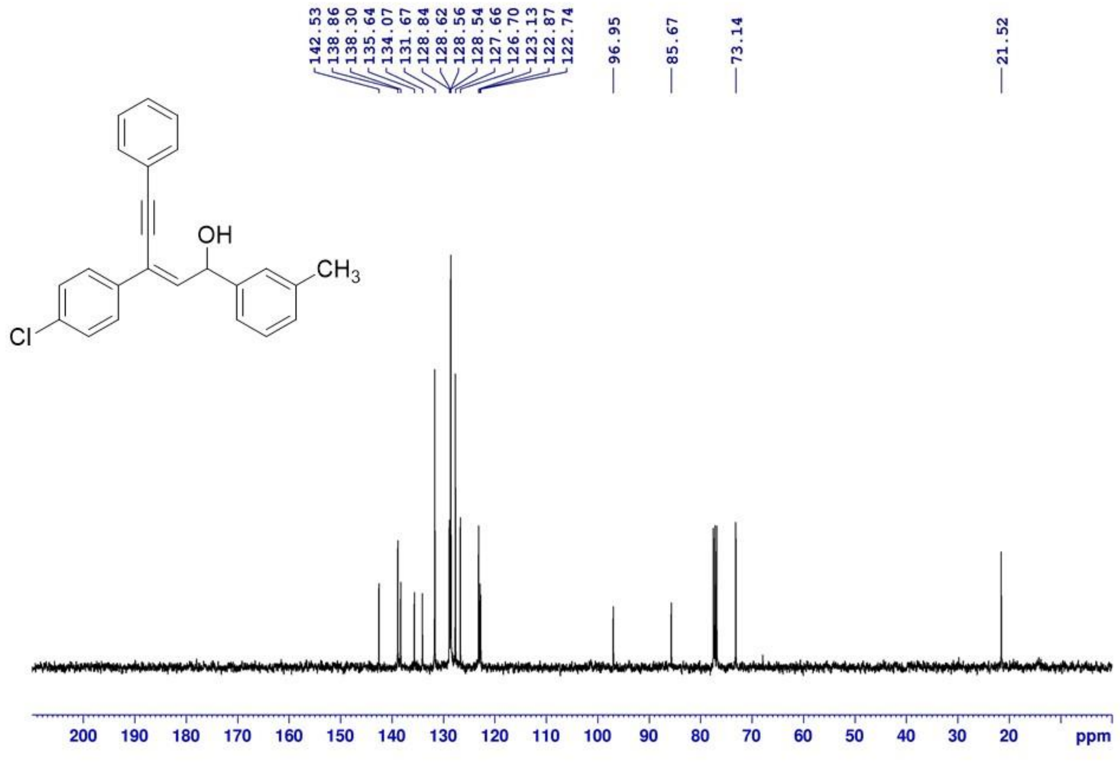

1H NMR (400 MHz, $CDCl_3$) Spectrum of **1b**

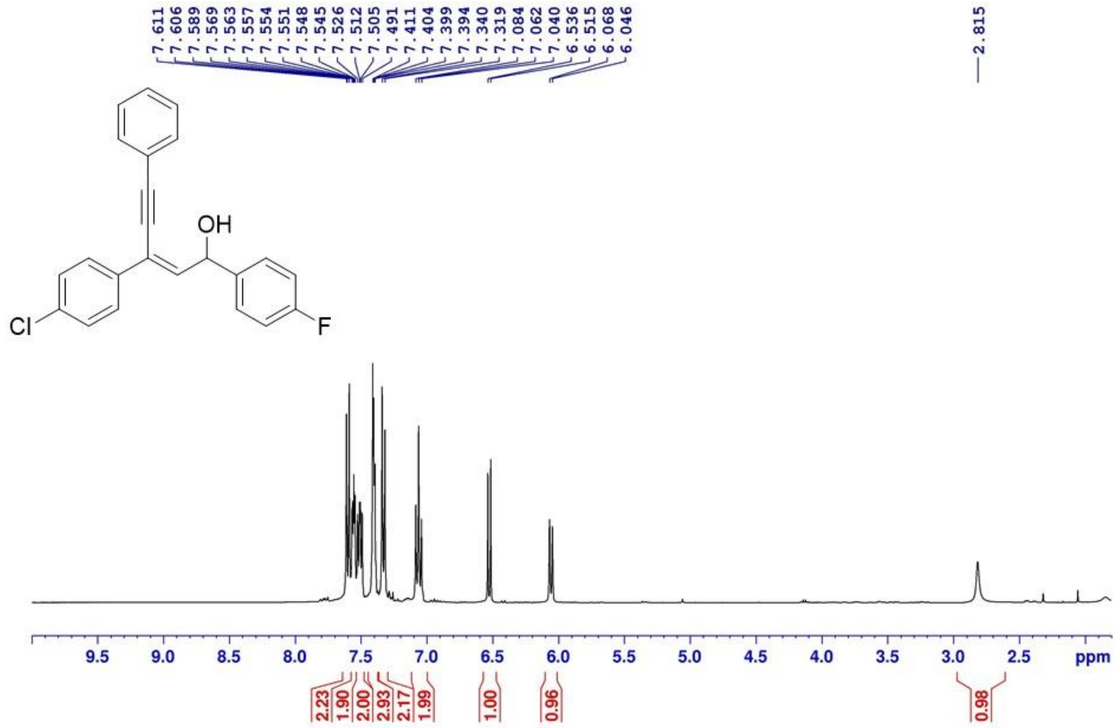

$^{13}C\{^1H\}$ NMR (100 MHz, $CDCl_3$) Spectrum of **1b**

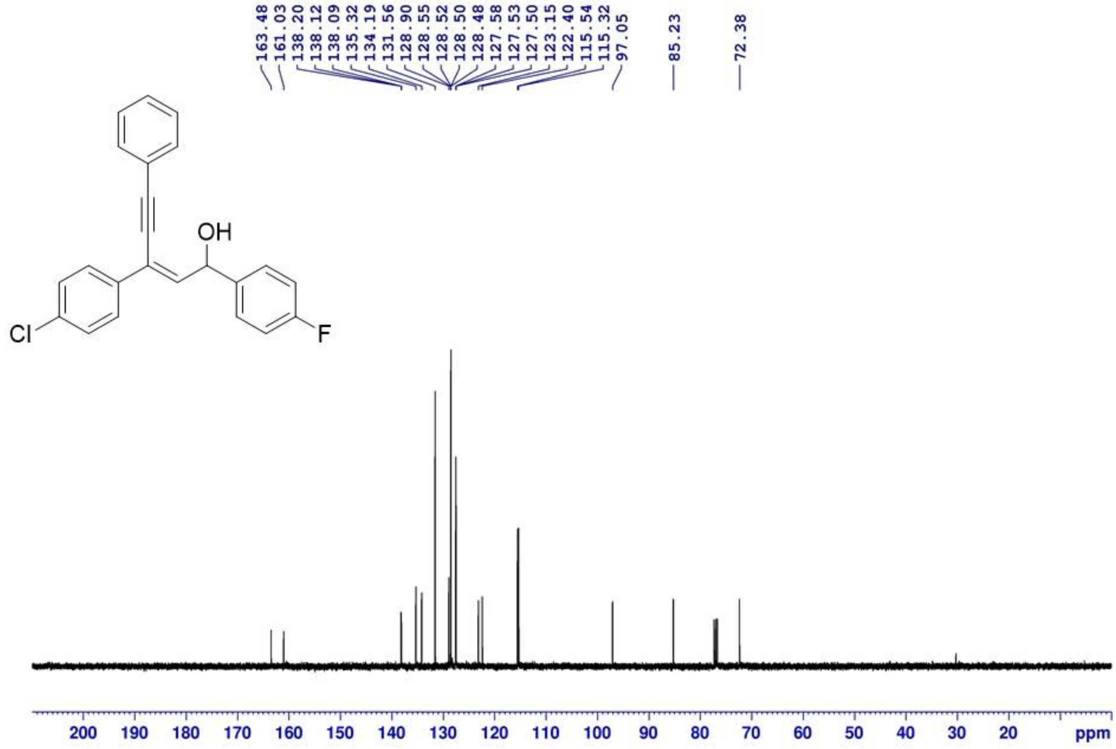

^{1}H NMR (400 MHz, $CDCl_3$) Spectrum of **1c**

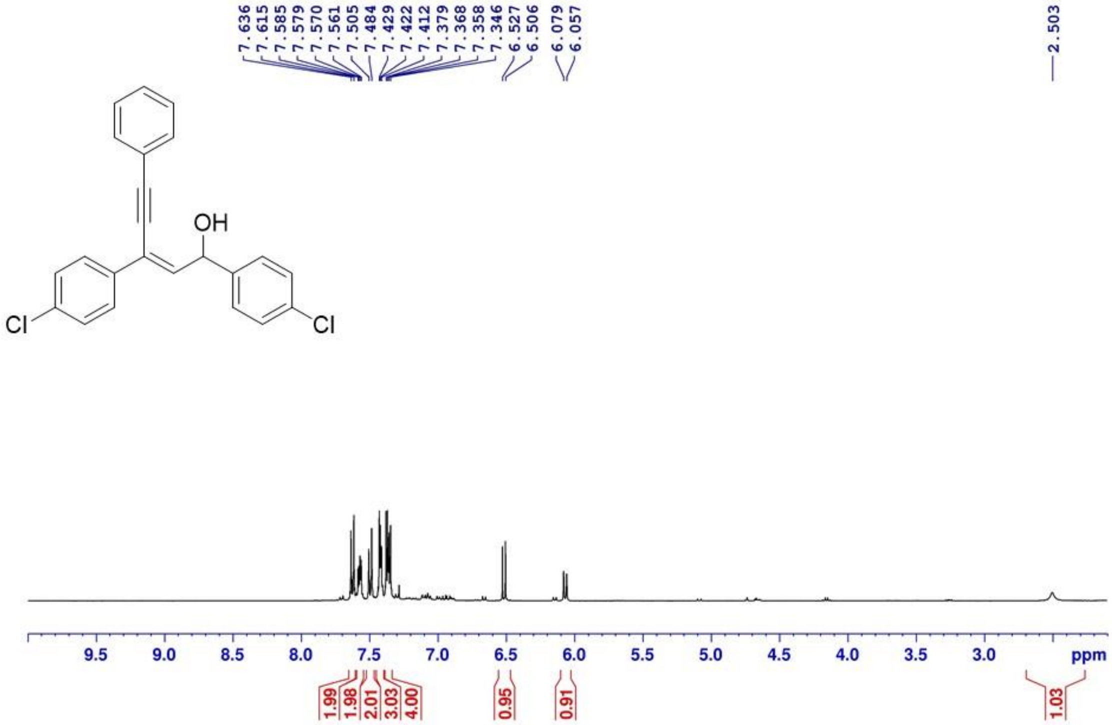

^{13}C{^{1}H} NMR (100 MHz, $CDCl_3$) Spectrum of **1c**

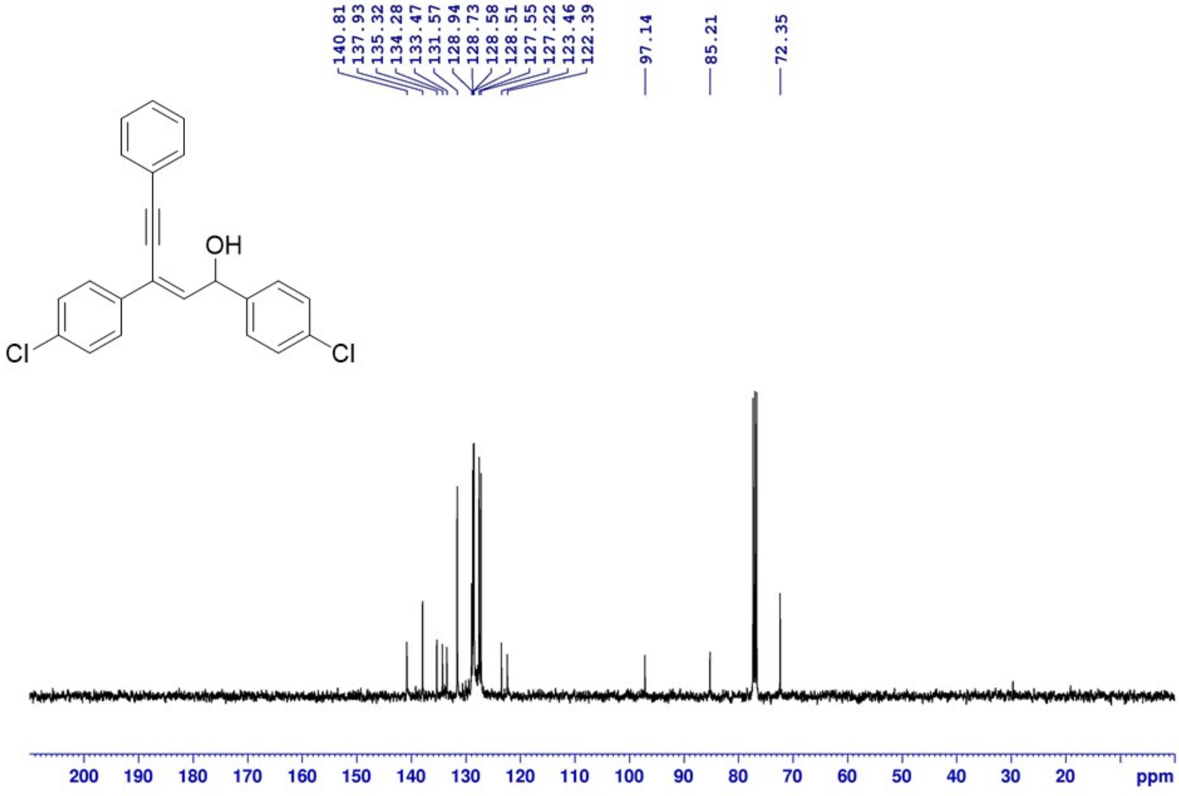

^{1}H NMR (400 MHz, $CDCl_3$) Spectrum of **1d**

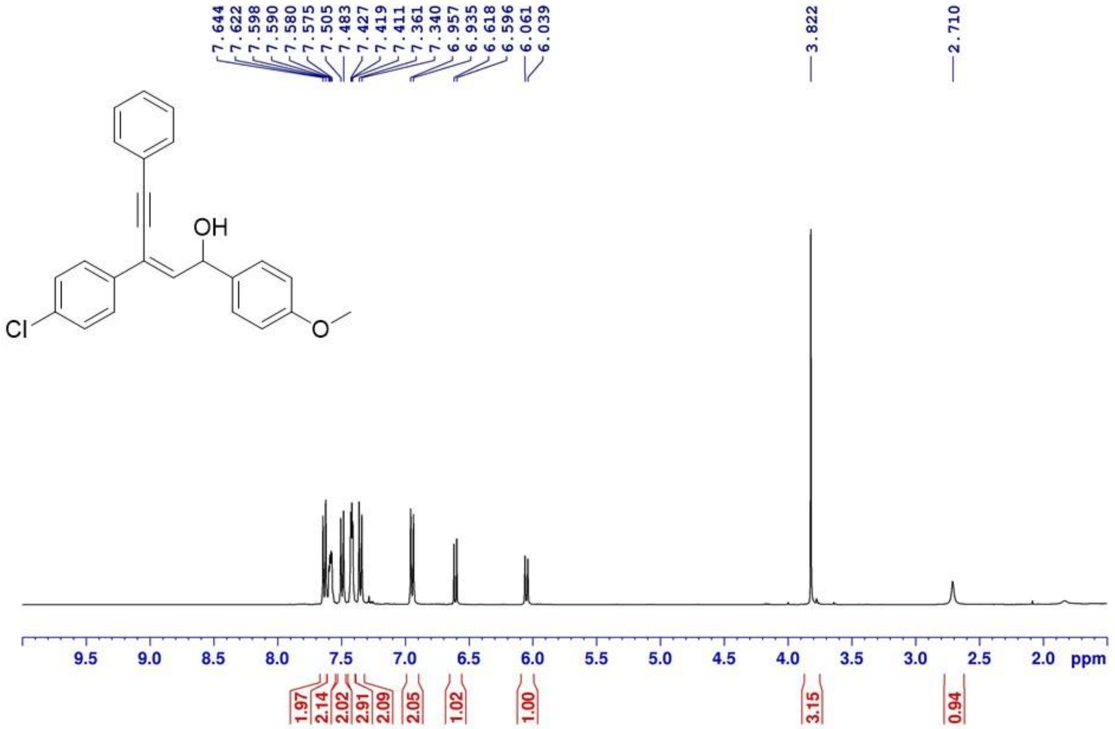

^{13}C{^{1}H} NMR (100 MHz, $CDCl_3$) Spectrum of **1d**

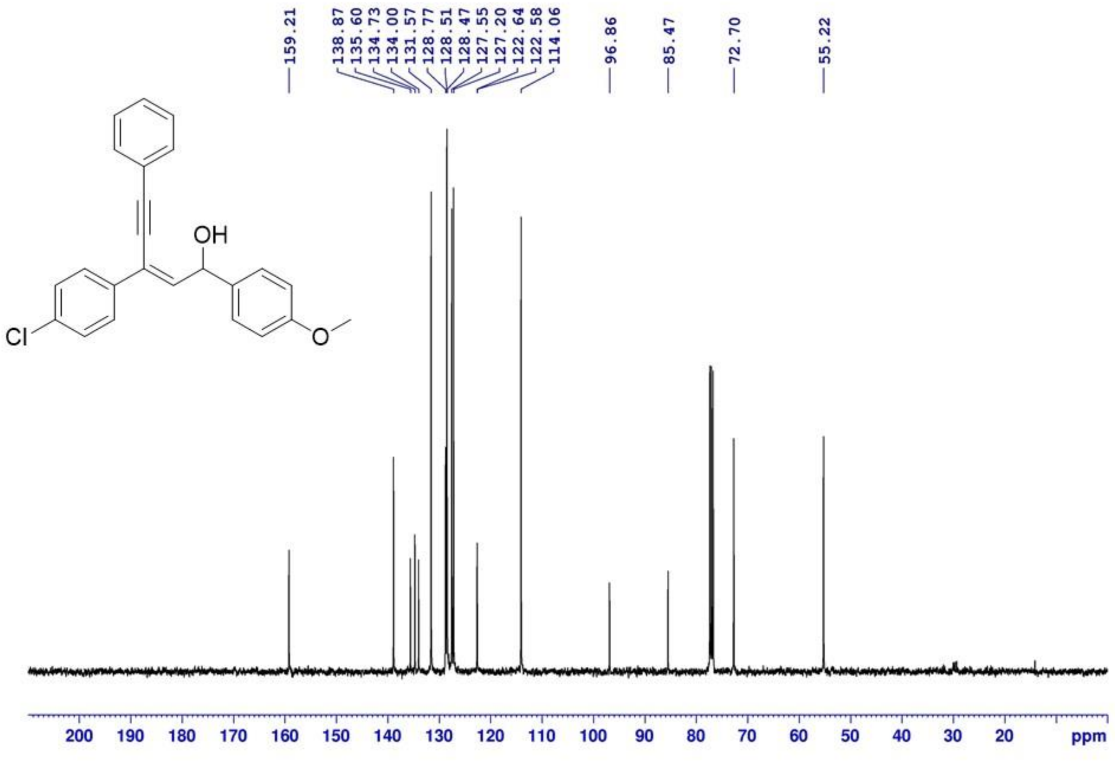

^{1}H NMR (400 MHz, $CDCl_3$) Spectrum of **1e**

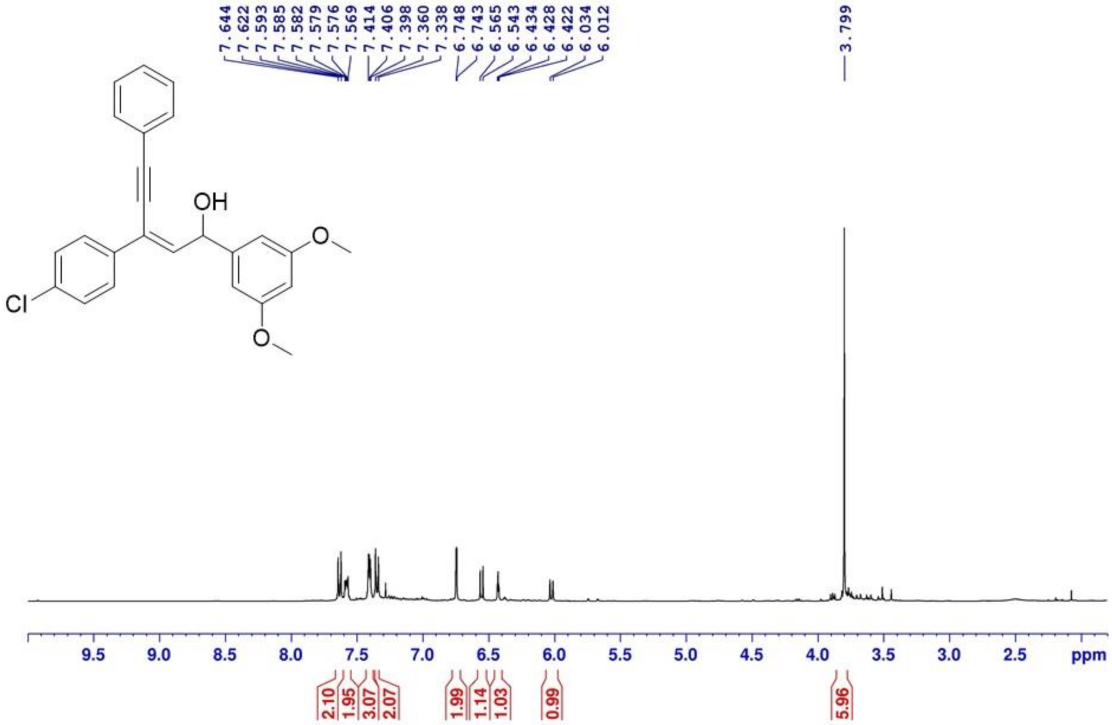

$^{13}C\{^{1}H\}$ NMR (100 MHz, $CDCl_3$) Spectrum of **1e**

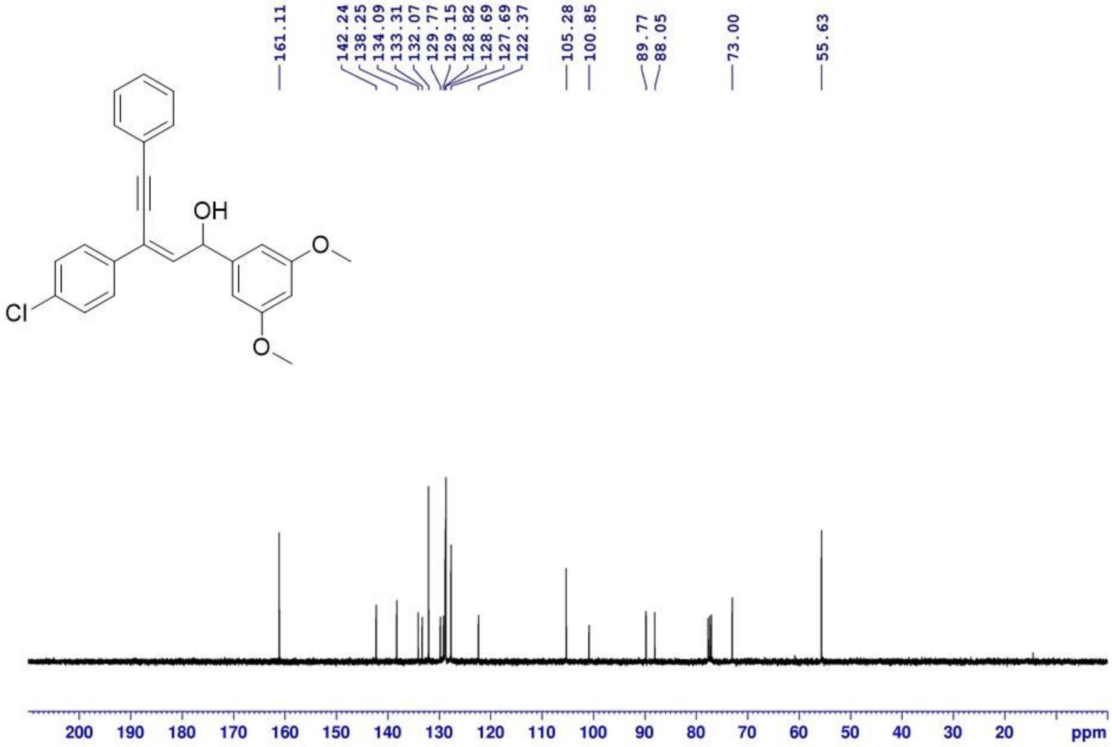

^{1}H NMR (400 MHz, $CDCl_3$) Spectrum of **1f**

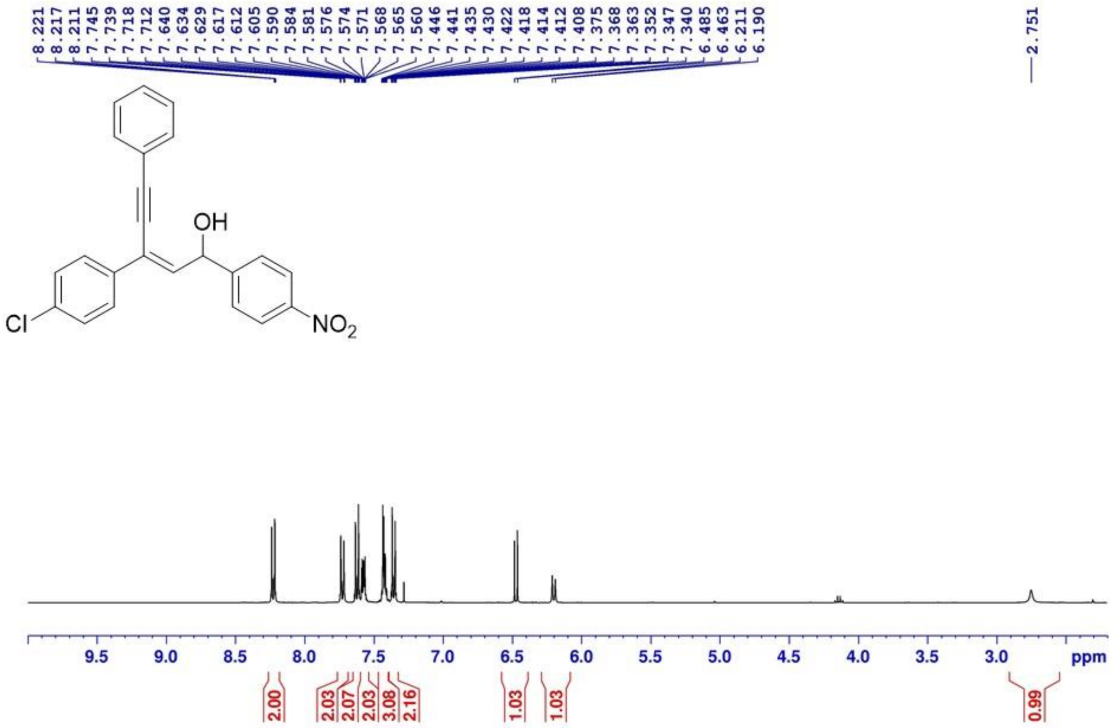

$^{13}C\{^1H\}$ NMR (100 MHz, $CDCl_3$) Spectrum of **1f**

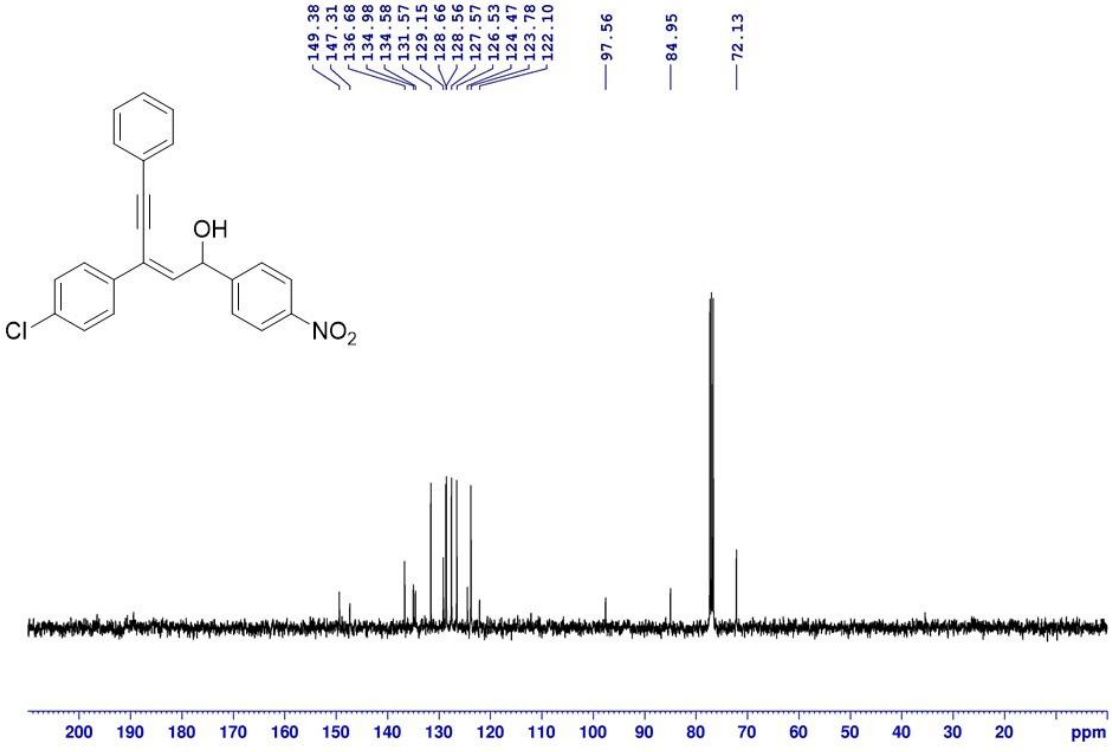

^{1}H NMR (400 MHz, $CDCl_3$) Spectrum of **1g**

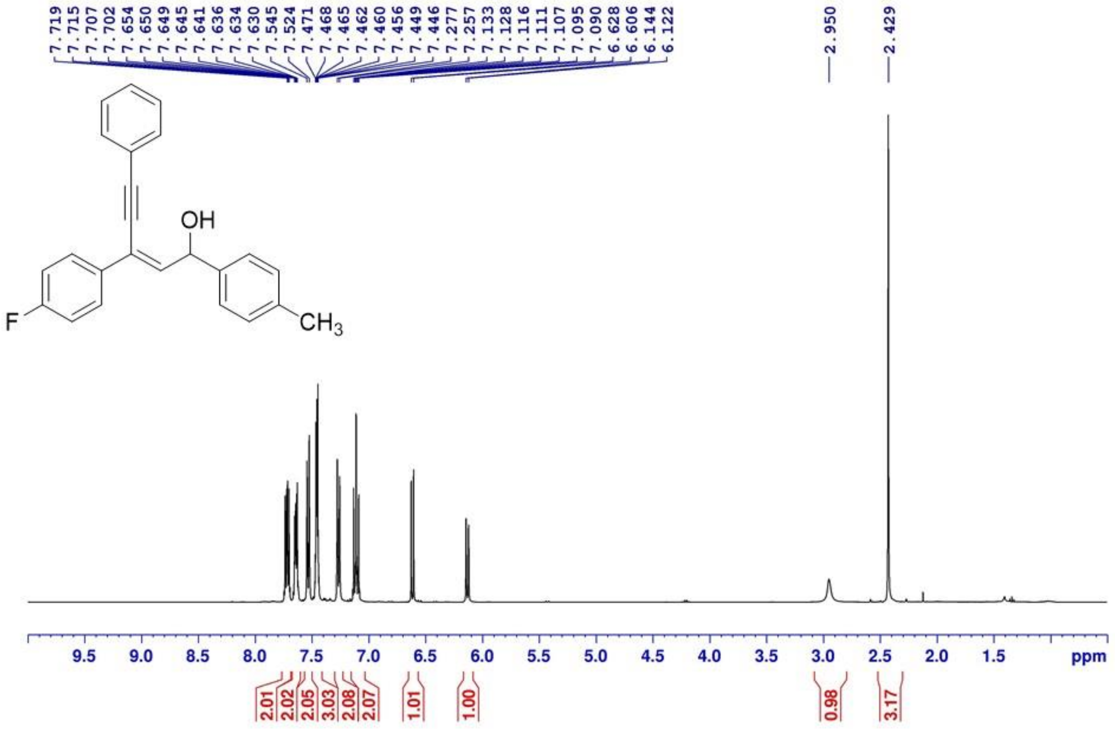

^{13}C{^{1}H} NMR (100 MHz, $CDCl_3$) Spectrum of **1g**

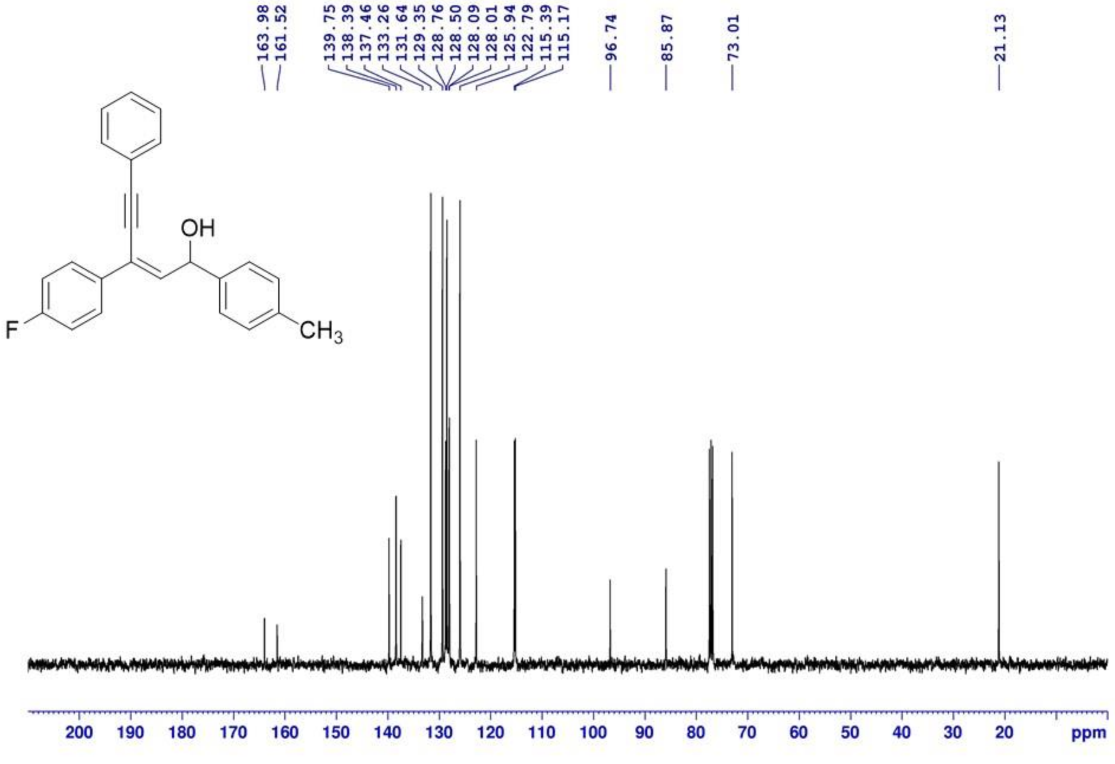

^{1}H NMR (400 MHz, $CDCl_3$) Spectrum of **1h**

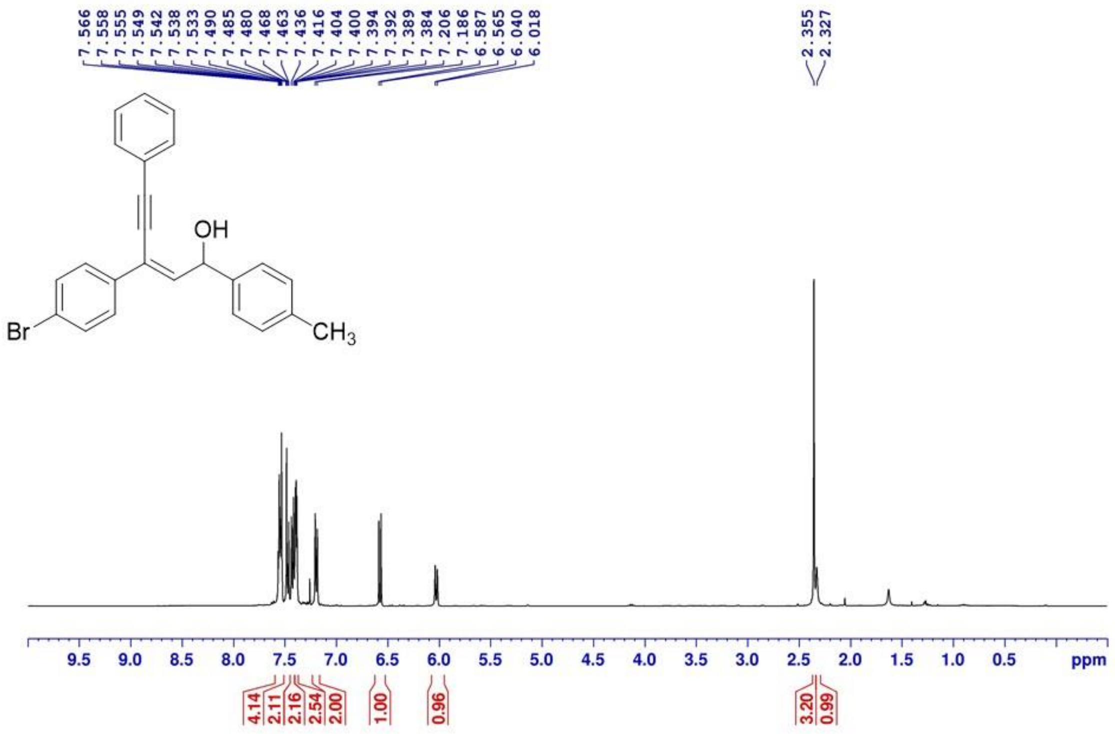

^{13}C{^{1}H} NMR (100 MHz, $CDCl_3$) Spectrum of **1h**

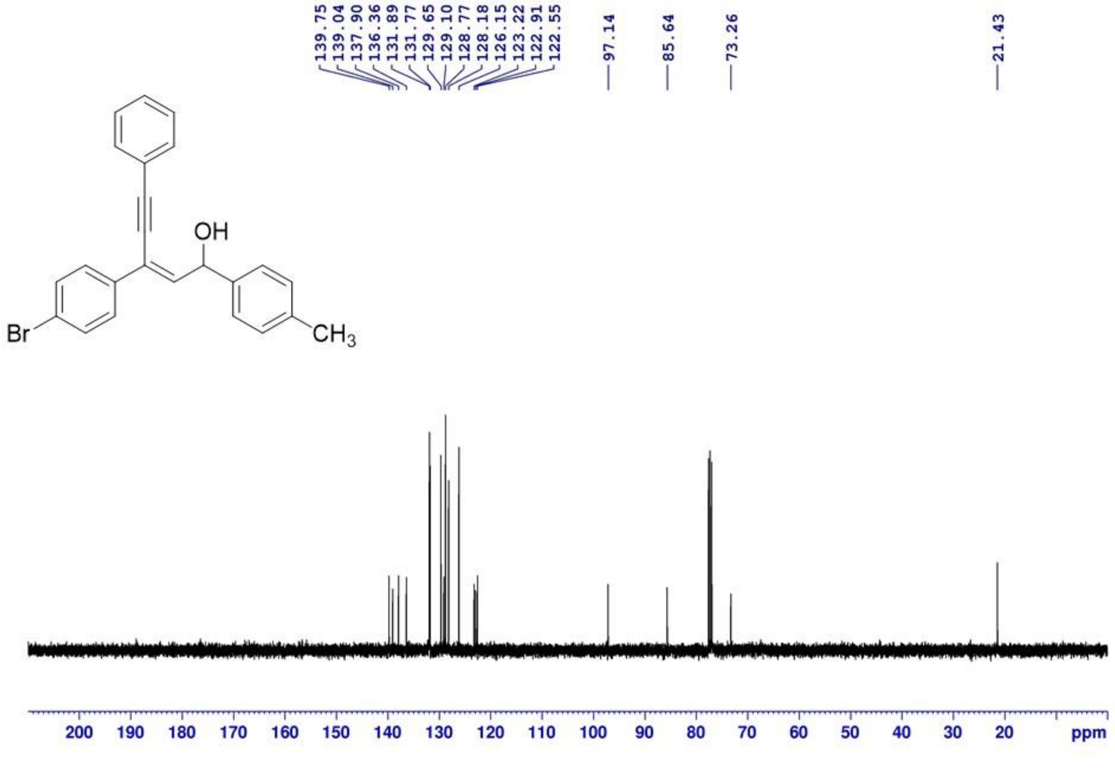

^{1}H NMR (400 MHz, $CDCl_3$) Spectrum of **1i**

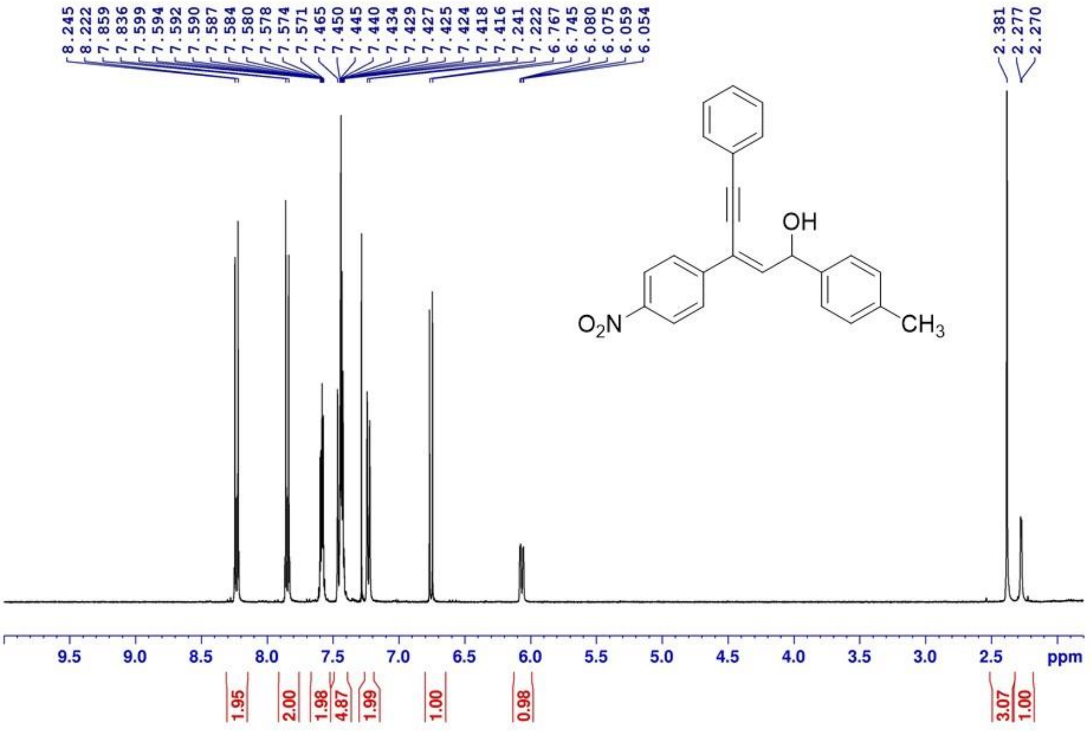

$^{13}C\{^{1}H\}$ NMR (100 MHz, $CDCl_3$) Spectrum of **1i**

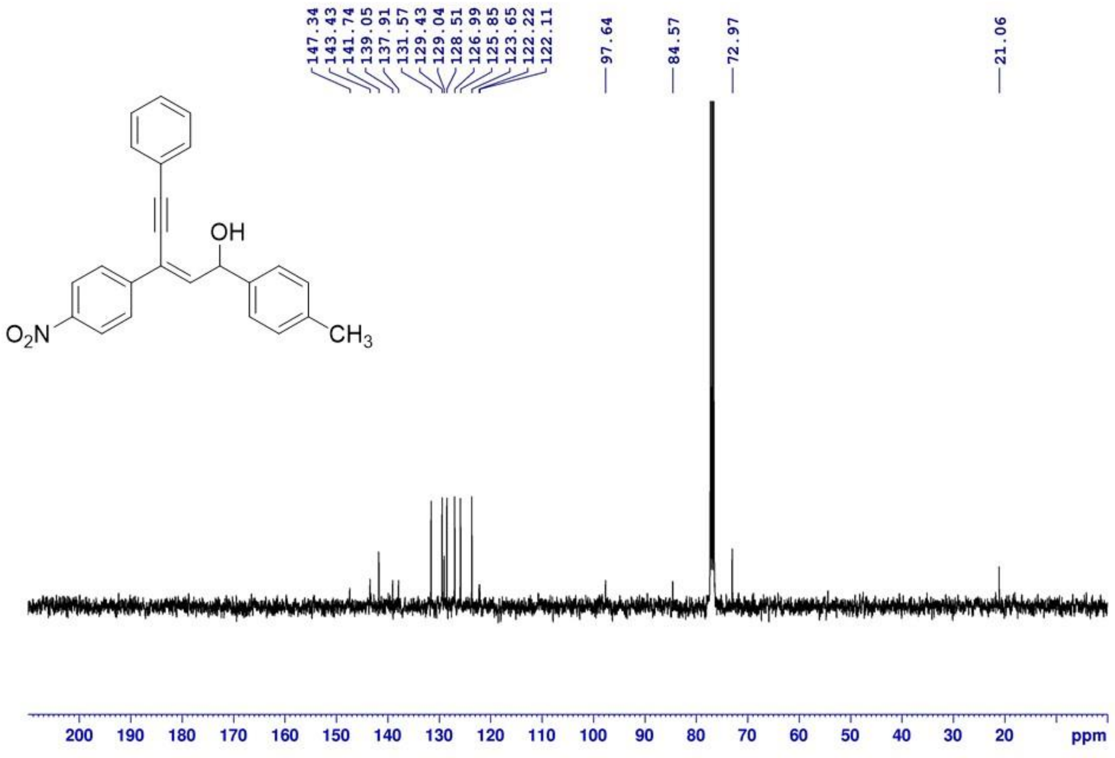

^{1}H NMR (400 MHz, $CDCl_3$) Spectrum of **1j**

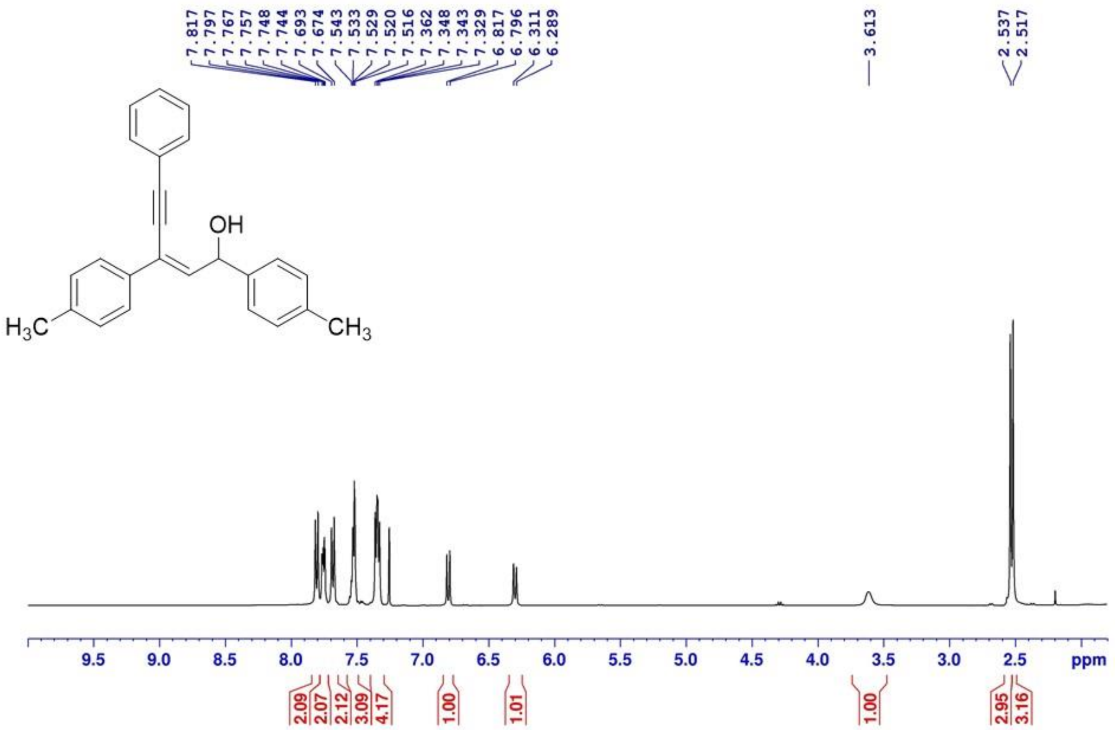

^{13}C{^{1}H} NMR (100 MHz, $CDCl_3$) Spectrum of **1j**

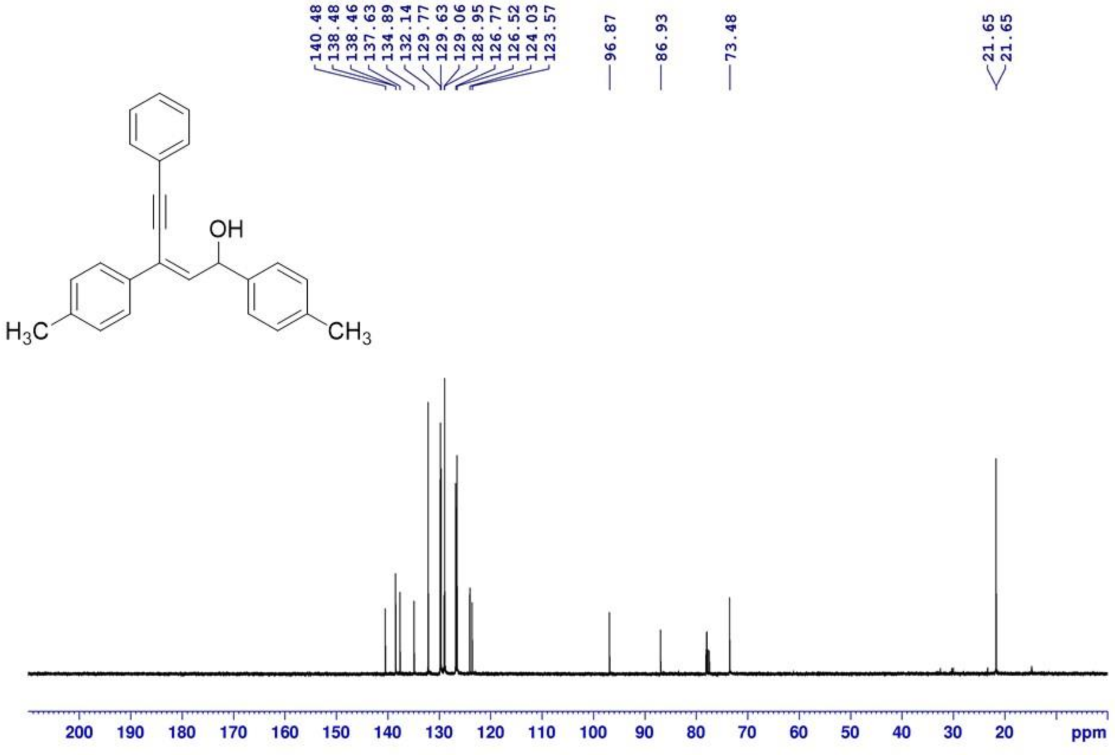

^{1}H NMR (400 MHz, $CDCl_3$) Spectrum of **1k**

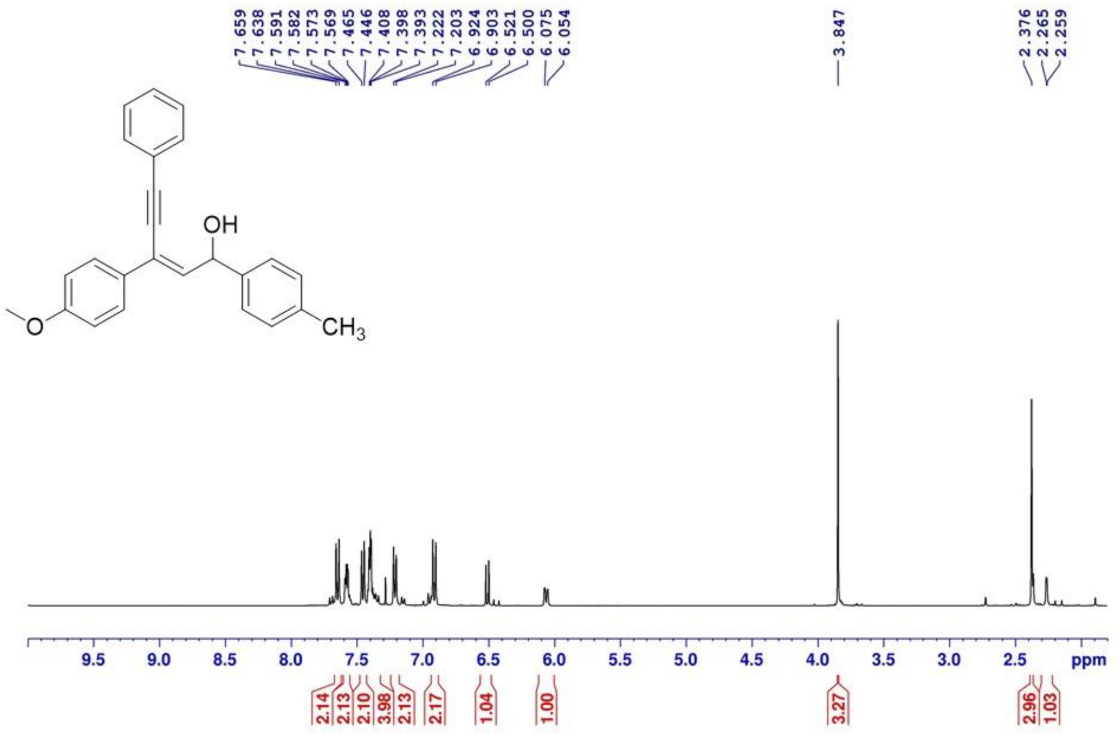

^{13}C{^{1}H} NMR (100 MHz, $CDCl_3$) Spectrum of **1k**

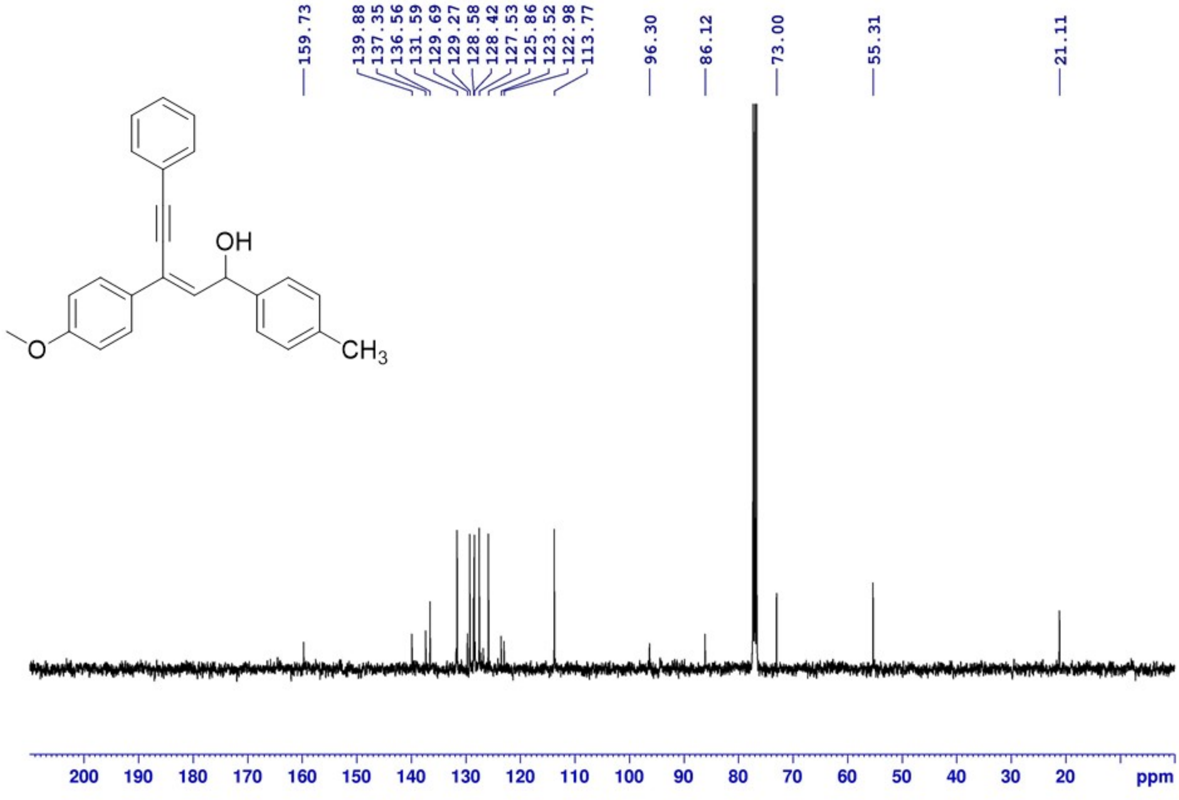

^{1}H NMR (400 MHz, $CDCl_3$) Spectrum of **1l**

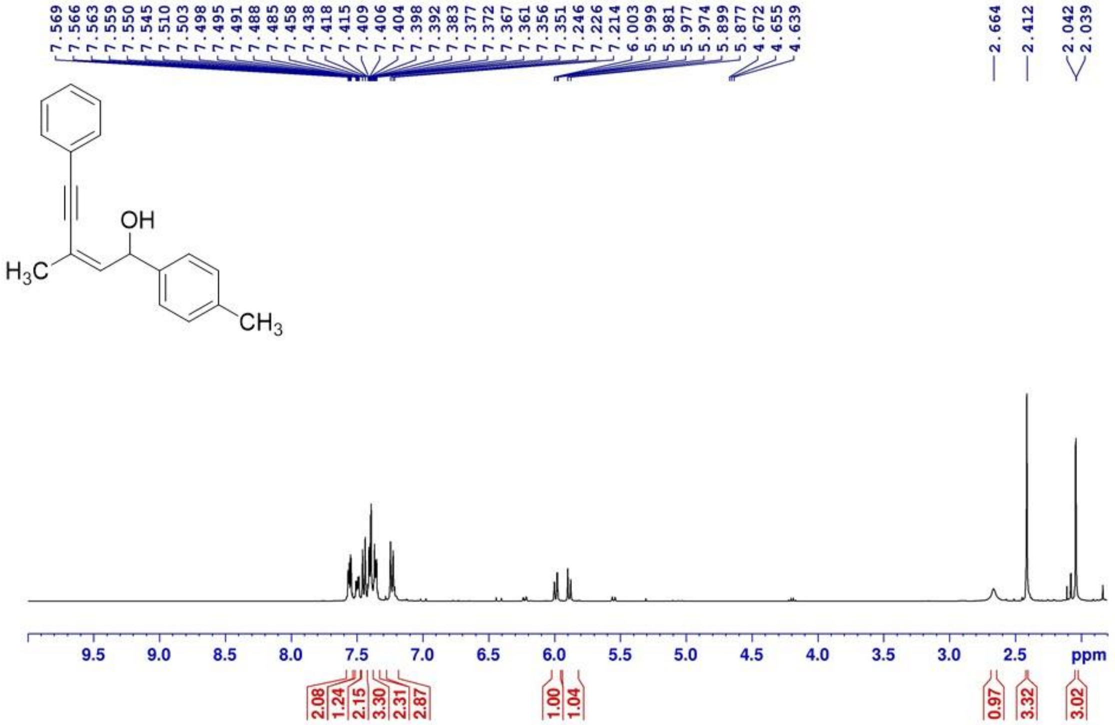

$^{13}C\{^{1}H\}$ NMR (100 MHz, $CDCl_3$) Spectrum of **1l**

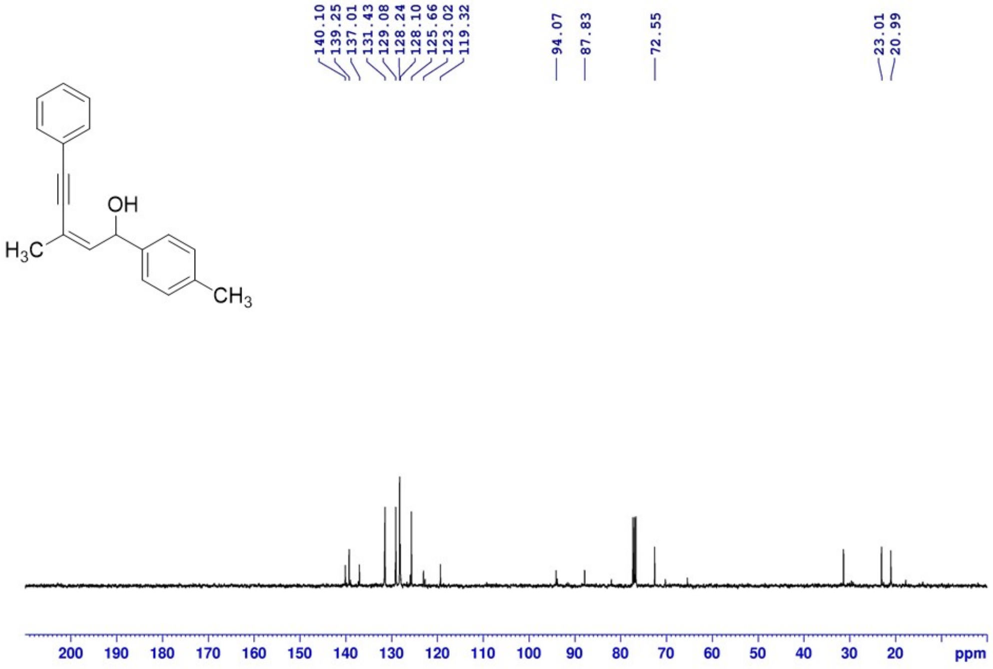

^{1}H NMR (400 MHz, $CDCl_3$) Spectrum of **1m**

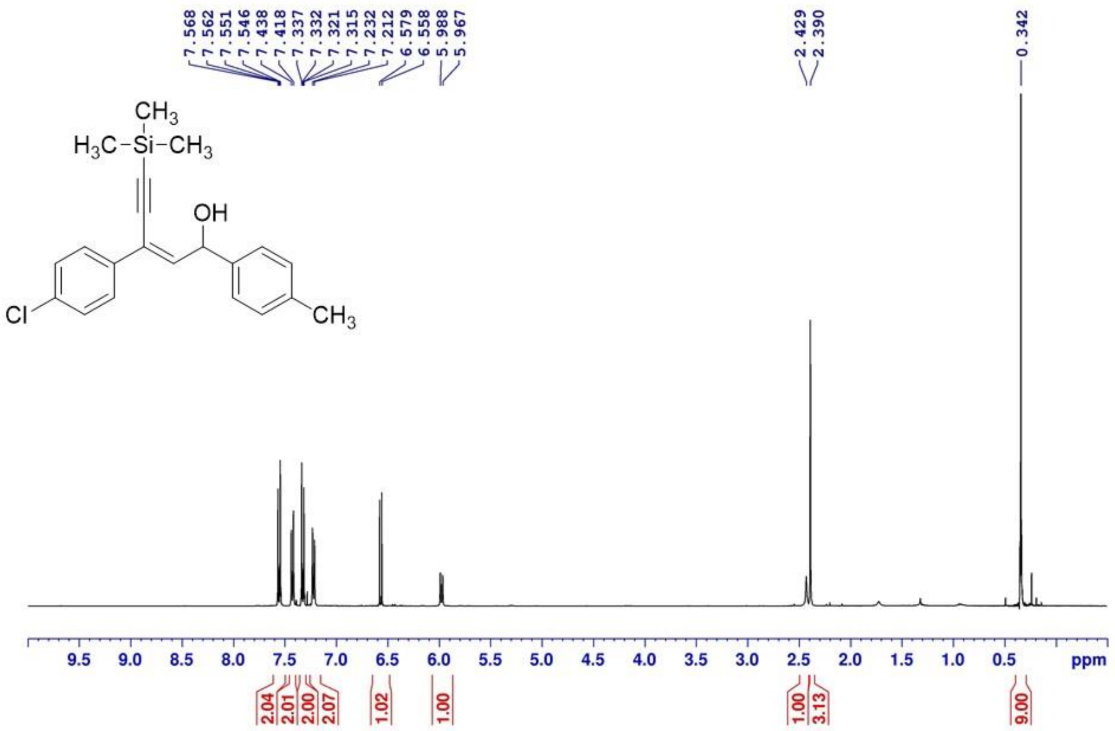

$^{13}C\{^1H\}$ NMR (100 MHz, $CDCl_3$) Spectrum of **1m**

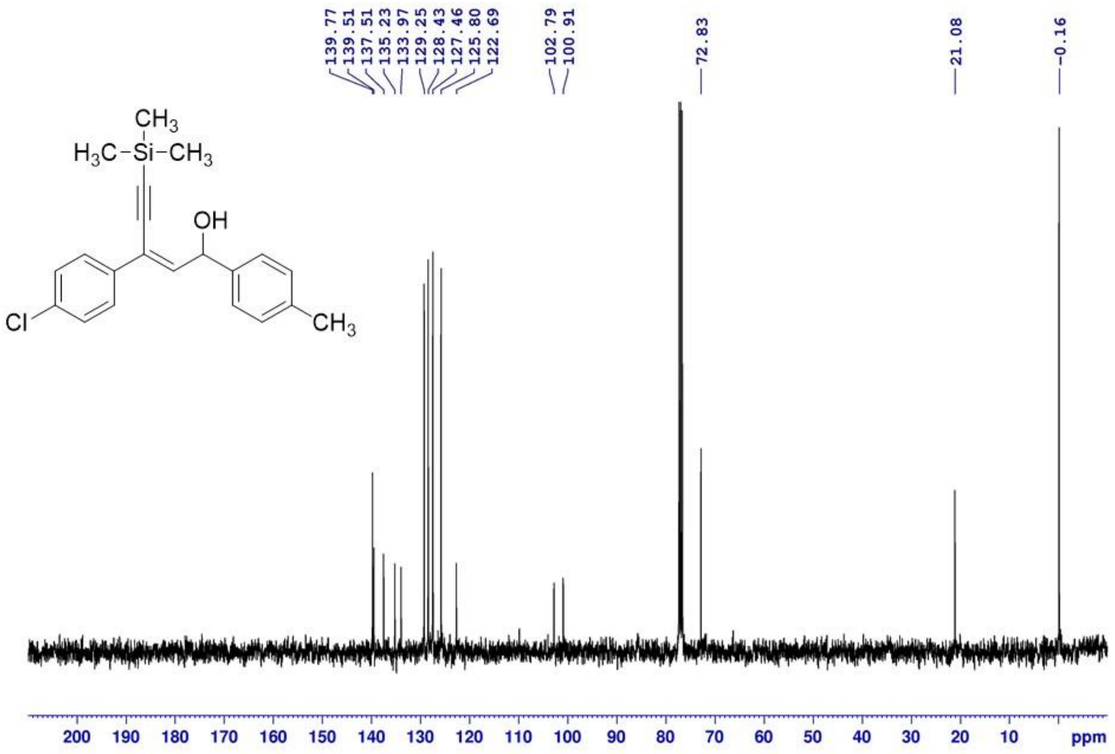

^{1}H NMR (400 MHz, $CDCl_3$) Spectrum of **1n**

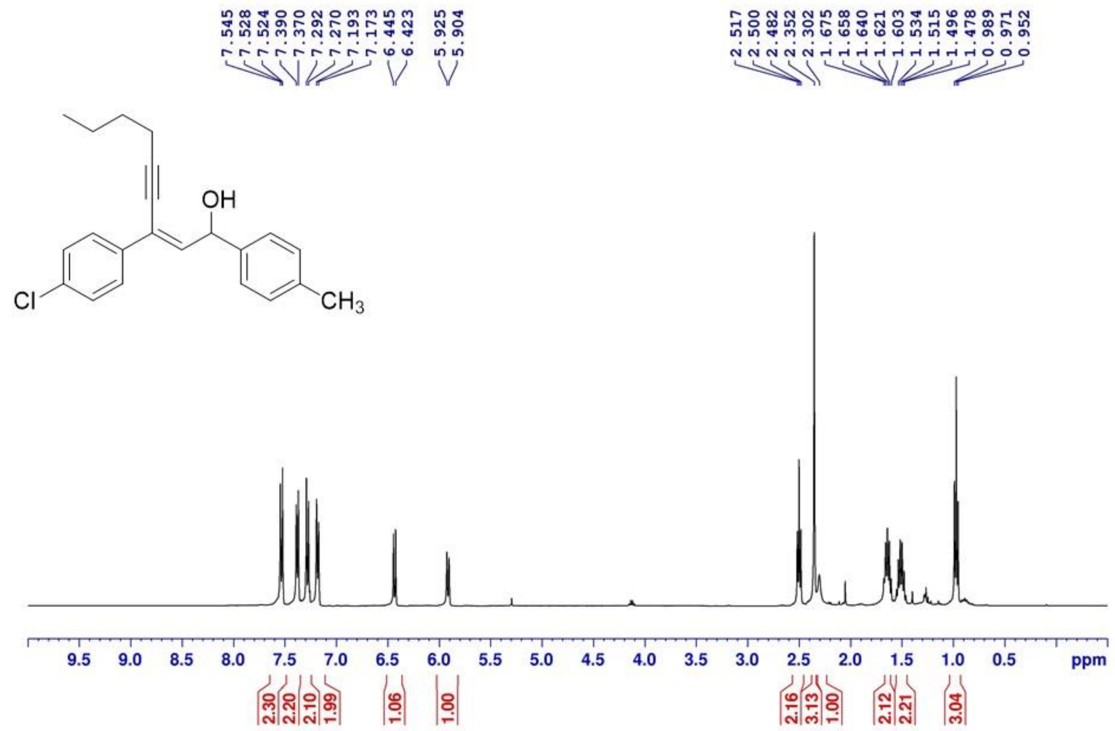

$^{13}C\{^{1}H\}$ NMR (100 MHz, $CDCl_3$) Spectrum of **1n**

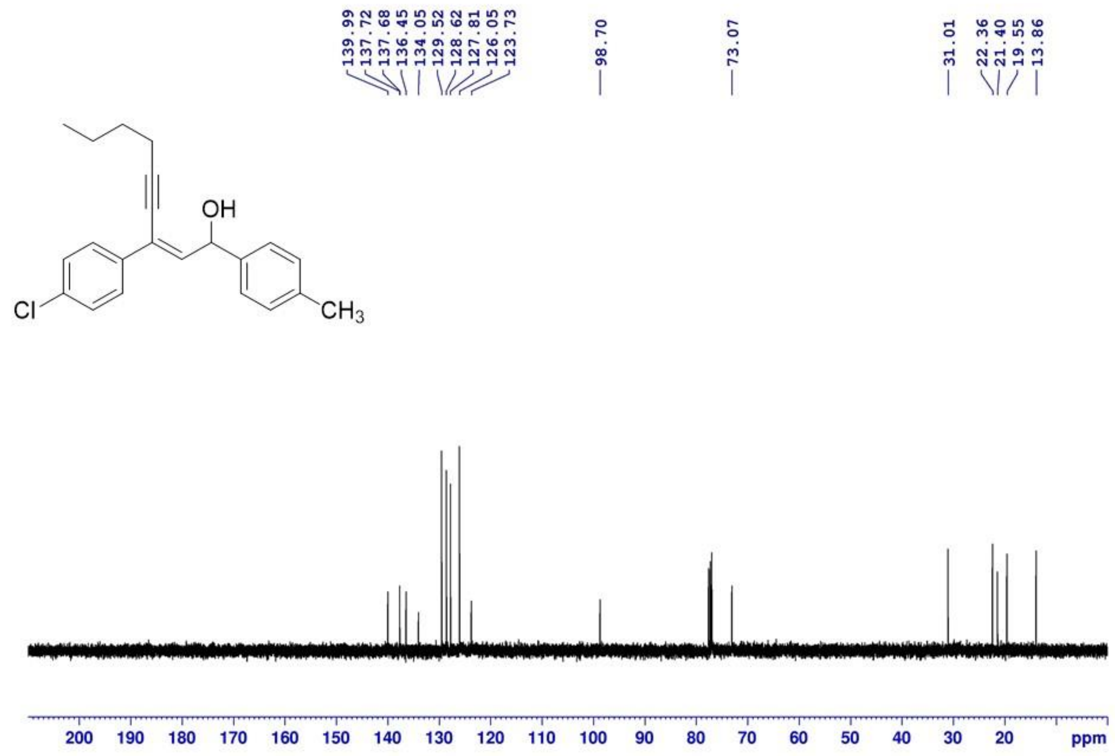

^{1}H NMR (400 MHz, $CDCl_3$) Spectrum of **1o**

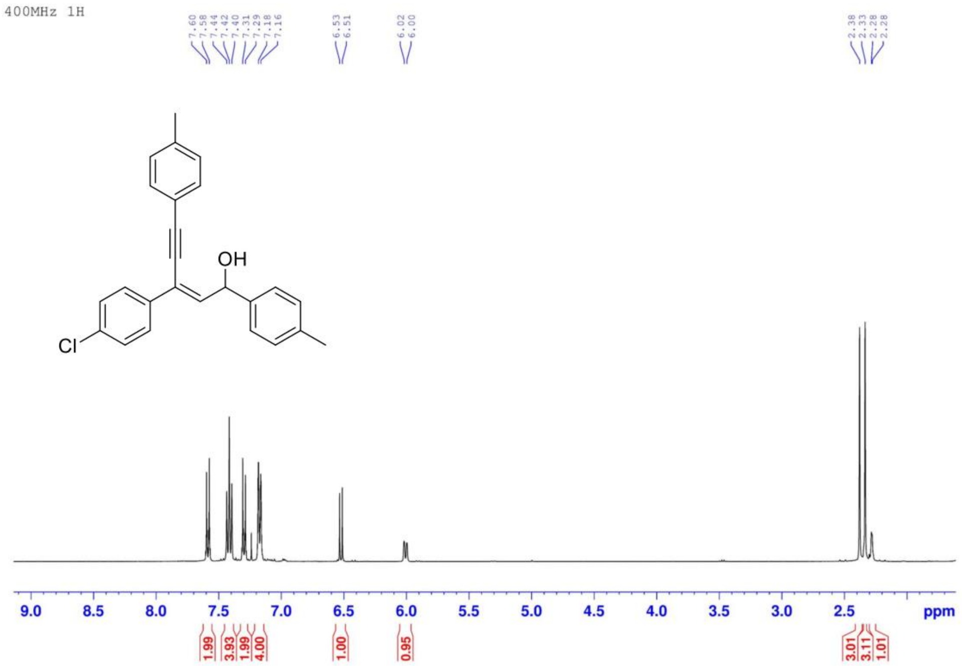

^{13}C{^{1}H} NMR (100 MHz, $CDCl_3$) Spectrum of **1o**

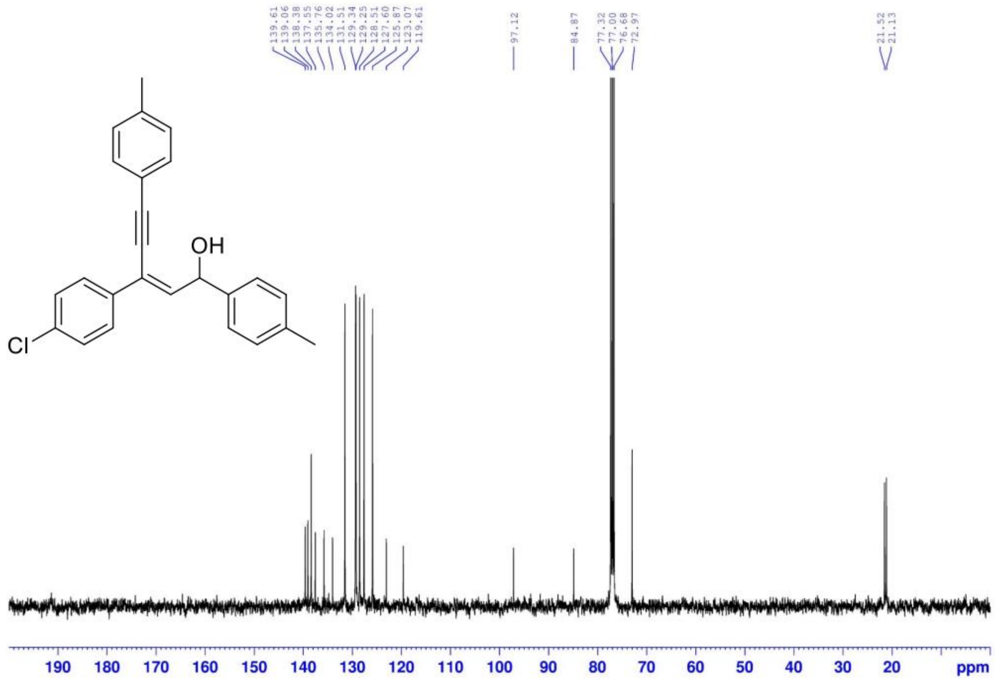

^{1}H NMR (400 MHz, $CDCl_3$) Spectrum of **1p**

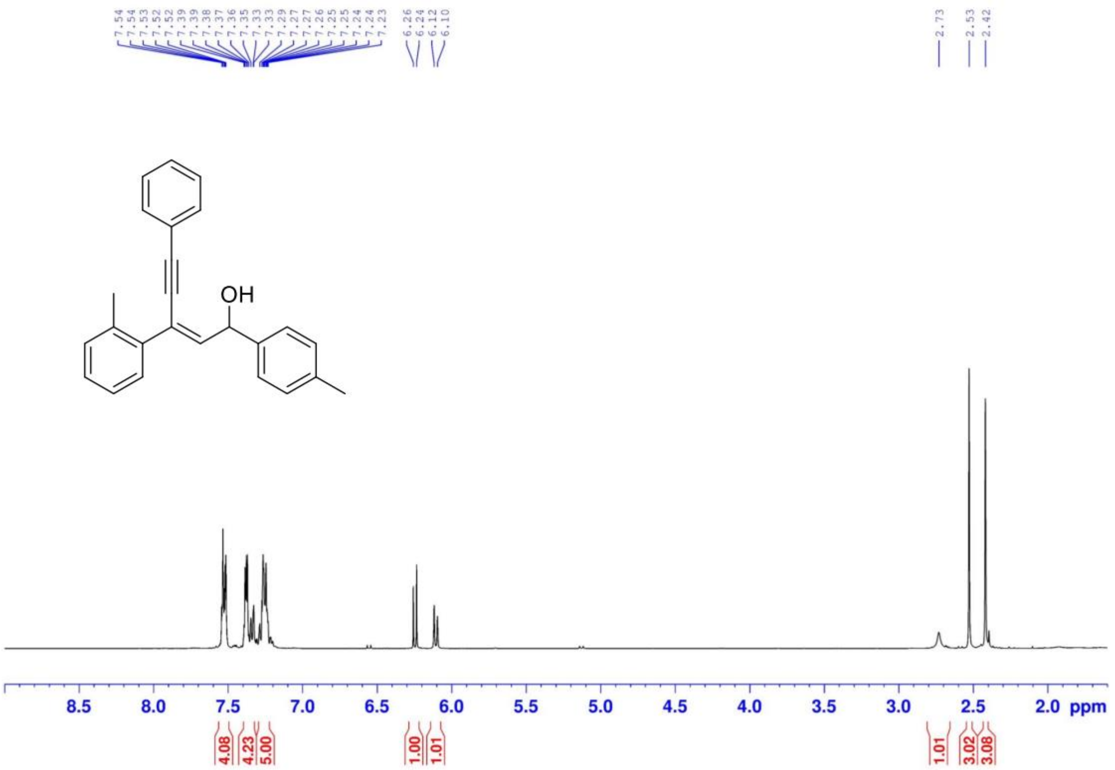

$^{13}C\{^{1}H\}$ NMR (100 MHz, $CDCl_3$) Spectrum of **1p**

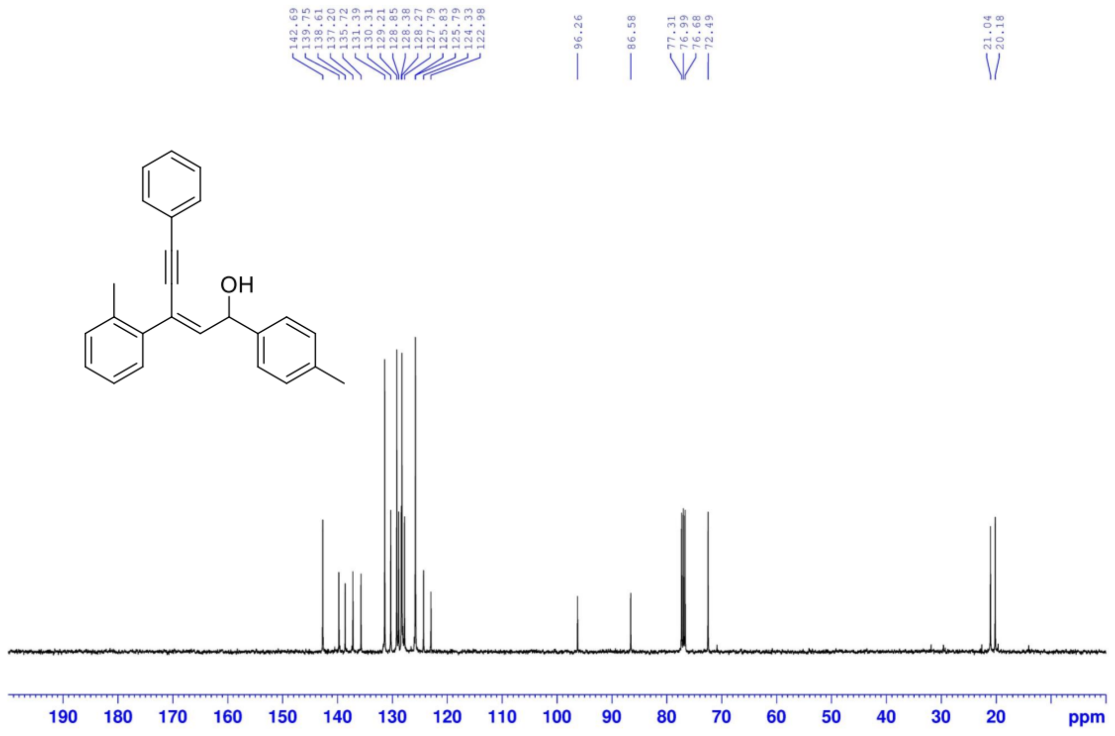

^{1}H NMR (400 MHz, $CDCl_3$) Spectrum of **2a**

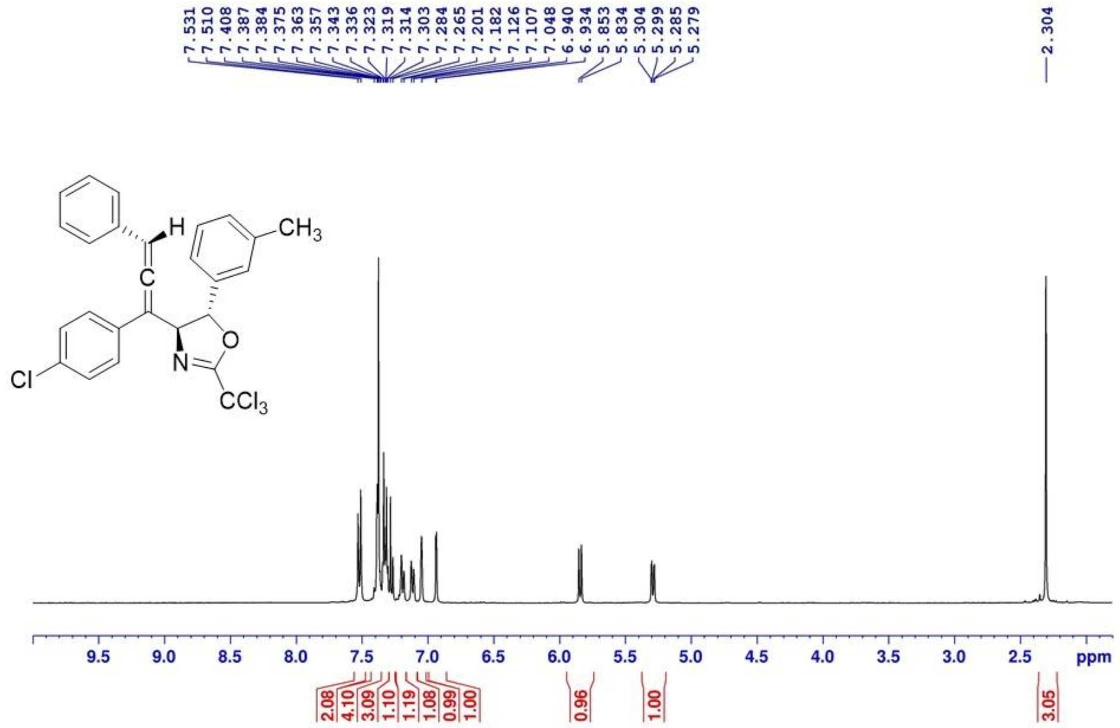

^{13}C{^{1}H} NMR (100 MHz, $CDCl_3$) Spectrum of **2a**

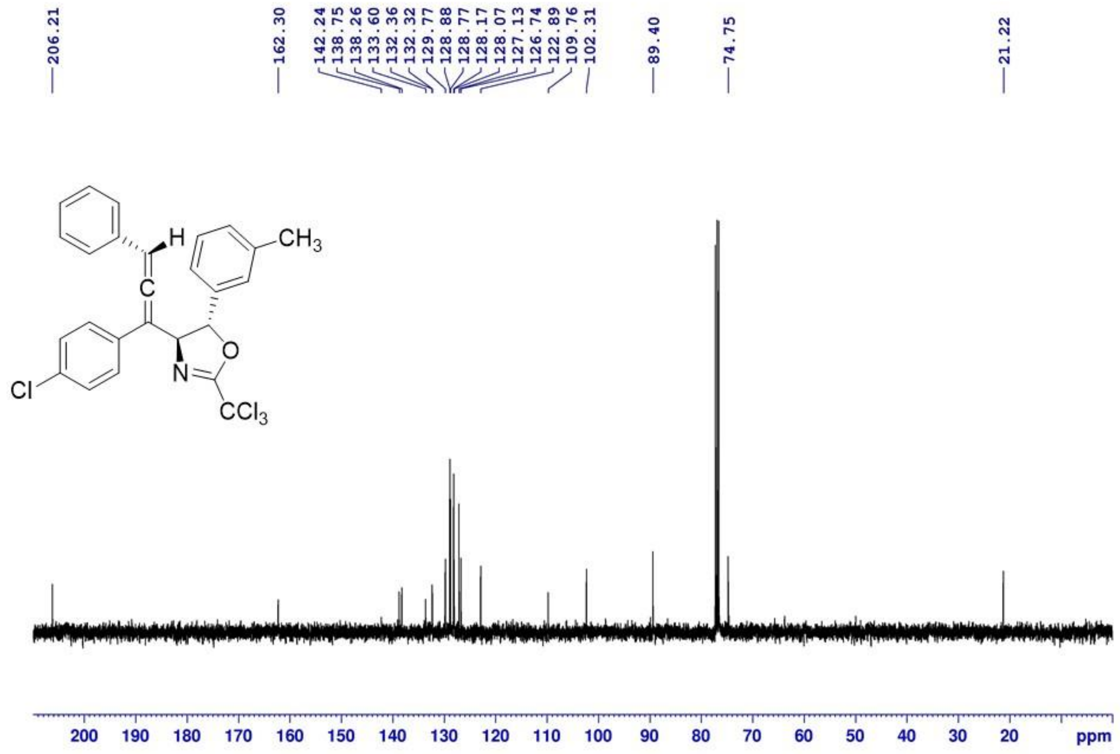

^{1}H NMR (400 MHz, $CDCl_3$) Spectrum of **2b**

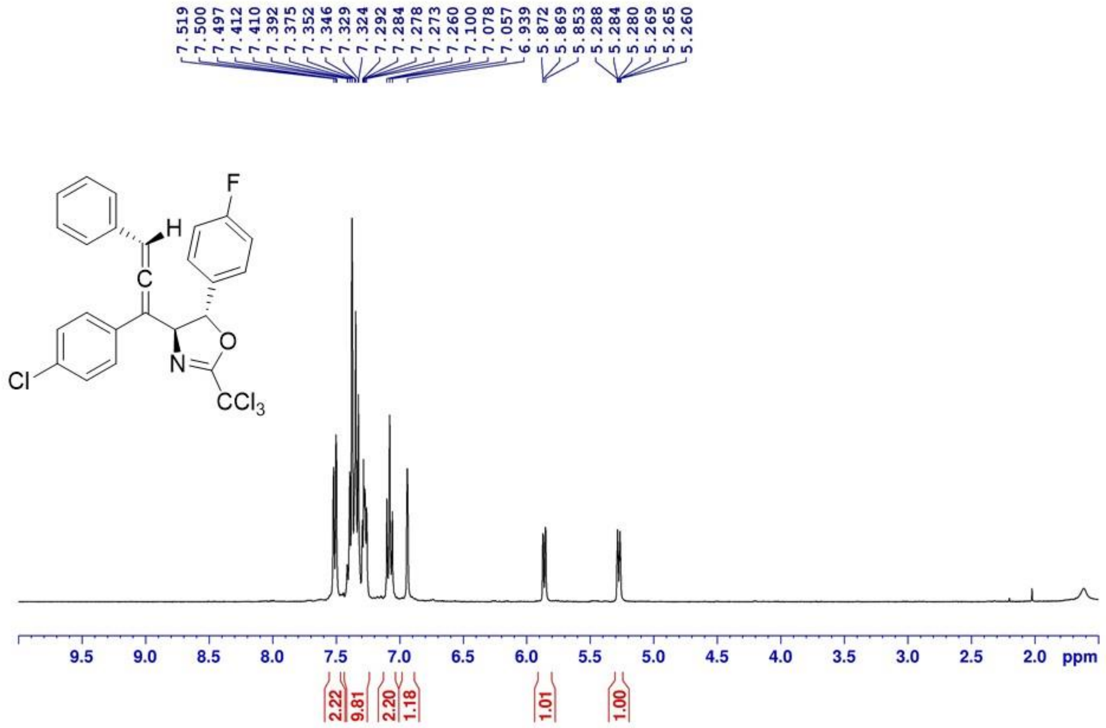

^{13}C{^{1}H} NMR (100 MHz, $CDCl_3$) Spectrum of **2b**

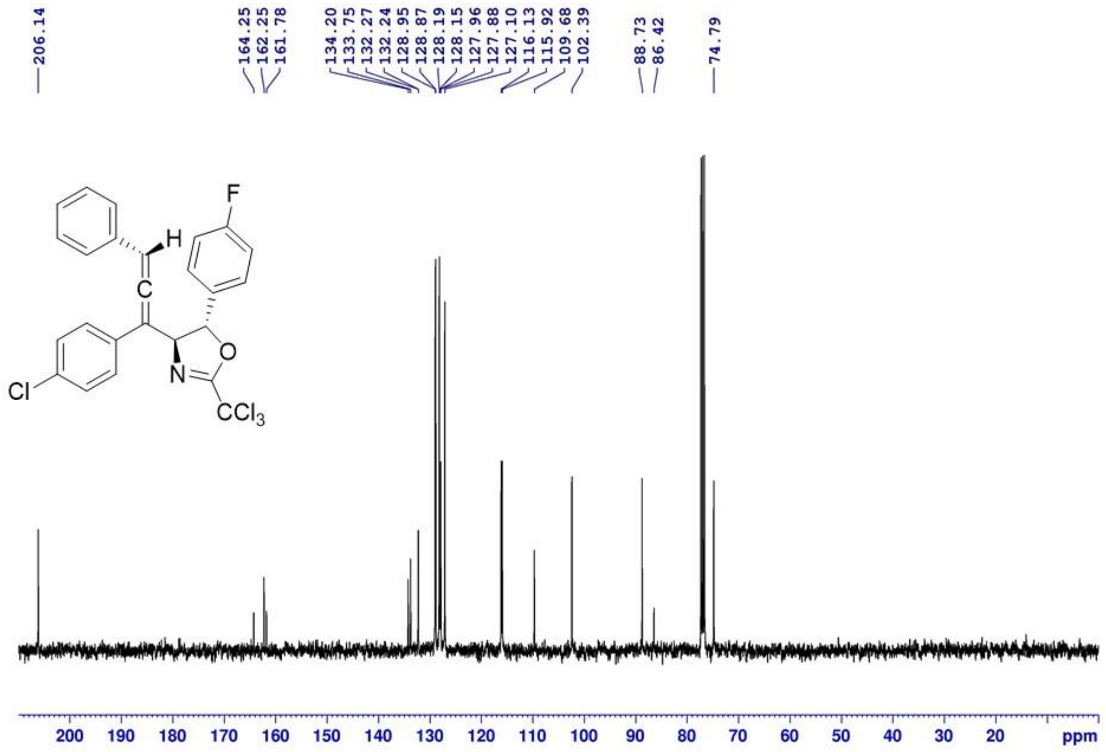

^{1}H NMR (400 MHz, $CDCl_3$) Spectrum of **2c**

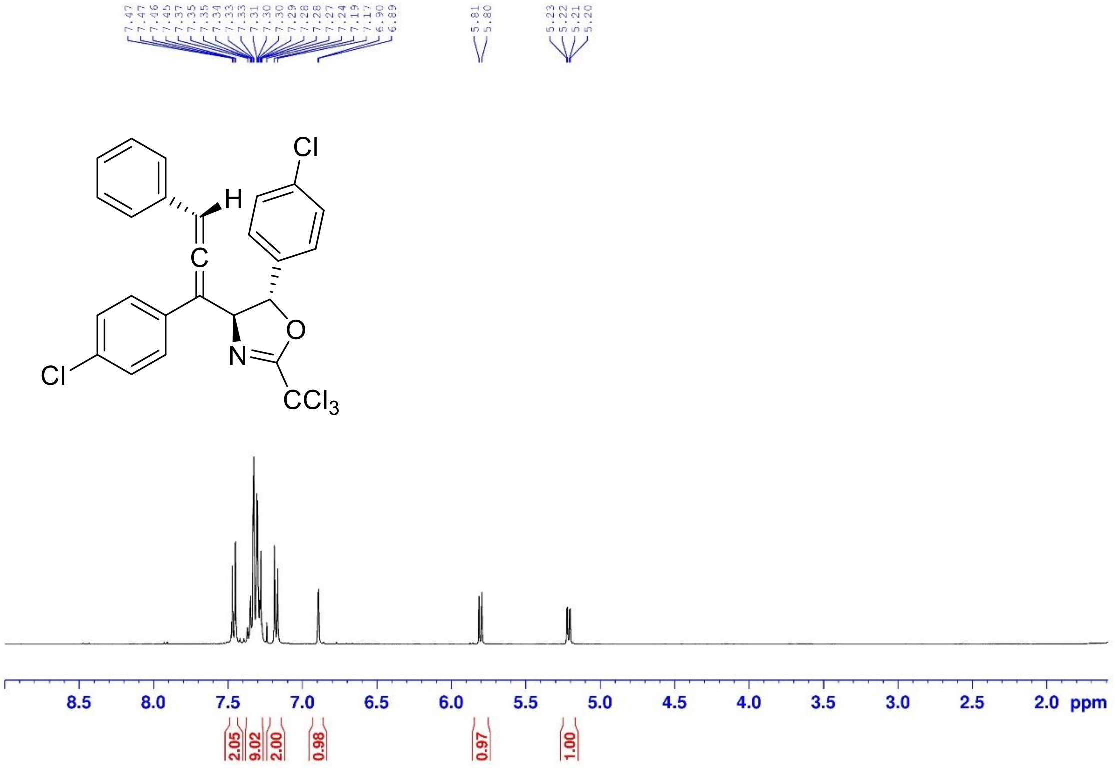

^{13}C{^{1}H} NMR (100 MHz, $CDCl_3$) Spectrum of **2c**

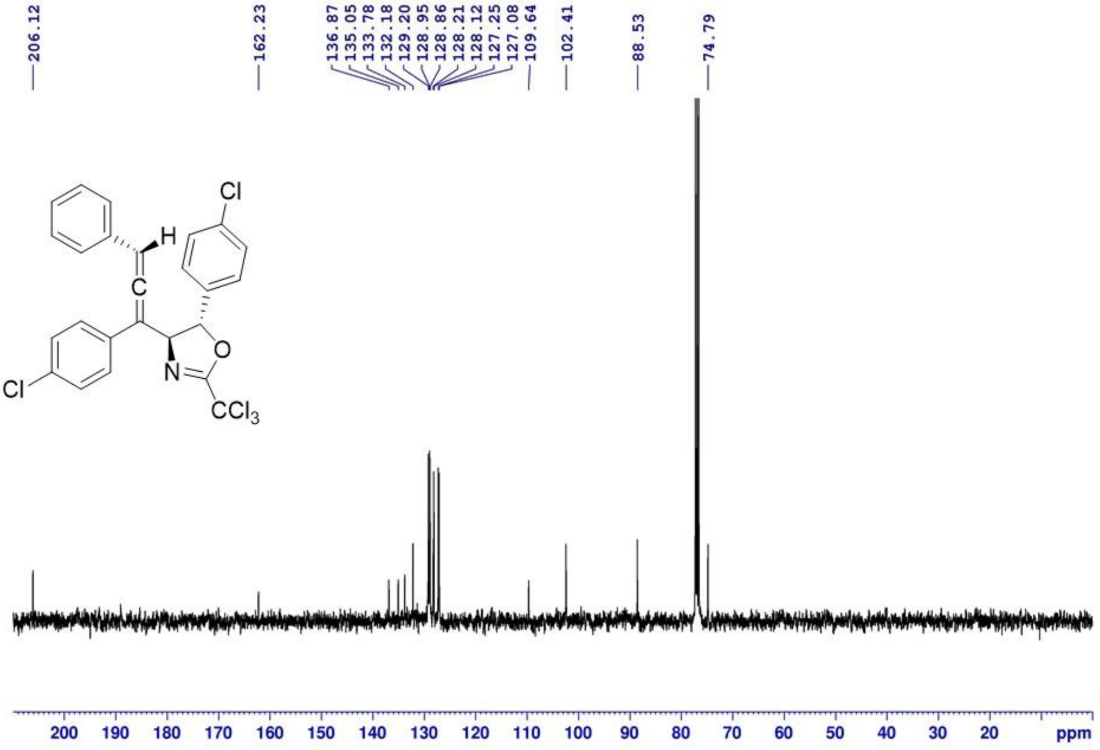

^{1}H NMR (400 MHz, $CDCl_3$) Spectrum of **2d**

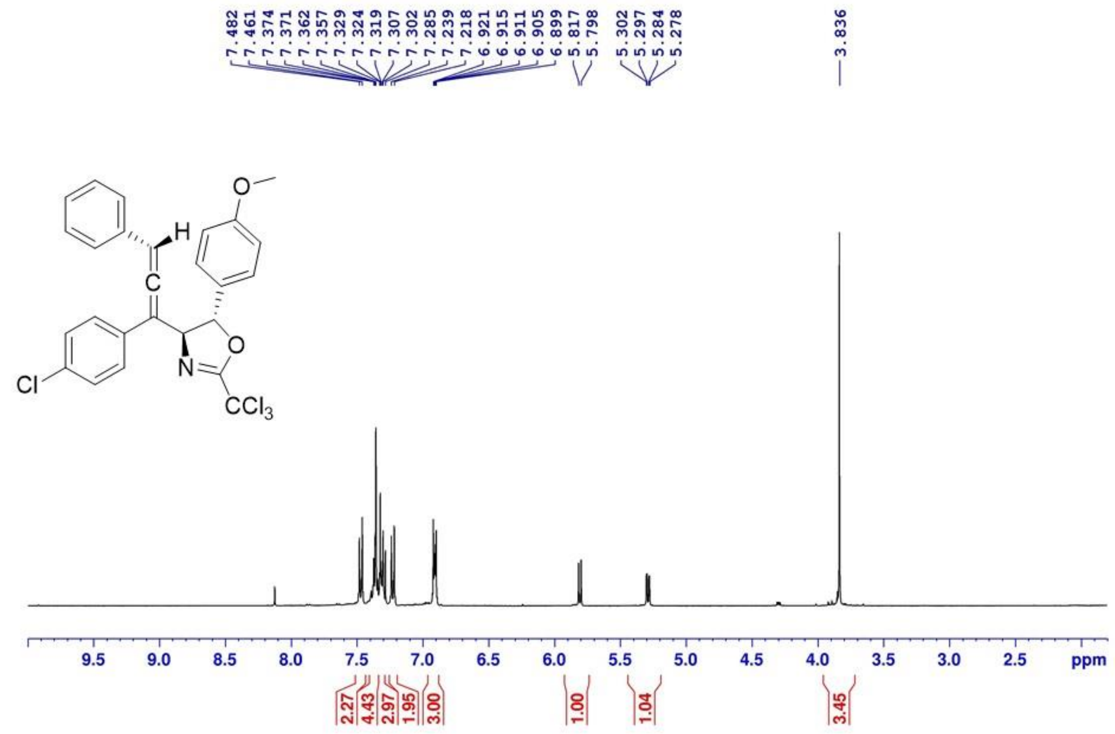

$^{13}C\{^{1}H\}$ NMR (100 MHz, $CDCl_3$) Spectrum of **2d**

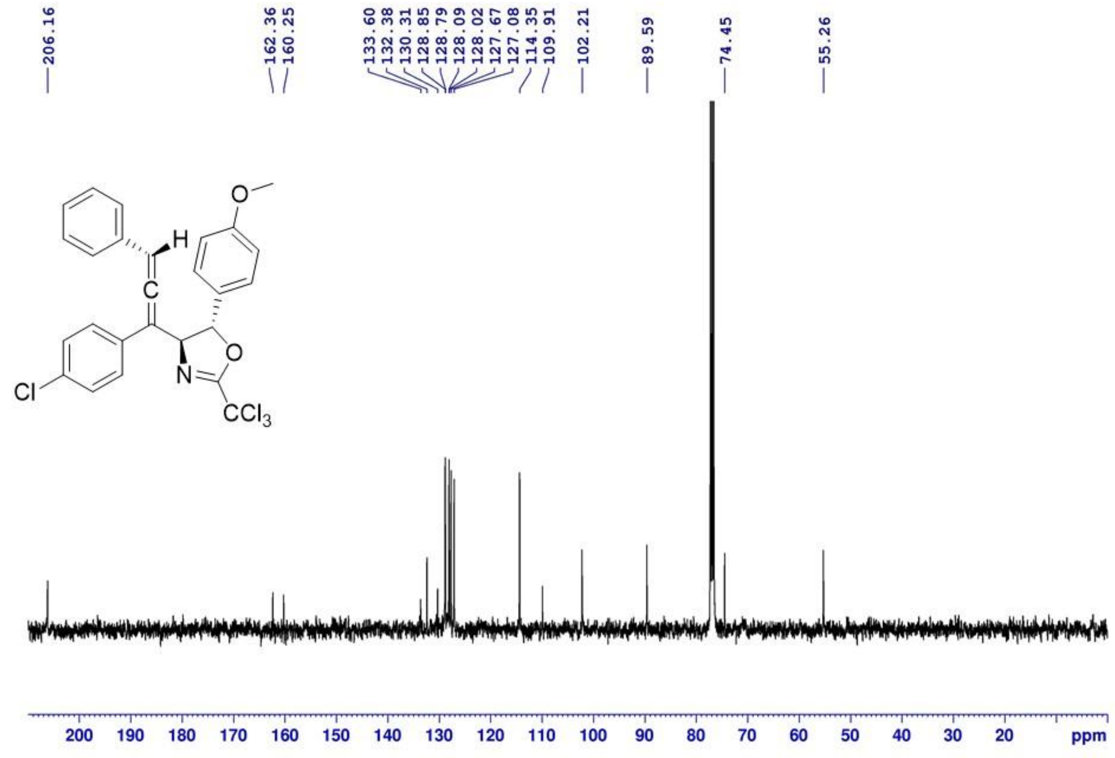

^{1}H NMR (400 MHz, $CDCl_3$) Spectrum of **2e**

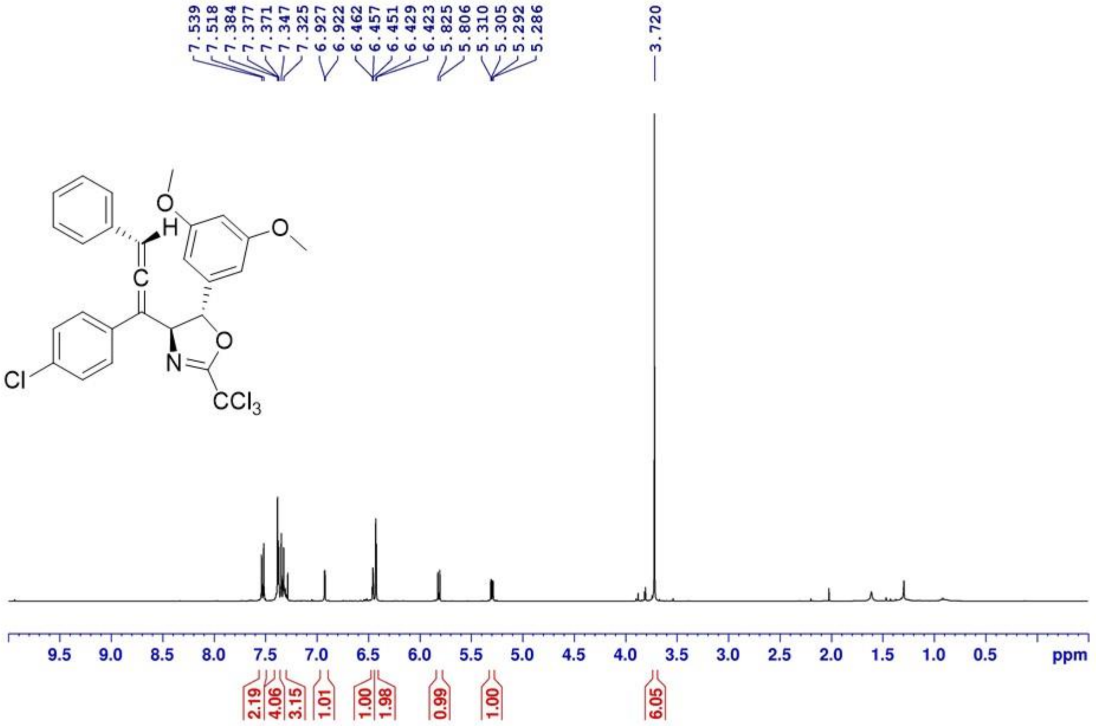

$^{13}C\{^{1}H\}$ NMR (100 MHz, $CDCl_3$) Spectrum of **2e**

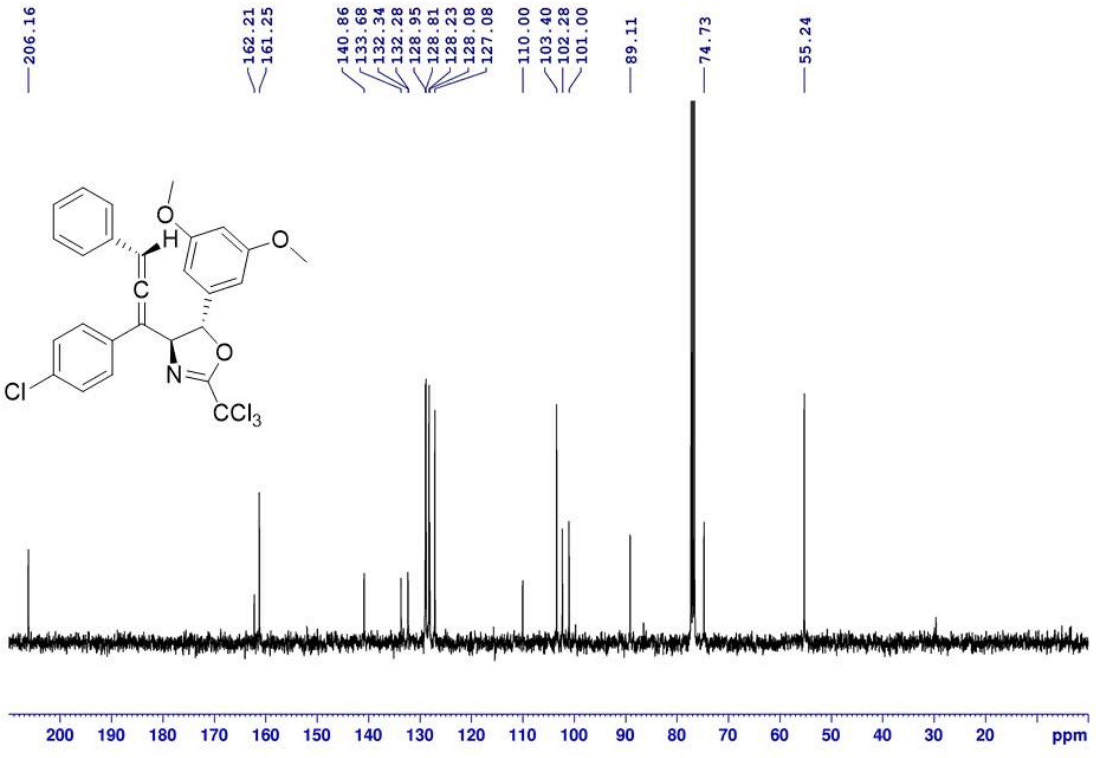

^{1}H NMR (400 MHz, $CDCl_3$) Spectrum of **2f**

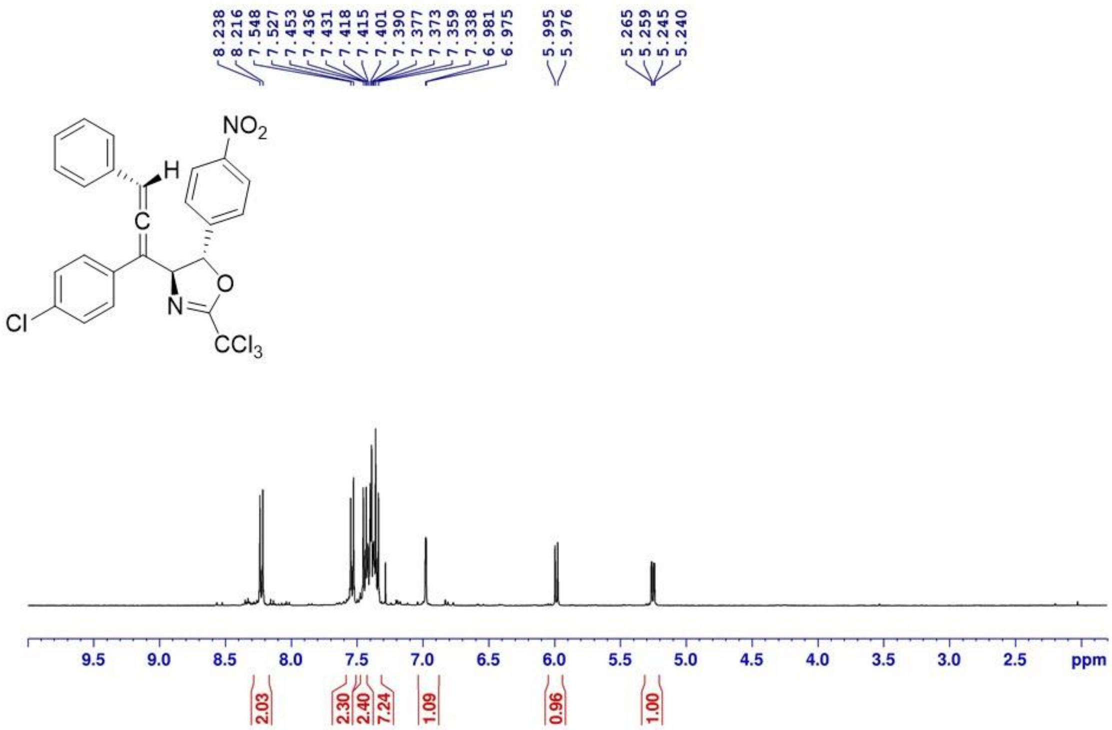

$^{13}C\{^{1}H\}$ NMR (100 MHz, $CDCl_3$) Spectrum of **2f**

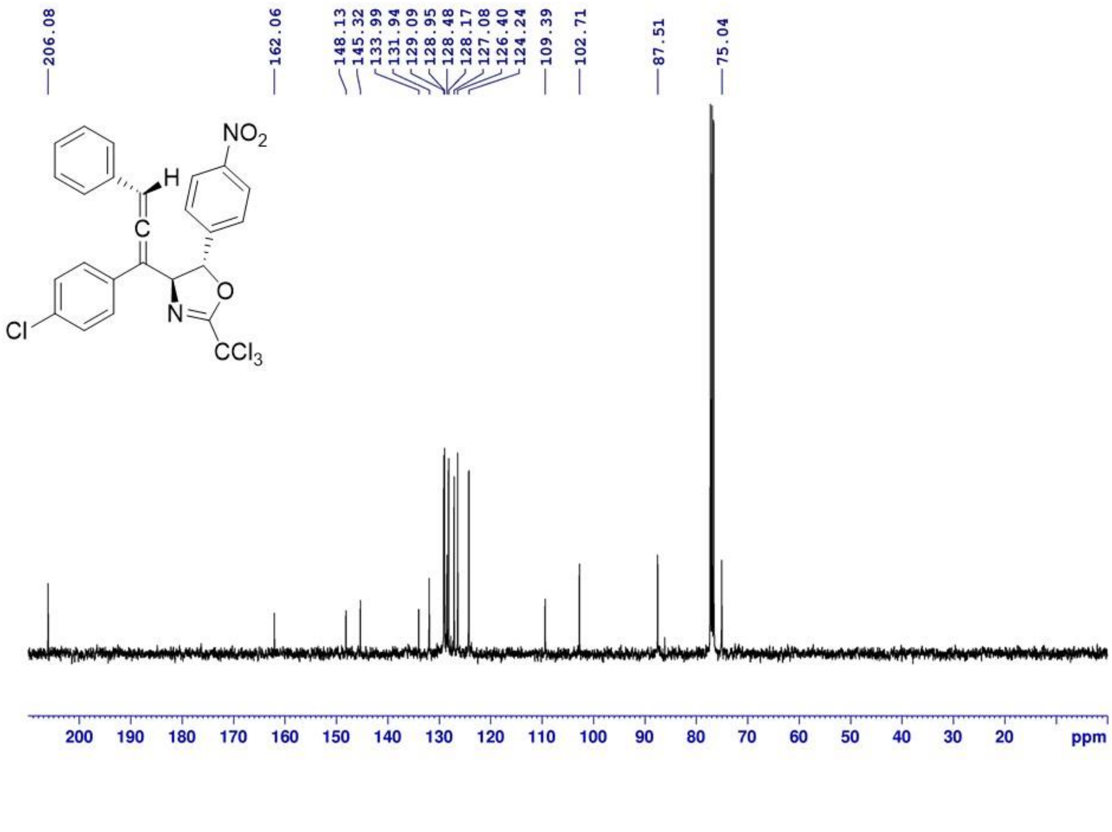

^{1}H NMR (400 MHz, $CDCl_3$) Spectrum of **2g**

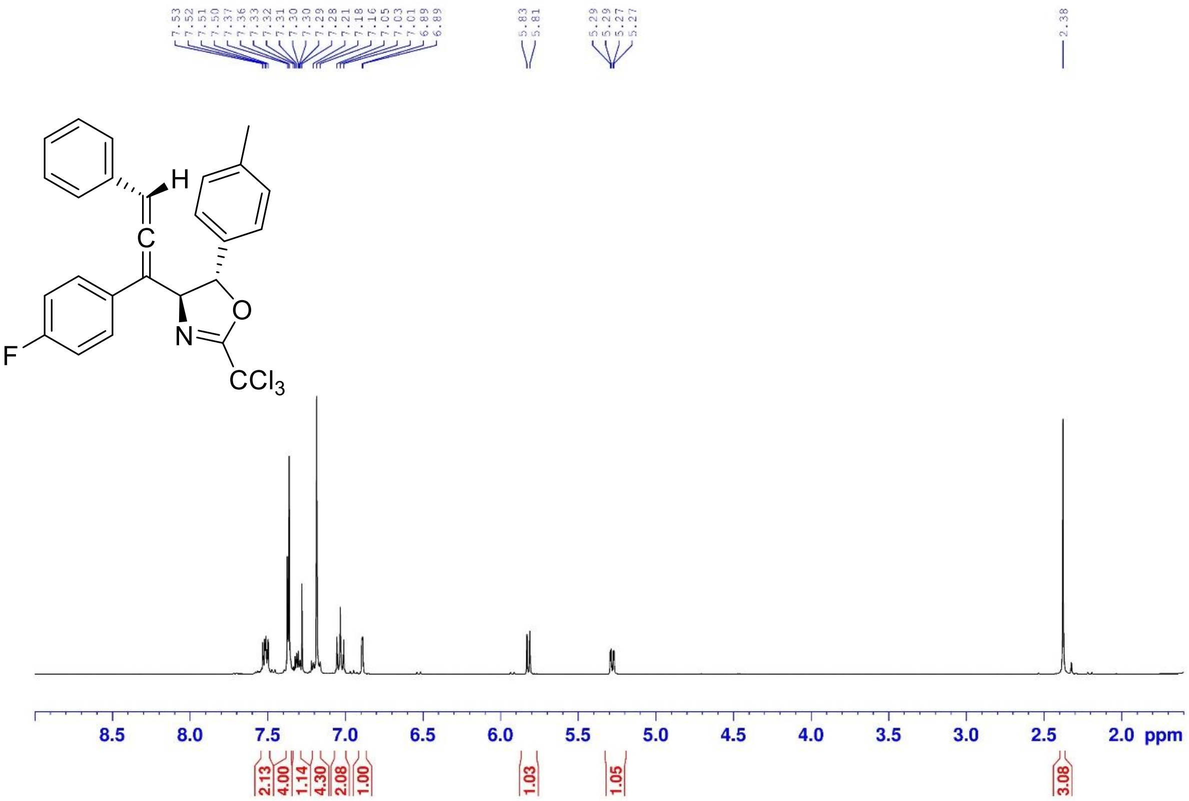

$^{13}C\{^{1}H\}$ NMR (100 MHz, $CDCl_3$) Spectrum of **2g**

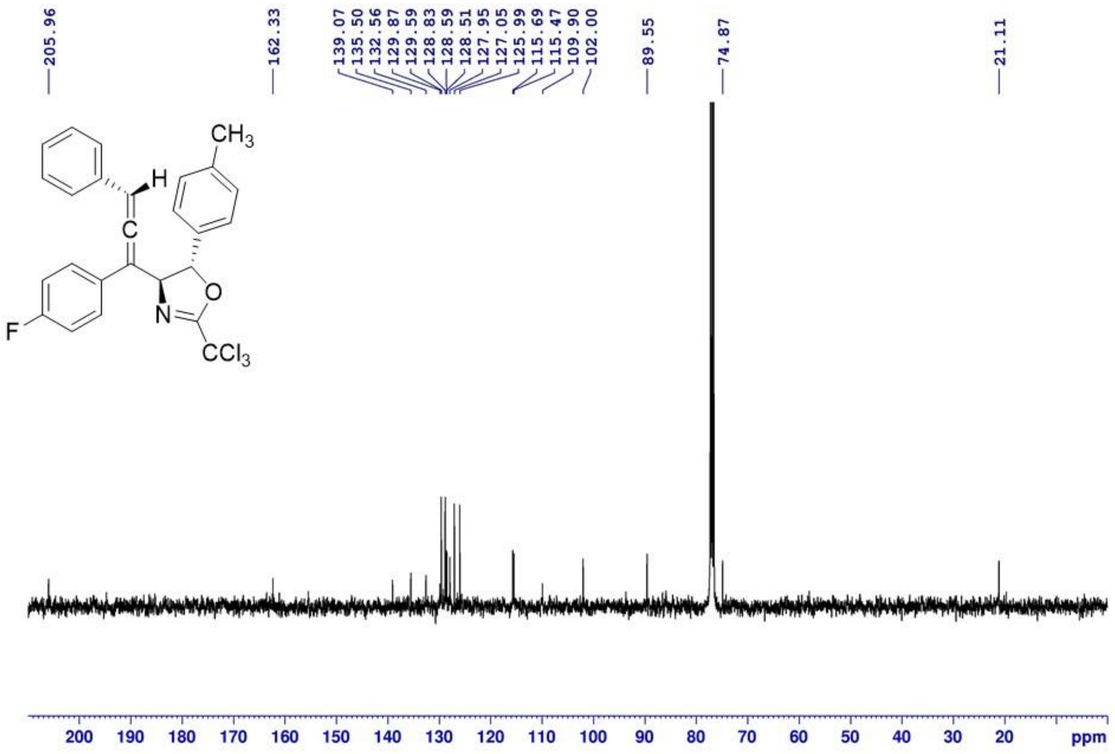

^{1}H NMR (400 MHz, $CDCl_3$) Spectrum of **2h**

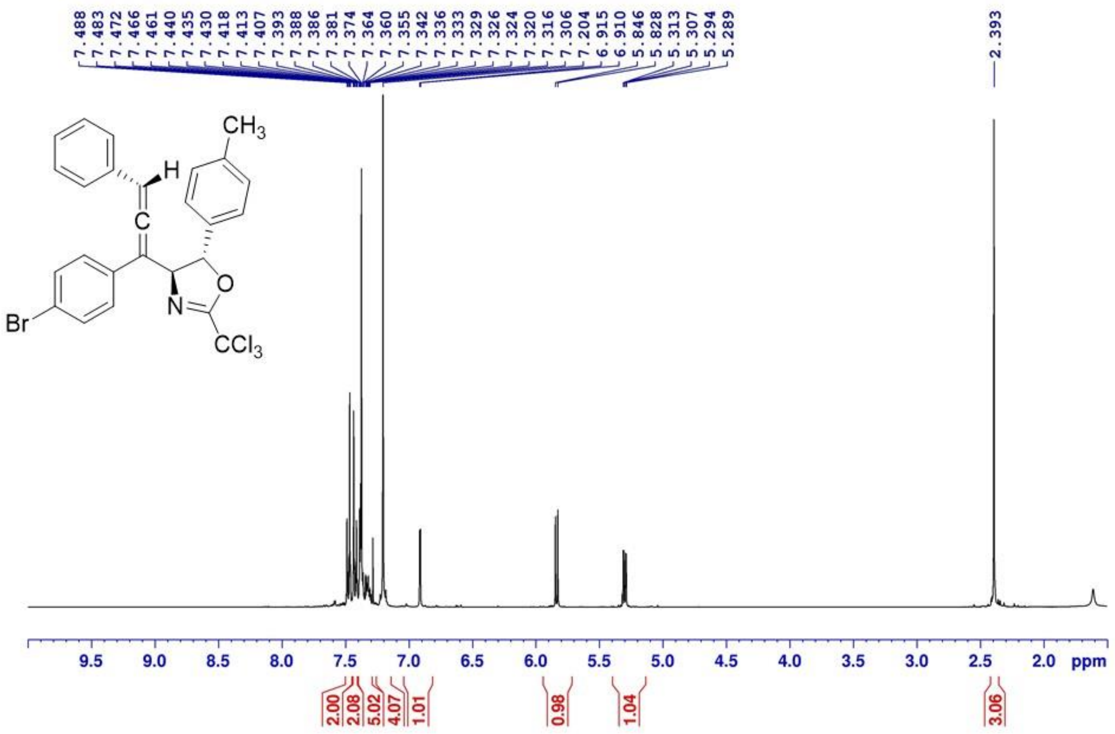

$^{13}C\{^{1}H\}$ NMR (100 MHz, $CDCl_3$) Spectrum of **2h**

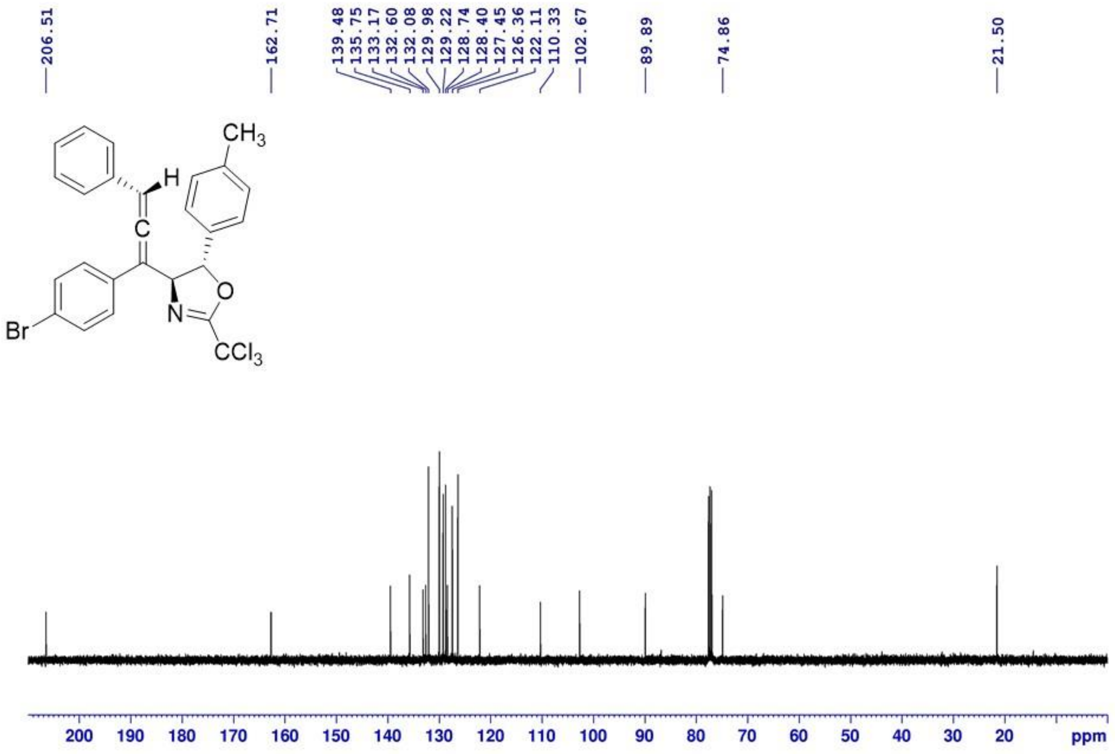

^{1}H NMR (400 MHz, $CDCl_3$) Spectrum of **2o**

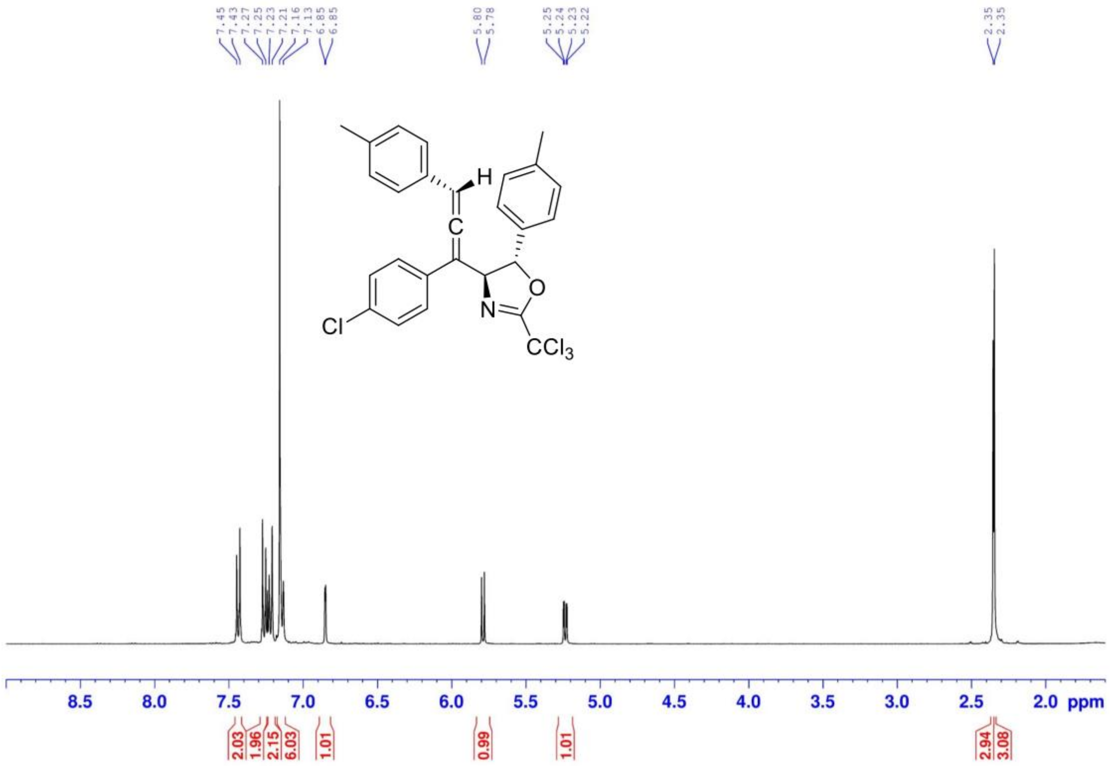

$^{13}C\{^{1}H\}$ NMR (100 MHz, $CDCl_3$) Spectrum of **2o**

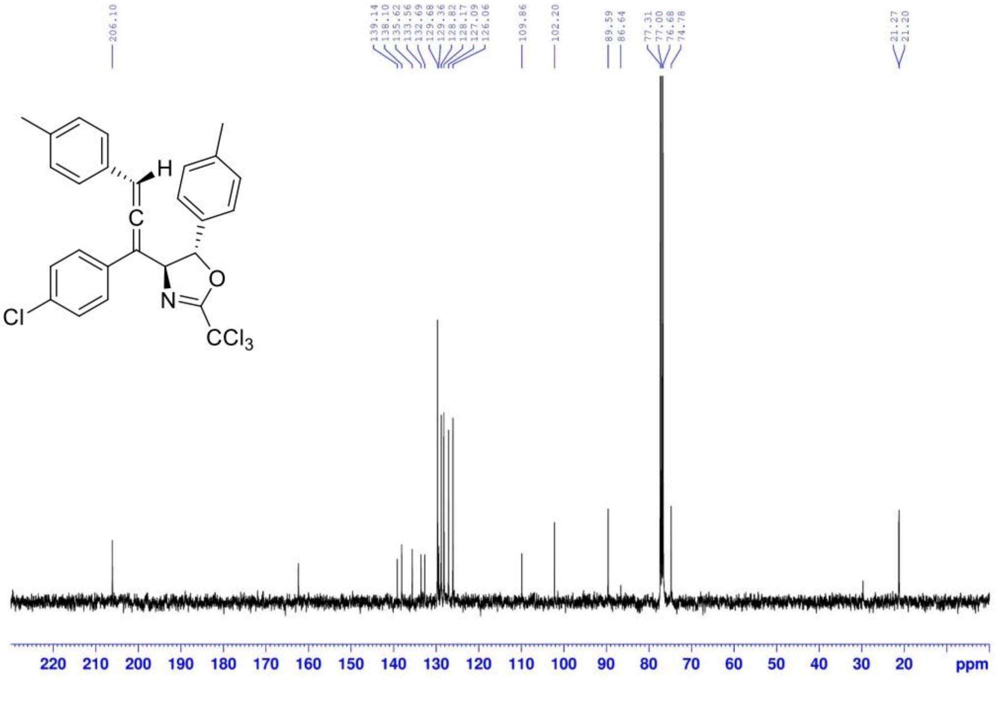

^{1}H NMR (400 MHz, $CDCl_3$) Spectrum of **3**

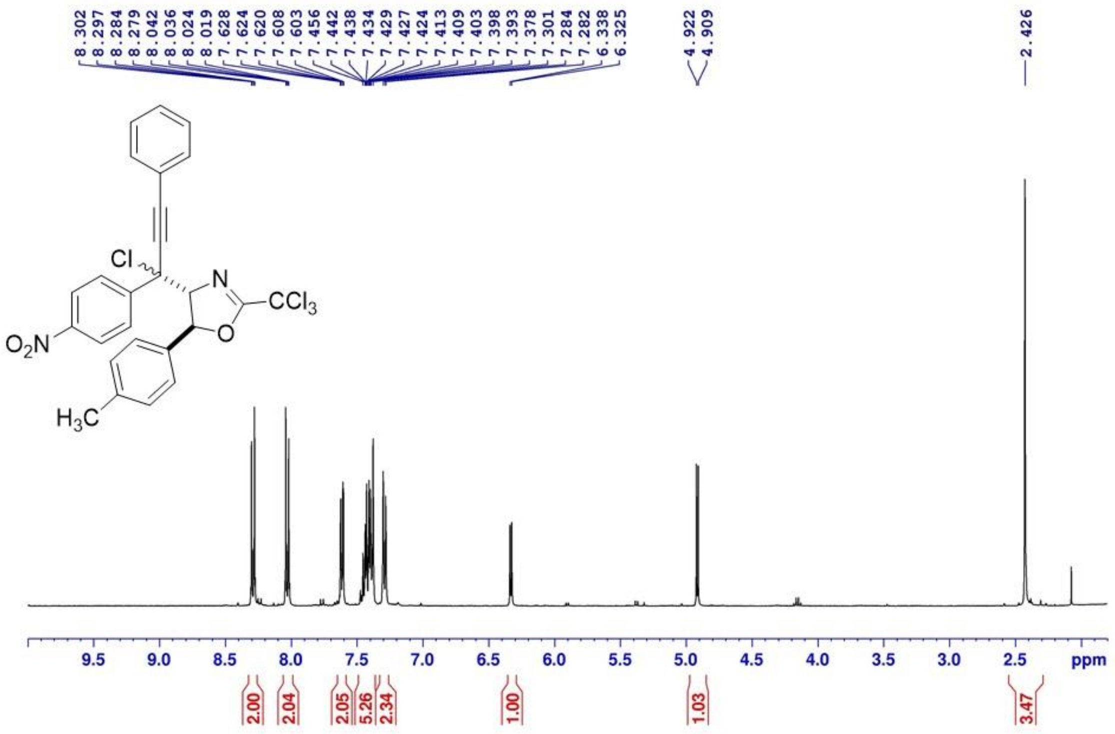

$^{113}C\{^{1}H\}$ NMR (100 MHz, $CDCl_3$) Spectrum of **3**

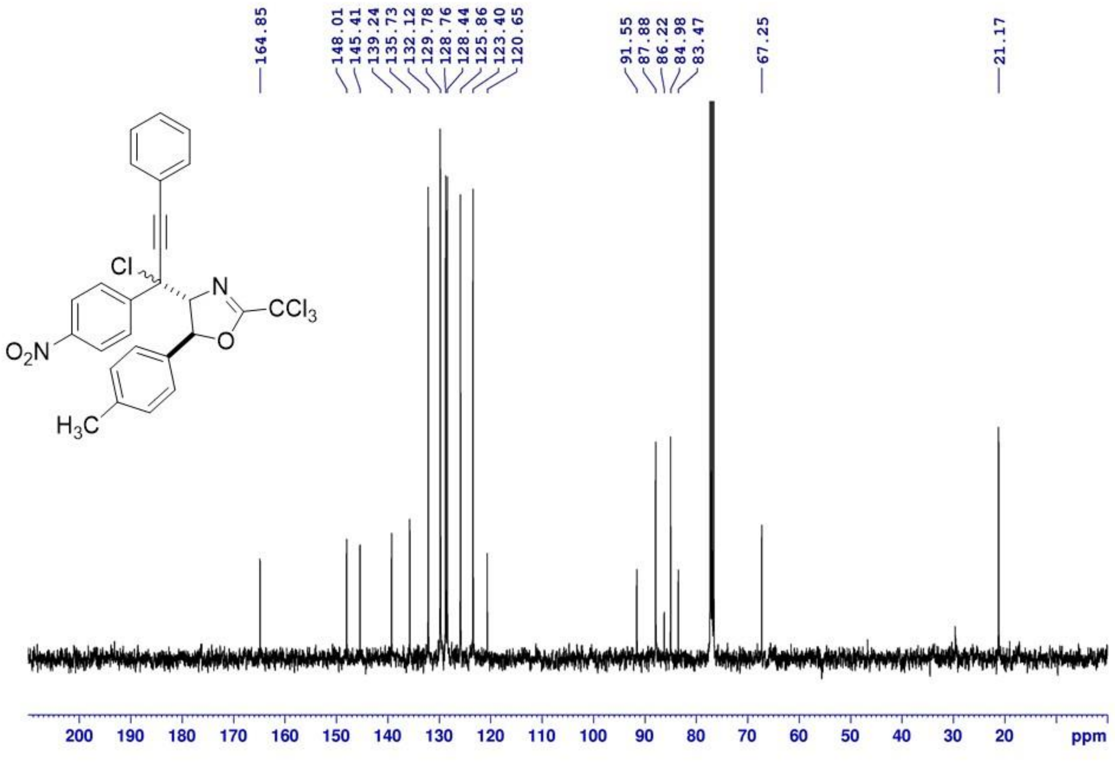

^1H NMR (400 MHz, $CDCl_3$) Spectrum of **4**

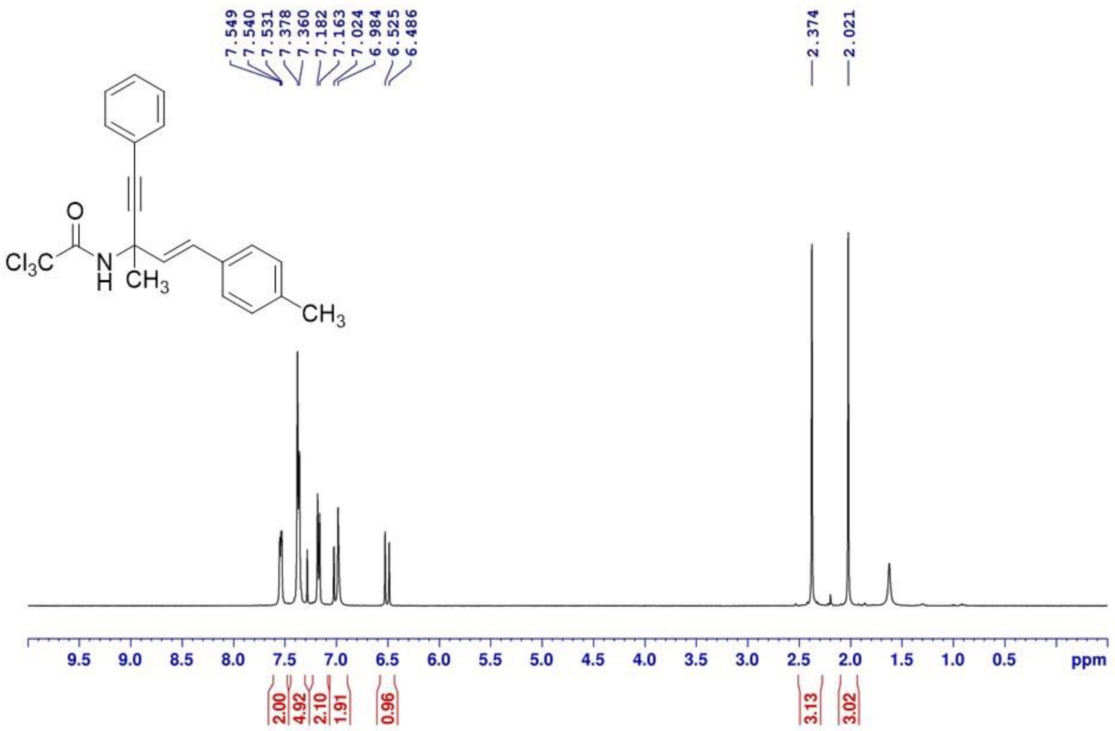

$^{13}C\{^1H\}$ NMR (100 MHz, $CDCl_3$) Spectrum of **4**

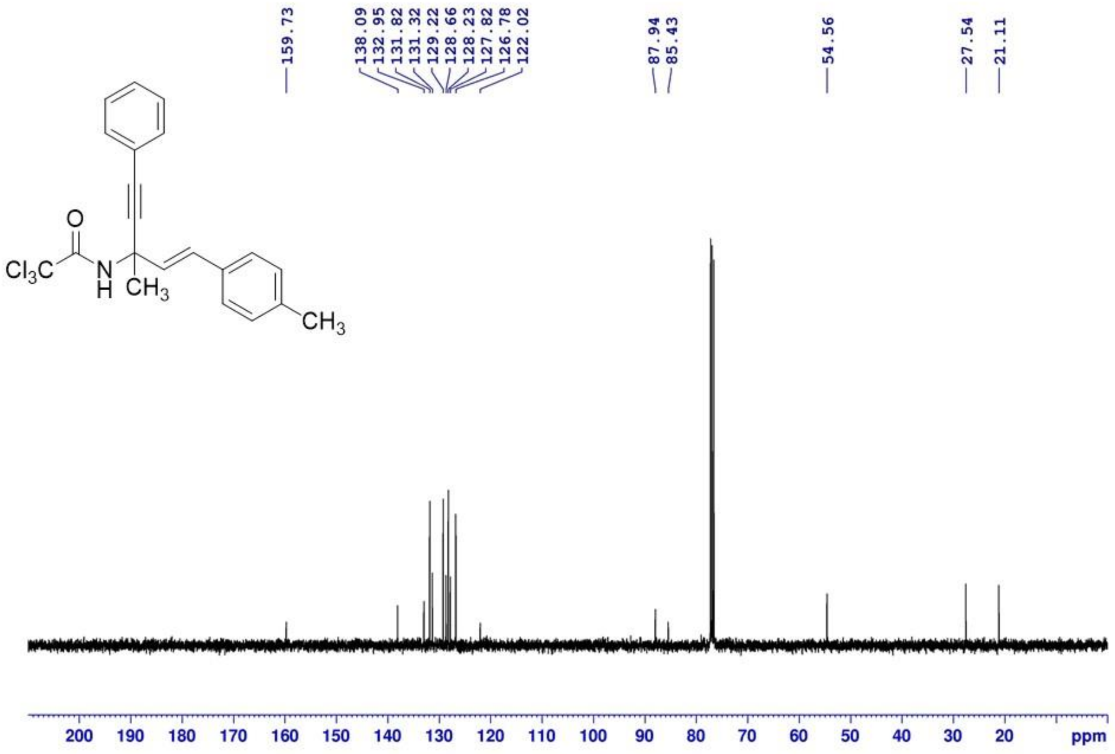

^{1}H NMR (400 MHz, $CDCl_3$) Spectrum of **5**

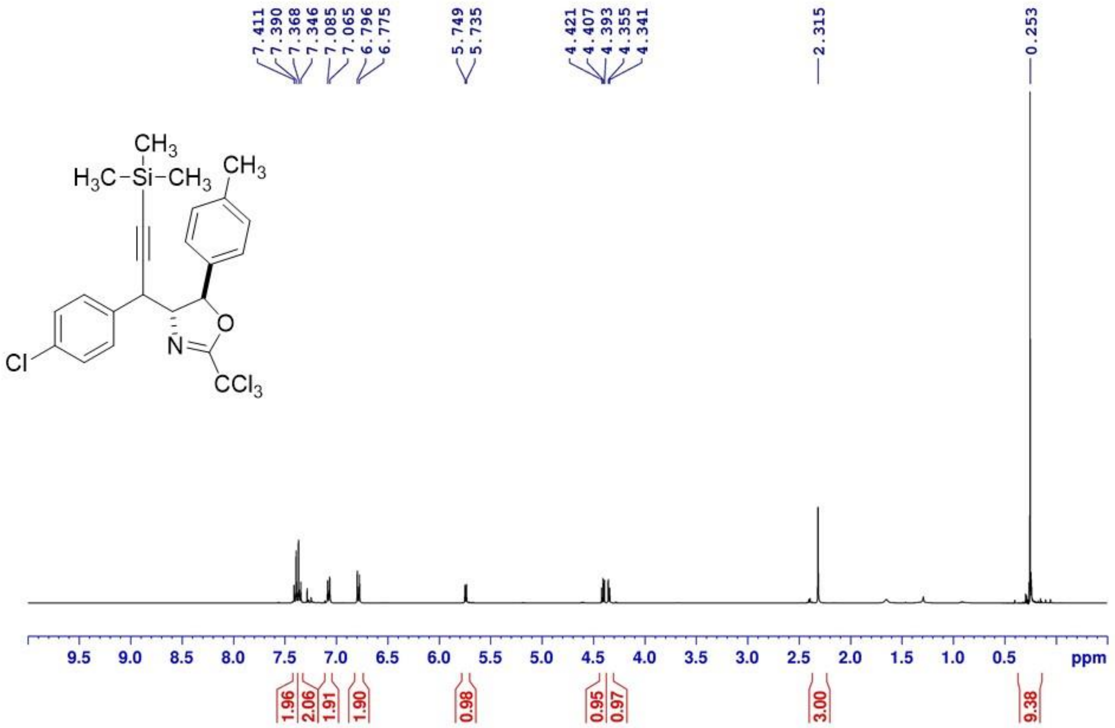

$^{13}C\{^{1}H\}$ NMR (100 MHz, $CDCl_3$) Spectrum of **5**

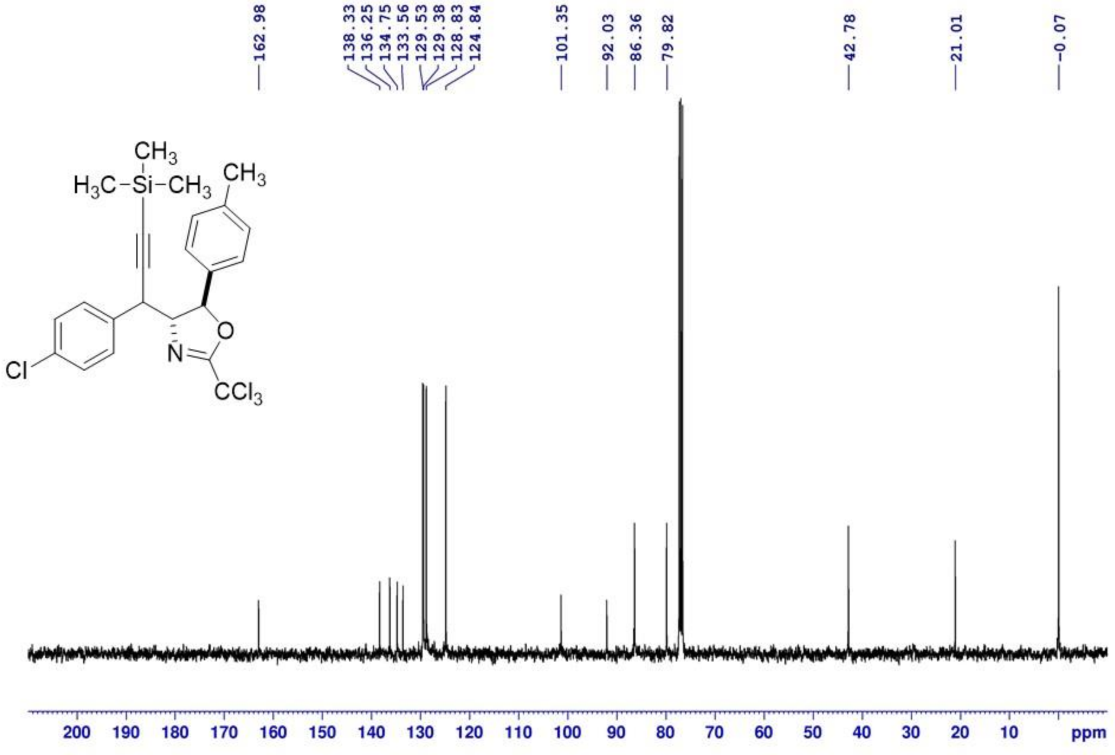

^{1}H NMR (400 MHz, $CDCl_3$) Spectrum of **6a**

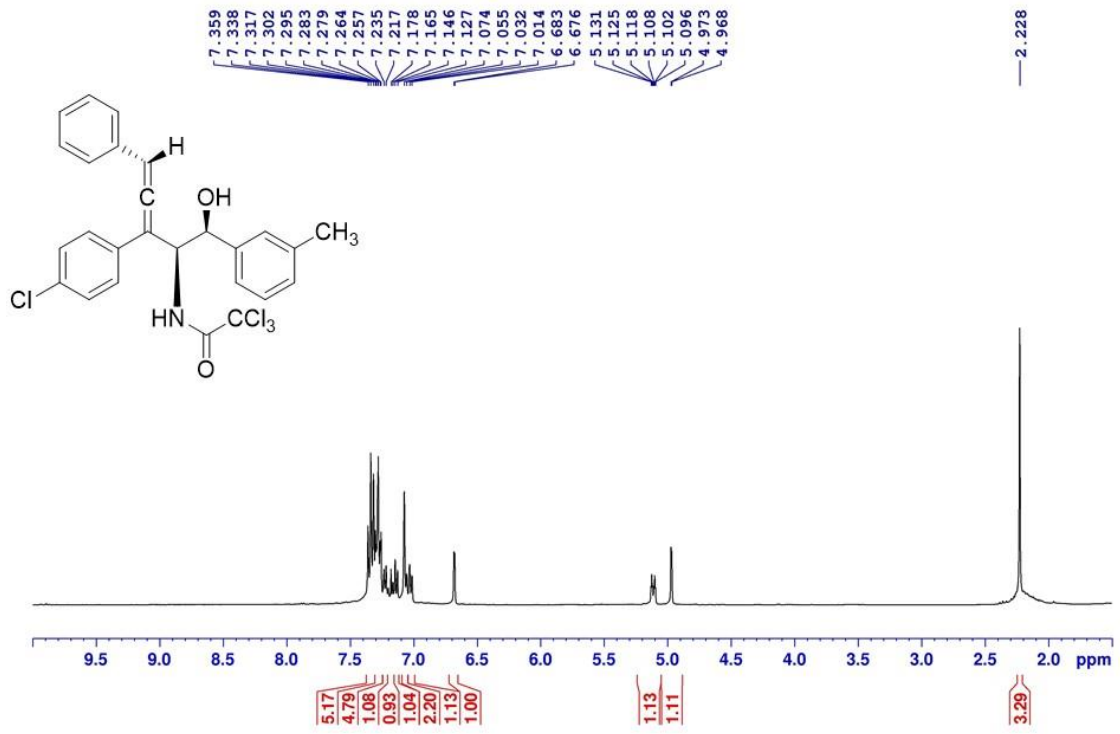

$^{13}C\{^{1}H\}$ NMR (100 MHz, $CDCl_3$) Spectrum of **6a**

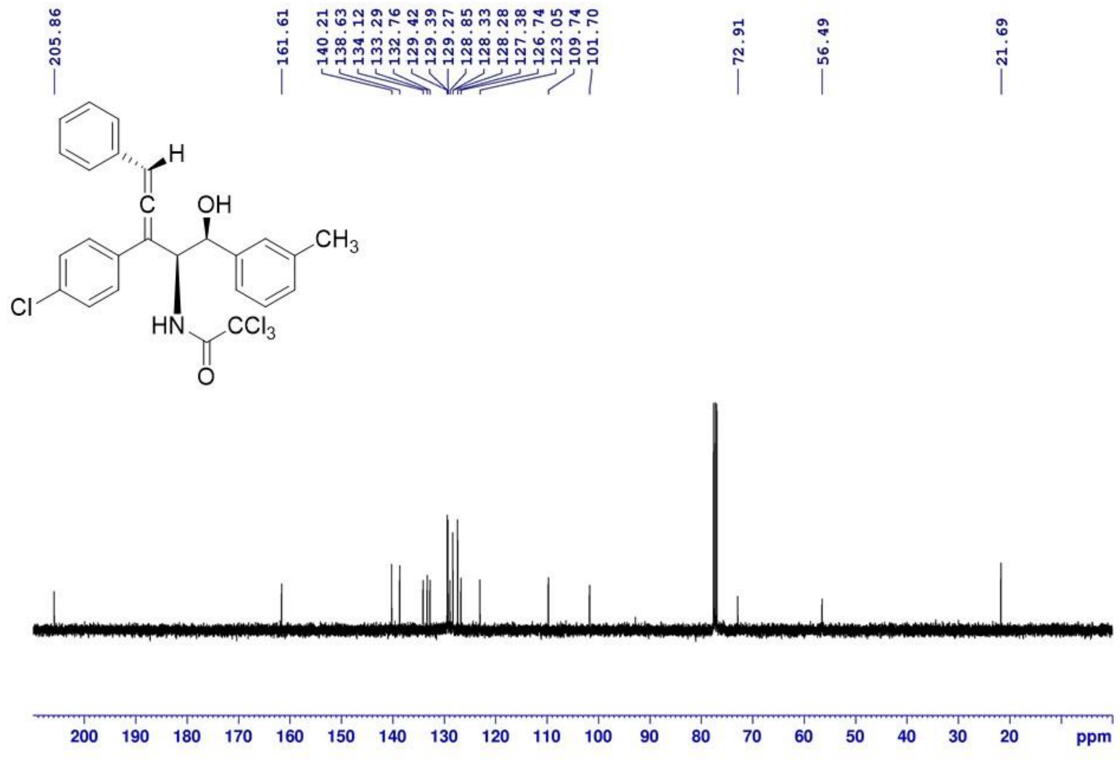

^{1}H NMR (400 MHz, $CDCl_3$) Spectrum of **6b**

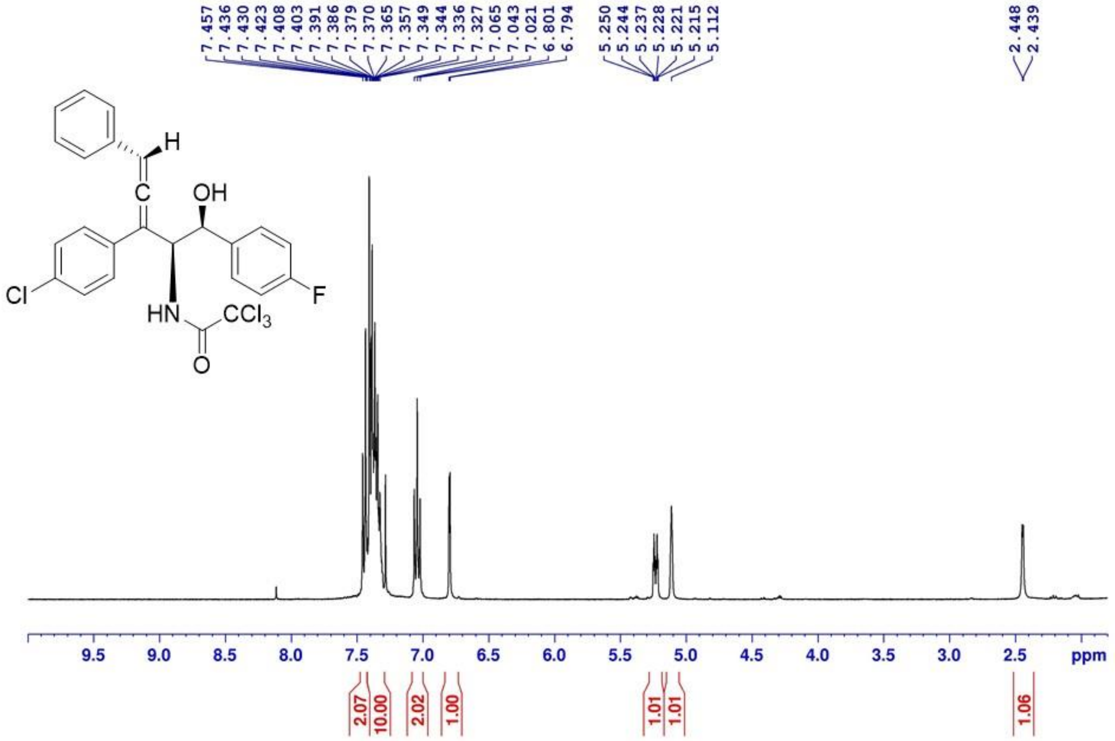

$^{13}C\{^{1}H\}$ NMR (100 MHz, $CDCl_3$) Spectrum of **6b**

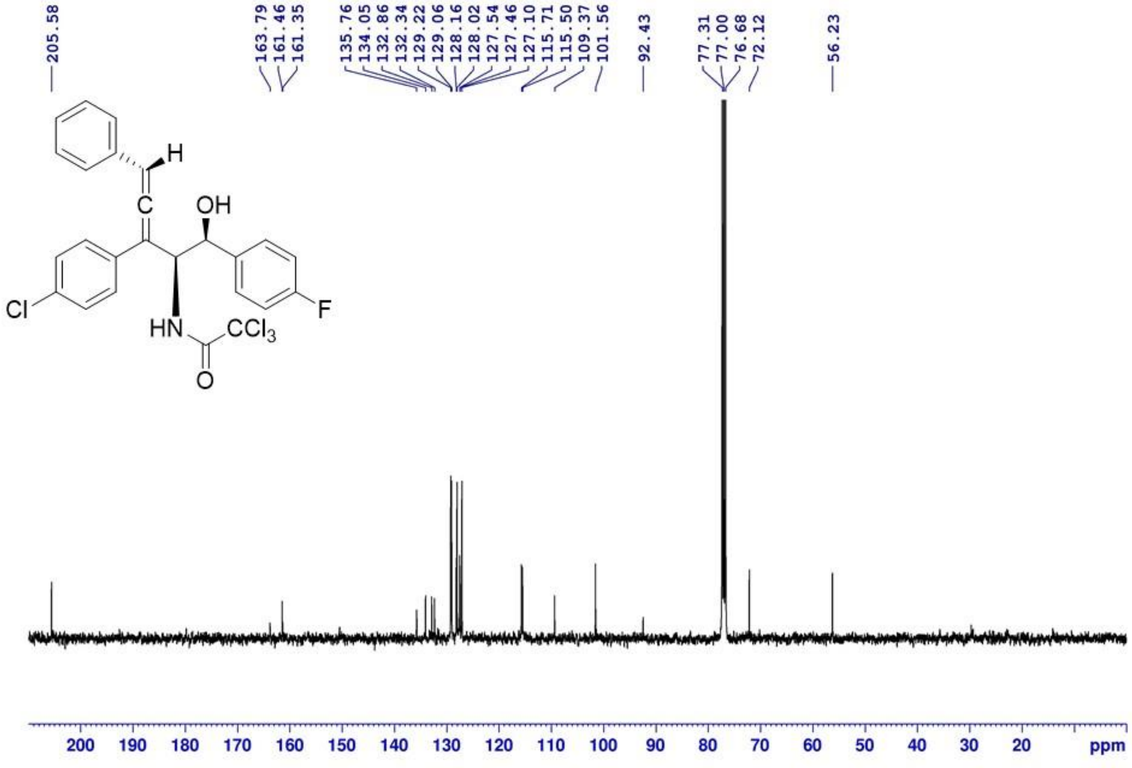

^{1}H NMR (400 MHz, $CDCl_3$) Spectrum of **6c**

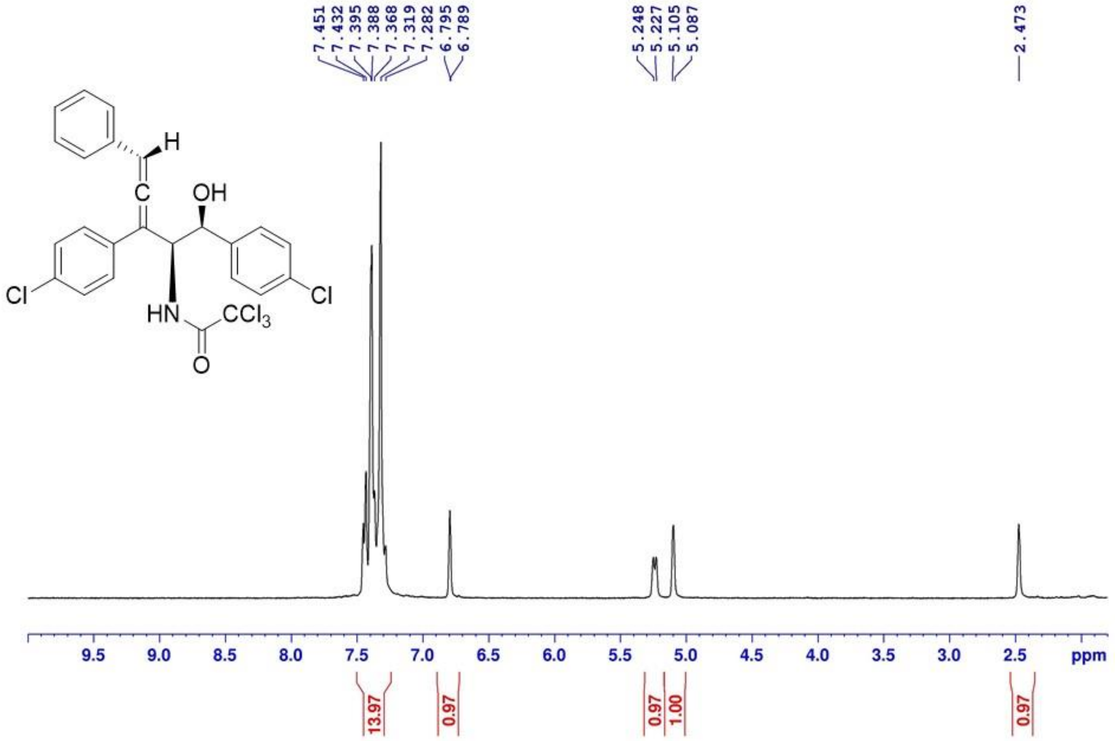

$^{13}C\{^{1}H\}$ NMR (100 MHz, $CDCl_3$) Spectrum of **6c**

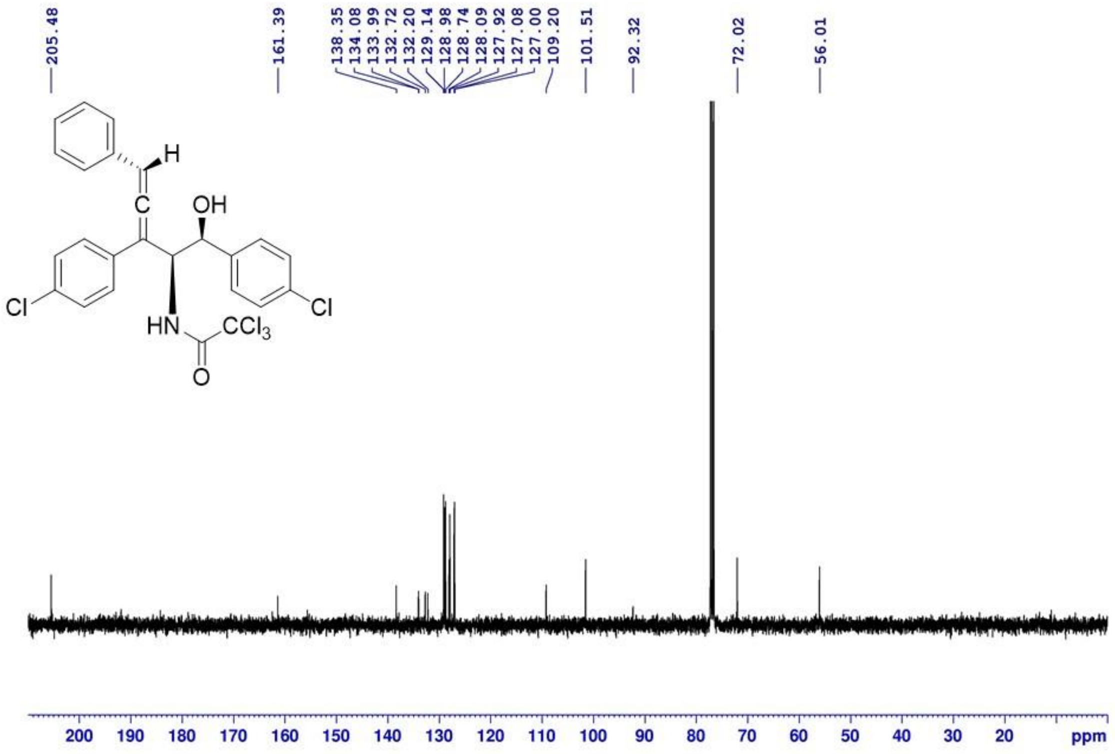

^{1}H NMR (400 MHz, $CDCl_3$) Spectrum of **6h**

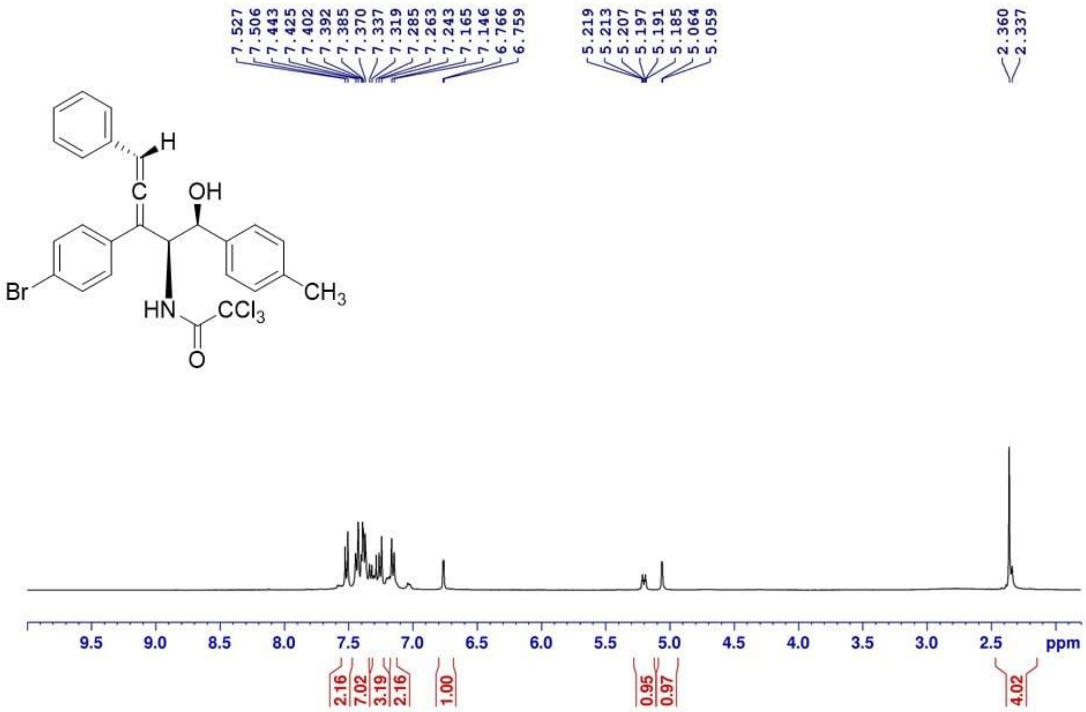

$^{13}C\{^{1}H\}$ NMR (100 MHz, $CDCl_3$) Spectrum of **6h**

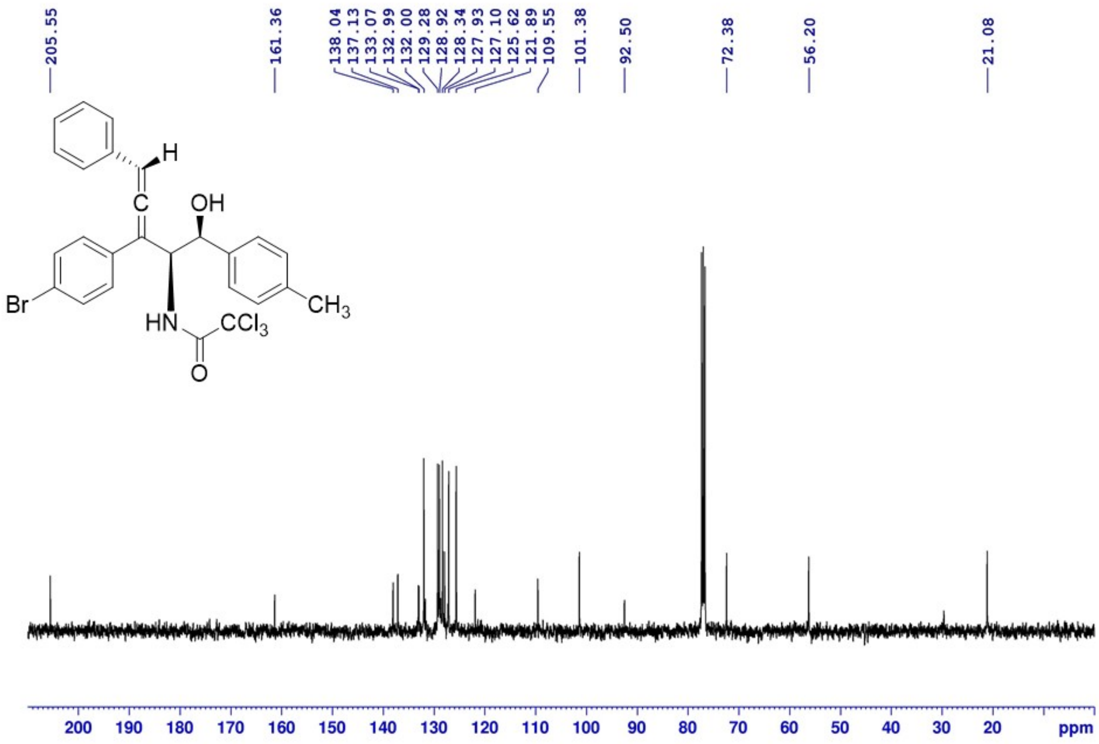

Figure S3. NOE signal enhancement (%) of **2a**

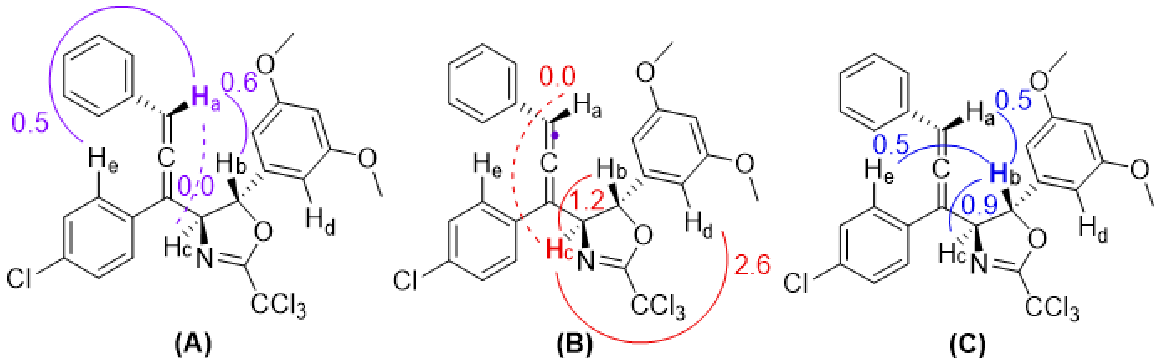

Figure S4. Mass spectrum of compound **3.**

Meas. m/z	#	Ion Formula	m/z	err [mDa]	err [ppm]	mSigma	Score	rdb	e⁻ Conf	N-Rule
547.0158	1	C26H19Cl4N2O3	547.0144	1.4	2.5	62.6	100.00	16.5	even	ok

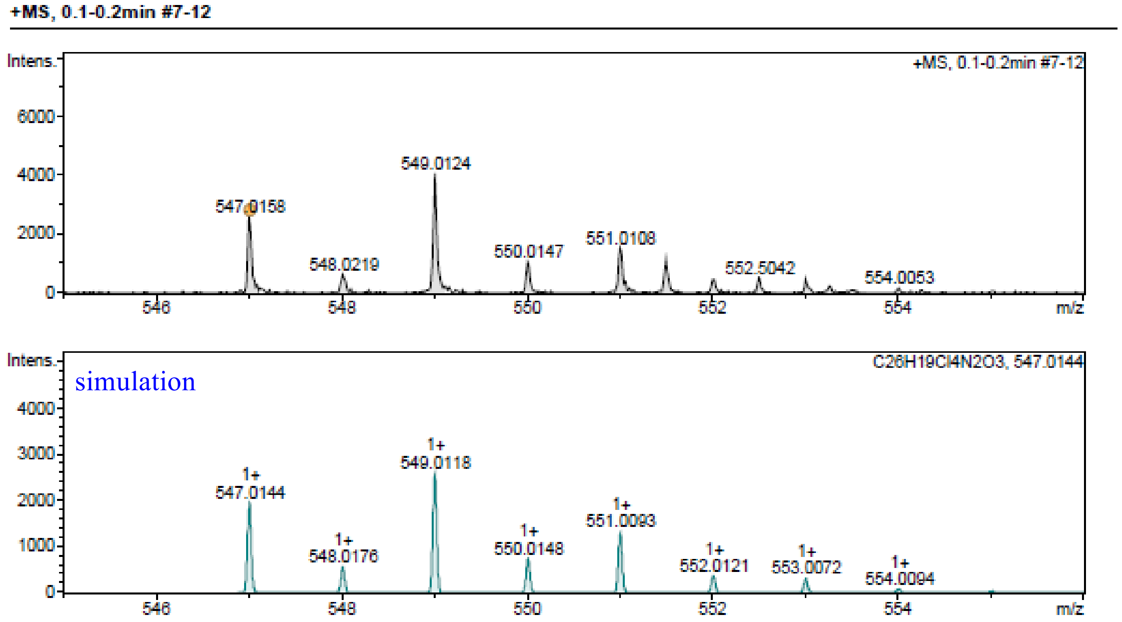

Supplement: Supplementary file 1 — jo4c01152_si_001.pdf [file jo4c01152_si_001.pdf]
